# Arbuscular mycorrhizal fungi and mycorrhizal helper bacteria co-inoculation alters drought-associated physiological traits and leaf abscisic acid concentration in citrus rootstocks

**DOI:** 10.1371/journal.pone.0361406

**Published:** 2026-10-27

**Authors:** Prananath Barman, Sanjay Kumar Singh, Raj Kumar, Daya Shankar Mishra, Anil Shukla, Avnish Kumar Pandey, Ali Khadivi, Yazgan Tunç

**Affiliations:** 1 Division of Fruits and Horticultural Technology, ICAR-Indian Agricultural Research Institute, New Delhi, Delhi, India; 2 ICAR Research Complex for NEH Region, Tripura Centre, Lembucherra, Tripura, India; 3 ICAR- Central Soil Salinity Research Institute, Karnal, Haryana, India; 4 ICAR-Central Horticultural Experiment Station, Vejalpur, Panchmahals, Gujarat, India; 5 ICAR-Central Arid Zone Research Institute, Regional Research Station, Pali Marwar, Rajasthan, India; 6 ASPEE College of Horticulture, Navsari Agricultural University, Navsari, Gujarat, India; 7 Department of Horticultural Sciences, Faculty of Agriculture and Natural Resources, Arak University, Arak, Iran; 8 Republic of Türkiye, Ministry of Agriculture and Forestry, General Directorate of Agricultural Research and Policies, Hatay Olive Research Institute Directorate, Hassa, Hatay, Türkiye; Universitat Jaume 1, SPAIN

## Abstract

Plants rely heavily on root-associated microflora to mitigate various environmental stresses such as drought and the use of arbuscular mycorrhizal fungi (AMF) as a biological instrument to enhance plant drought tolerance, through the formation of mutualistic associations, seems like a novel strategy for sustainable agriculture. Thus, a glasshouse study was undertaken with two citrus rootstocks (Troyer citrange and Cleopatra mandarin) under 20 days of differential moisture regimes (well-watered and water-stress condition), after 270 days of inoculation with two AMF strains (*Glomus intraradices* and mixed strains of *Gigaspora decipiens* + *Glomus mosseae*) alone or in combination with mycorrhizal helper bacteria (MHB), i.e., phosphate solubilising bacteria (PSB), *Azospirillum* or *Providencia*, with an objective of testing the hypothesis that AMF and MHB might influence ABA accumulation in citrus leaves, thereby altering physio-biochemical response to declining soil water status. The seedlings dually inoculated with AMF and MHB exhibited significantly higher relative water content of leaf as well as rhizospheric soil. *Glomus intraradices* in association with PSB or *Azospirillum* accumulated higher concentrations of osmolytes (phenols, proline and total soluble sugars) and antioxidant metabolites (glutathione and carotenoids) in leaves under WS condition. Significantly altered leaf tissue ABA accumulation was noticed in dual inoculated treatment of *Glomus intraradices* and *Azospirillum* for Troyer citrange (3.517 µg g-1 F.W.) and *G*. *intraradices* and PSB for Cleopatra mandarin seedlings (3.079 µg g-1 F.W.), under water-stress condition, as compared to other microbial inoculants including control. Thus, water deficit stress induced physiological and biochemical response pathways could be modified by AMF and MHB. Under these experimental conditions, Troyer citrange exhibited higher relative water content, photosynthetic activity, and stomatal conductance under drought conditions than Cleopatra mandarin seedlings, offering a potential microbial intervention strategy for mitigating osmotic stress in semi-arid environments.

## 1. Introduction

Plants in nature are continuously exposed to several biotic and abiotic stresses, water deprivation being one of the commonest. Water-stress is one of the most important environmental factors limiting growth and development of crop plants such as citrus, which has relatively few and short root hairs and are fairly dependent on arbuscular mycorrhizal fungi (AMF) that are most *Glomus* species [1]. Inoculation of AMF has been found to modify whole plant response to water deficit stress [2], including stomatal response to possible changes in drought-associated physiological traits and leaf ABA accumulation [3]. The mycorrhizal plants sometimes show stomatal conductance different from those of control plants, when only part of a root system dries and before leaf water relations are affected, suggesting an AMF effect on drought-associated physiological traits [4]. The abscisic acid (ABA) appears to be a primary candidate associated with the water deficit stress response [5]. It is an inhibitor of stomatal opening [6] and AMF can affect host balances of ABA [7]. Duan et al. [8] reported that mycorrhizal symbiosis did not alter stomatal sensitivity to ABA, but that the amount of ABA moving in xylem as a function of soil water content around root systems was lower in mycorrhizal plants than in similar-sized control plants in dry soil. Lower concentrations of ABA in mycorrhizal versus non-mycorrhizal roots at similar soil water contents have also been observed in water deficit stressed alfalfa [9]. Thus, it seems possible that mycorrhizal fungi may affect root metabolic responses to environmental stress and alter the production of chemical ‘stress’ signals in roots [3]. Thus, a better understanding of this complex interconnected signalling pathways and AMF-mediated mechanisms that regulate drought tolerance in plants will open new approaches to exploit AMF as a bioprotective tool against drought [10]. Mycorrhiza helper bacteria (MHB) such as *Bacillus*, *Pseudomonas*, *Azospirillum*, and *Providencia* are known to stimulate root colonization by AMF, promote pre-symbiotic mycelial growth and increase signalling communication between AMF and plant host, thereby improving root receptivity [11].

Keeping the above points, it is reasonable to hypothesize that AMF and MHB might in some way modify drought-associated physiological traits and thus influence stomatal behaviour, whether mycorrhizal symbiosis along with helper bacteria can actively modulate leaf tissue ABA accumulation and downstream drought adaptive features across different citrus genotypes remains to be fully clarified. Therefore, we hypothesized that AMF along with MHB enhanced soil-plant hydraulic conductance and plant water status during drought. To test these hypotheses, we measured the co-inoculation effects of AMF and MHB on morpho-physiological and biochemical traits in citrus rootstocks subjected to low moisture stress. Thus, our study aimed to induce drought tolerance in citrus rootstocks through microbial interventions.

## 2. Materials and methods

### 2.1. Experimental details

The experiment was conducted during three consecutive years in Indian Agricultural Research Institute (IARI), New Delhi, which belongs to Trans-Gangetic Plains of agro-climatic zones of India. Climate is categorized as semi-arid, subtropical, with hot, dry summer and cold winter and it falls in the Agro-eco-region-IV. May and June are the hottest months with the maximum temperature ranging from 41–44 ^°^C, and December and January are the coldest months, with the minimum temperature ranging between 3–7 °C. The mean annual rainfall is 710 mm of which as much as 75% is received during monsoon season (June to September). Some winter showers are also received during December-March. Frost occurs occasionally during month of December-January. The average relative humidity varies from 34.1 to 97.9% and average wind speed is 0.45 to 3.96 m/s. The study was performed in two citrus rootstocks, viz. Troyer citrange (*Citrus sinensis* × *Poncirus trifoliata*) and Cleopatra mandarin (*C*. *reshni*), collected from the Citrus Germplasm Block of the Division of Fruits and Horticultural Technology, IARI. Seeds of the two citrus rootstocks were surface sterilized by immersing in 70% alcohol for five minutes, followed by rinsing with sterile distilled water and then germinated on wet filter paper in Petri dishes at 28 °C. After 7 days these seedlings were transplanted in plastic containers (12 cm × 20 cm) containing mixture of soil: sand: FYM (2:2:1) under glasshouse conditions. The potting mixtures were sterilized by placing them in pre-heated oven at temperature of 82–93 °C for 30 minutes after covering tightly with aluminum foil, then let them cool completely inside the oven before transferring them into the plastic containers. Plastic containers were sterilized by washing them with soapy water and soaking them in 10% bleach solution for 10–30 minutes followed by rinsing thoroughly with clean water and air-drying completely in the sun. The potting mixture had EC (1:2) 6.35 mS m^-1^, pH (1:2) 7.92, HCO_3_^-^ 1.14 g kg^-1^ and Cl^-^ 5910.75 ppm. Two AMF strains, viz., *Glomus intraradices* and mixed strains (*Gigaspora decipiens* + *Glomus mosseae*) and three mycorrhizal helper bacterial strains, viz., PSB (*Bacillus subtilis* + *B*. *megaterium*), *Azospirillum* and *Providencia* were used in the experiment. *Glomus intraradices* was procured from the Division of Microbiology, K. R. C. College of Horticulture, Arabhavi, Karnataka, while mixed strains and all the bacterial strains were purchased from Division of Microbiology, IARI, New Delhi. The AMF propagules (approx. 20 g inocula per pot) either alone or in combination with helper bacteria were put at 5 cm below the seedlings at transplanting in plastic containers. The seedlings were maintained in glasshouse with day night temperatures ranging from 27 ± 1 °C. Day lengths were being extended up to 16 h with cool white fluorescent lights at 630 µmol m^-2^s^-1^. Seedlings were watered on alternate days with sterile tap water. Prior to shifting of plants, all greenhouse benches and floors were rigorously disinfected with sodium hypochlorite. Control and inoculated plants were arranged on separate benches spaced far apart inside the glasshouse. After two months, the seedlings along with plastic containers were transferred to open conditions at High-Density Orchard of Division of Fruits and Horticultural Technology, IARI, New Delhi. To successfully prevent cross-contamination between control plants and microbial inoculated plants grown in pots under open outdoor conditions, the control and inoculated plants were placed in separate rows of 30 cm height at 1.50-meter distance and the seedlings were irrigated at two-day intervals by pouring water gently and slowly directly onto the soil surface at the base of the plant. Water used for irrigation had EC (1:2) 288 µS m^-1^, pH (1:2) 7.48, HCO_3_^-^ 1.0 meq l^-1^ and Cl^-^ 110.76 ppm. The experiment was laid out in completely randomized design with 24 treatment combinations (two citrus rootstocks, two mycorrhizal cultures and three bacterial strains). The entire experiment was replicated across three independent chronological annual runs (2021–2022, 2022–2023, and 2023–2024) under identical glasshouse conditions to guarantee experimental reproducibility. Within each annual run, every treatment combination comprised two independent biological replications. Each replication consisted of six separate pots, with one uniform seedling grown per pot, where each individual potted seedling served as a distinct independent experimental unit (yielding 12 pots per treatment per year, and a pooled sample size of n = 6 true biological replicates per treatment combination across the three years). The treatment included T_1_ = Troyer citrange control plants, T_2_ = Troyer citrange inoculated with PSB, T_3_ = Troyer citrange inoculated with *Azospirillum* sp., T_4_ = Troyer citrange inoculated with *Providencia* sp., T_5_ = Troyer citrange inoculated with *G*. *intraradices*, T_6_ = Troyer citrange co-inoculated with *G*. *intraradices* and PSB, T_7_ = Troyer citrange co-inoculated with *G*. *intraradices* and *Azospirillum* sp., T_8_ = Troyer citrange co-inoculated with *G*. *intraradices* and *Providencia* sp., T_9_ = Troyer citrange inoculated with mixed strains (mixture of *Gigaspora decipiens*  + *G*. *mosseae*), T_10_ = Troyer citrange co-inoculated with mixed strains and PSB, T_11_ = Troyer citrange co-inoculated with mixed strains and *Azospirillum* sp., T_12_ = Troyer citrange co-inoculated with mixed strains and *Providencia* sp., T_13_ = Cleopatra mandarin control plants, T_14_ = Cleopatra mandarin inoculated with PSB, T_15_ = Cleopatra mandarin inoculated with *Azospirillum* sp., T_16_ = Cleopatra mandarin inoculated with *Providencia* sp., T_17_ = Cleopatra mandarin inoculated with *G*. *intraradices*, T_18_ = Cleopatra mandarin co-inoculated with *G*. *intraradices* and PSB, T_19_ = Cleopatra mandarin co-inoculated with *G*. *intraradices* and *Azospirillum* sp., T_20_ = Cleopatra mandarin co-inoculated with *G*. *intraradices* and *Providencia* sp., T_21_ = Cleopatra mandarin inoculated with mixed strains, T_22_ = Cleopatra mandarin co-inoculated with mixed strains and PSB, T_23_ = Cleopatra mandarin co-inoculated with mixed strains and *Azospirillum* sp., T_24_ = Cleopatra mandarin co-inoculated with mixed strains and *Providencia* sp. Each of the 24 treatments had two replications. The differential water treatment was started at 270 days after inoculation under glasshouse condition. The citrus seedlings of each replication of each treatment (six seedlings per replication per treatment) were divided into two equal sets and each set was subjected to either water-stress or well-watered condition, during which soil relative water content was measured using soil moisture meter fitted with 4.7 inches rods. Wet point was fixed at 90% and dry point at 8%. The soil relative water content (RWC) for WW plants was monitored at 80%. Duration of withholding irrigation was 20 days, when more than 50% treatment under water-stress condition, showed visible symptoms of temporary wilting. All plants were harvested at the end of the 20-day dry-down period for downstream physiological and biochemical profiling.

#### 2.1.1. Statement specifying permissions.

The study was done in the Indian Agricultural Research Institute (IARI). We acquired permission from the Indian Agricultural Research Institute (IARI) to conduct the study.

#### 2.1.2. Statement on experimental research and field studies on plants.

The cultivated or wild-growing plants sampled comply with relevant institutional, national, and international guidelines and domestic legislation of India.

### 2.2. Parameters recorded

The percentage of AM colonization in roots was analysed before water-stress imposition at 270 DAI and after 20 days of differential water treatment for water-stressed (WS) plants and well-watered (WW) plants by using the method as detailed by Phillips and Hayman [12]. Root samples were cut into pieces and fixed on formalin: acetic acid: alcohol (FAA) in the ratio of 5: 5: 90 for 24 hours. The roots were then cleaned by autoclaving with 10% KOH at 15 lbs pressure for 15 minutes. The alkalinity due to KOH was neutralised by adding 1.00% HCl for five minutes. Staining was done by immersing the roots in 0.05% trypan blue solution, prepared by adding 0.25 g of trypan blue powder in 500 ml lactoglycerol (438 ml lactic acid: 32 ml glycerine: 30 ml water) for 10 minutes. Excess stain was decanted and stained roots were stored in lactoglycerol. The root bits (one centimetre long) were arranged on slides using forceps. One drop of polyvinyl glycol was put on each root segment and thereafter the root segments were gently pressed between two glass slides for fixation. The presence of fungal hyphae, arbuscules, and vesicles was examined under a 10× compound microscope. For each distinct biological replication, 30 separate root segments (each 1 cm long) were scanned using a modified root-bit segment counting method to verify structural infection parameters. The total % root colonisation was calculated using the following formula:


$$\begin{array}{c} \text{Percent root colonization}=\frac{\text{Number of root bits positive for colonization}}{\text{Total number of root bits observed under microscope}}\times \text{100} \end{array}$$

Soil relative water content was recorded daily, starting from the beginning till the end of moisture stress period (20 days), using soil moisture meter fitted with 4.7-inch rods and then expressed as percentage. Four fully developed leaves were selected from each replication of each treatment for leaf area measurement (cm^2^) with the help of LiCor-3000 leaf area meter, just prior to the imposition of differential moisture regimes and after water treatment for water-stress and well-watered treatment. Relative water content (RWC) of the fifth fully expanded leaf from the shoot apex, just prior to the imposition of differential moisture regimes and after water treatment for water-stress and well-watered treatment was determined following the method suggested by Barrs and Weatherley [13].

Chlorophyll ‘a’, chlorophyll ‘b’, total chlorophyll and total carotenoids content in leaf were determined just prior to the imposition of differential moisture regimes and after water treatment for water-stress and well-watered treatment using the method suggested by Hiscox and Israelstam [14]. Photosynthetic and respiration rate and stomatal conductance were measured just prior to the imposition of differential moisture regimes and after water treatment for water-stress and well-watered treatment by using Infra-Red Gas Analyser PS System II (Li-Cor 6200) from 9:00 am to 11:00 am. The youngest fully emerged leaf (third from top) of each replication of each treatment was enclosed in a chamber, sealed to avoid gas exchange with the atmosphere and the chamber was placed in sunlight to measure photosynthetic rate (expressed in µmol m^-2^s^-1^) and stomatal conductance (expressed in mmol m^-2^s^-1^).

Leaf proline content was estimated as per the method of Bates et al. [15]. Total phenol content in leaf was estimated according to Malik, and Singh [16]. Total soluble sugar of leaf was estimated and compared with the prepared standard curve by the method as suggested by Buysse and Merckx [17]. The antioxidants metabolite like ascorbic acid was estimated as described by Mukherjee and Choudhuri [18]. The absorbance was recorded at 530 nm. The concentration of ascorbic acid was calculated from a standard curve plotted with known concentration of ascorbic acid in the range of 1–12 ug ml^-1^. Glutathione was assayed according to the method of Grifith [19] and Smith [20]. All spectrophotometric analyses were conducted using an UV-visible Spectrophotometer-Ultrospec 2000 (Pharmacia Biotech).

Abscisic acid (ABA) content in leaf tissue was determined just prior to the imposition of differential moisture regimes and at the conclusion of the experimental dry-down window for both water-stressed (WS) and well-watered (WW) conditions using the method suggested by Ali *et al*. [21]. Extraction was performed using 80% methanol at 4 °C in absolute dark to prevent light-induced isomerization of ABA. The crude extract was evaporated down to the aqueous phase. Subsequent acidification (pH 2.5–3.0) allowed partitioning into an organic phase of ethyl acetate. To eliminate residual chlorophyll and lipids, the extract was passed through a C18 solid-phase extraction (SPE) cartridge, pre-conditioned with methanol and acidified water.

Chromatographic separation was achieved via an HPLC-UV system equipped with a reversed-phase C18 column (250 mm × 4.6 mm, 5 µm particle size). The mobile phase consisted of an isocratic mixture of methanol and 0.1% aqueous acetic acid (60:40, v/v) pumped at a constant flow rate of 1.0 mL/min. Standard monitoring and spectral detection were performed at a fixed UV wavelength of 254 nm. Primary standard reference materials (≥98% purity, Sigma-Aldrich) were dissolved in chromatographically pure methanol to prepare a concentrated stock solution. Working standards were prepared via serial dilution to construct a five-point external calibration curve. The baseline method recovery rate (R) was evaluated at 75.8%. The final absolute concentration of ABA in the leaf tissue was calculated using the following equation:


$$\begin{array}{c} \text{ABA content in leaf }(\mu \text{g g}-\text{1}\text{ F}.\text{W}.)=\frac{Xvial\times V}{\text{W }\times R\;}\times \text{100} \end{array}$$

where Xvial represents the concentration in the HPLC vial (μg/mL), V is the final volume of the reconstituted extract (mL), W is the fresh weight of the processed leaf tissue (g), and R is the method recovery rate factor expressed as a fraction (0.758).

### 2.3. Statistical data analysis

All data collected over the three independent experimental runs were compiled and subjected to rigorous statistical screening. Prior to pooling the data across years, the homogeneity of variance was verified using Levene’s test (*p* > 0.05). Because there were no significant interactions between the experimental treatments and the trial years, the annual datasets were pooled into a single balanced dataset framework. To evaluate the main effects and multi-way interactions, a multi-way full factorial analysis of variance (ANOVA) was executed based on a General Linear Model (GLM) evaluating Citrus Rootstock, Microbial Treatment, and Moisture Regime as fixed factors. Each year represents an independent experimental run treated as a random block effect. Within the statistical model, each individual potted seedling was defined as an independent experimental unit, and the values from the distinct units per replication were compiled across the three years to establish a true biological replication size of n = 6.

For the rhizospheric soil relative water content (RWC) data collected sequentially over a 20-day dry-down window, a Repeated-Measures ANOVA was implemented to track temporal fluctuations and evaluate time × treatment interaction cascades. Proportional colonization rates were normalized using Arc Sin √% transformations prior to statistical modeling, where omnibus F-tests indicated significant variance *p* < 0.05, mean separation was done using Fisher’s protected Least Significant Difference (LSD) tests at α = 0.05. Bivariate relationship modeling was conducted via Pearson correlation analysis (2-tailed significance testing), with exact coefficient magnitudes (r) coupled to true evaluated sample dimensions (n = 24 treatment mean nodes). All analyses were done using SPSS software version 16.0.

## 3. Results

### 3.1. Root colonisation

The multi-way ANOVA demonstrated a highly significant interaction between rootstocks, AMF strains, and MHB treatments for root colonization percentage (*p* < 0.05). The water-stress treatment notably decreased root colonization. No mycorrhizal colonization was observed in the roots of non-AM seedlings under water stress condition. Negligible background mycorrhizal colonization or staining artifacts (6.67% to 16.66%) were observed in the non-AM inoculated control seedlings, which was significantly lower than all intentional AMF-inoculated treatments before imposing differential moisture stress and also under well-watered condition (F_23, 264_ = 42.1, *p* < 0.001). However, dual inoculated treatment had significantly highest root colonization, as compared to single inoculation, particularly *G*. *intraradices* + PSB, under moisture stress condition (Fig 1). Troyer citrange and Cleopatra mandarin recorded significantly highest root colonisation just before imposing differential water treatment (83.34 and 80.00%, respectively) and after 20 days of differential water treatment for both moisture stressed (66.67 and 62.50%, respectively) and well-watered plants (87.50 and 83.33%, respectively), when dually inoculated with *G*. *intraradices* and phosphate solubilising bacteria (PSB) (T_6_ and T_18_, respectively), which were at par with seedlings dually inoculated with *G*. *intraradices* and *Azospirillum* sp. (T_7_) (76.66%) before stress, *G*. *intraradices* and *Azospirillum* sp. (T_7_) (62.50%) and *G*. *intraradices* and *Providencia* sp. (T_8_) (58.33%) under water-stress condition and *G*. *intraradices* and *Azospirillum* sp. (T_7_) (83.33%) and *G*. *intraradices* and *Providencia* sp. (T_8_) (79.16%) under well-watered condition for Troyer citrange and seedlings dually inoculated with *G*. *intraradices* and *Azospirillum* sp. (T_19_) (79.16%) and *G*. *intraradices* and *Providencia* sp. (T_20_) (75.00%) under well-watered condition for Cleopatra mandarin (Table 1).

**Table 1:** Effect of microbial inoculants on root colonization (%) of citrus under differential soil moisture regimes*.

Treatment	BS	WS	WW
T_1_	6.67 (14.97)^g^	0.00 (0.83)^e^	8.33 (16.77)^g^
T_2_	13.34 (20.77)^f^	0.00 (0.83)^e^	16.66 (23.39)^f^
T_3_	13.33 (21.41)^f^	0.00 (0.83)^e^	16.67 (24.10)^f^
T_4_	10.00 (18.19)^fg^	0.00 (0.83)^e^	12.50 (20.44)^fg^
T_5_	66.67 (54.74)^bc^	54.17 (47.47)^b^	75.00 (60.00)^bc^
T_6_	83.34 (66.01)^a^	66.67 (54.74)^a^	87.50 (69.56)^a^
T_7_	76.66 (61.17)^ab^	62.50 (52.27)^a^	83.33 (65.90)^ab^
T_8_	70.00 (56.82)^b^	58.33 (49.79)^ab^	79.16 (62.95)^b^
T_9_	46.67 (43.09)^de^	25.00 (30.00)^d^	58.33 (49.79)^de^
T_10_	60.00 (50.82)^cd^	37.50 (37.73)^c^	70.84 (57.37)^cd^
T_11_	53.33 (46.91)^d^	33.33 (35.26)^cd^	66.67 (54.74)^d^
T_12_	50.00 (45.00)^de^	29.16 (32.63)^cd^	62.50 (52.27)^de^
T_13_	6.67 (14.97)^g^	0.00 (0.83)^e^	8.33 (16.77)^g^
T_14_	16.66 (23.99)^f^	0.00 (0.83)^e^	20.84 (27.05)^f^
T_15_	13.34 (20.77)^f^	0.00 (0.83)^e^	16.66 (23.39)^f^
T_16_	13.33 (21.41)^f^	0.00 (0.83)^e^	16.67 (24.10)^f^
T_17_	50.00 (45.00)^de^	33.33 (35.26)^cd^	70.84 (57.37)^cd^
T_18_	80.00 (63.44)^a^	62.50 (52.27)^a^	83.33 (65.90)^ab^
T_19_	70.00 (56.82)^b^	37.50 (37.73)^c^	79.16 (62.95)^b^
T_20_	66.67 (54.74)^bc^	41.67 (40.21)^bc^	75.00 (60.00)^bc^
T_21_	46.67 (43.09)^de^	25.00 (30.00)^d^	58.33 (49.79)^de^
T_22_	60.00 (50.82)^cd^	25.00 (30.00)^d^	70.84 (57.37)^cd^
T_23_	53.33 (46.91)^d^	29.16 (32.63)^cd^	70.84 (57.37)^cd^
T_24_	50.00 (45.00)^de^	29.16 (32.63)^cd^	62.50 (52.27)^de^

Note: *BS = Before stress, WS = Water deficit stress, WW = Well-watered; Grouping letters (a–g) indicate significant differences within each column based on Fisher’s protected LSD test (n = 6, p < 0.05, SPSS 16.0). Values in parentheses represent Arc Sin √% transformed values.

Treatment details: T_1_ = Troyer citrange control plants, T_2_ = Troyer citrange inoculated with PSB, T_3_ = Troyer citrange inoculated with *Azospirillum* sp., T_4_ = Troyer citrange inoculated with *Providencia* sp., T_5_ = Troyer citrange inoculated with *G*. *intraradices*, T_6_ = Troyer citrange co-inoculated with *G*. *intraradices* and PSB, T_7_ = Troyer citrange co-inoculated with *G*. *intraradices* and *Azospirillum* sp., T_8_ = Troyer citrange co-inoculated with *G*. *intraradices* and *Providencia* sp., T_9_ = Troyer citrange inoculated with mixed strains (mixture of *Gigaspora decipiens*  + *G*. *mosseae*), T_10_ = Troyer citrange co-inoculated with mixed strains and PSB, T_11_ = Troyer citrange co-inoculated with mixed strains and *Azospirillum* sp., T_12_ = Troyer citrange co-inoculated with mixed strains and *Providencia* sp., T_13_ = Cleopatra mandarin control plants, T_14_ = Cleopatra mandarin inoculated with PSB, T_15_ = Cleopatra mandarin inoculated with *Azospirillum* sp., T_16_ = Cleopatra mandarin inoculated with *Providencia* sp., T_17_ = Cleopatra mandarin inoculated with *G*. *intraradices*, T_18_ = Cleopatra mandarin co-inoculated with *G*. *intraradices* and PSB, T_19_ = Cleopatra mandarin co-inoculated with *G*. *intraradices* and *Azospirillum* sp., T_20_ = Cleopatra mandarin co-inoculated with *G*. *intraradices* and *Providencia* sp., T_21_ = Cleopatra mandarin inoculated with mixed strains, T_22_ = Cleopatra mandarin co-inoculated with mixed strains and PSB, T_23_ = Cleopatra mandarin co-inoculated with mixed strains and *Azospirillum* sp., T_24_ = Cleopatra mandarin co-inoculated with mixed strains and *Providencia* sp.

**Fig 1:**
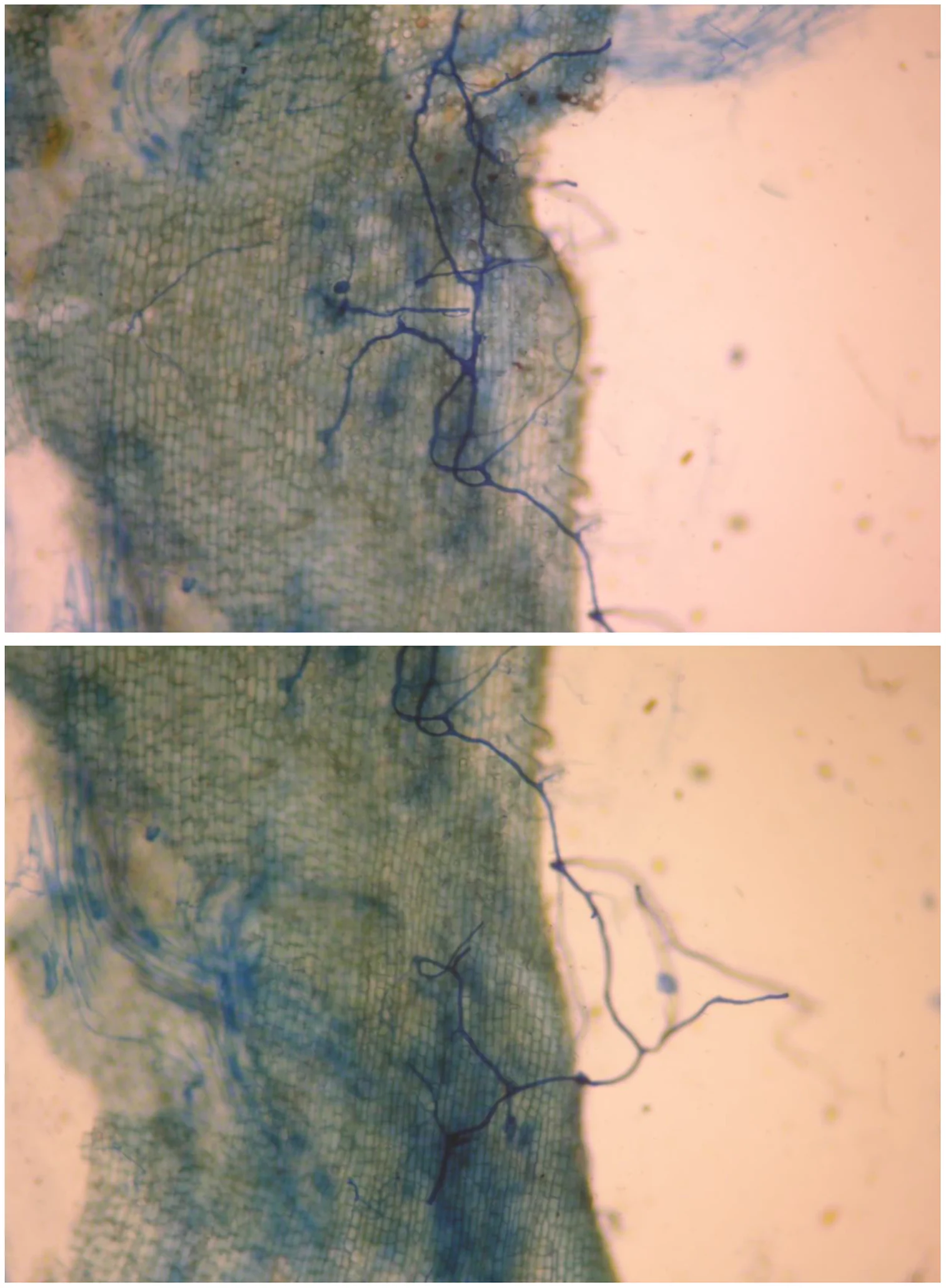
Root colonization of Troyer citrange and Cleopatra mandarin inoculated with *Glomus intraradices* and PSB under water deficit stress condition.

### 3.2. Soil relative water content

Soil relative water content (RWC) in the rhizosphere declined progressively during the 20-day moisture stress period in both Troyer citrange and Cleopatra mandarin (Fig 2). Repeated-measures analysis revealed significant effects of treatment, time, and the treatment × time interaction on soil RWC (*p* < 0.05), indicating that microbial inoculation significantly influenced the pattern of soil moisture depletion during drought.

**Fig 2:**
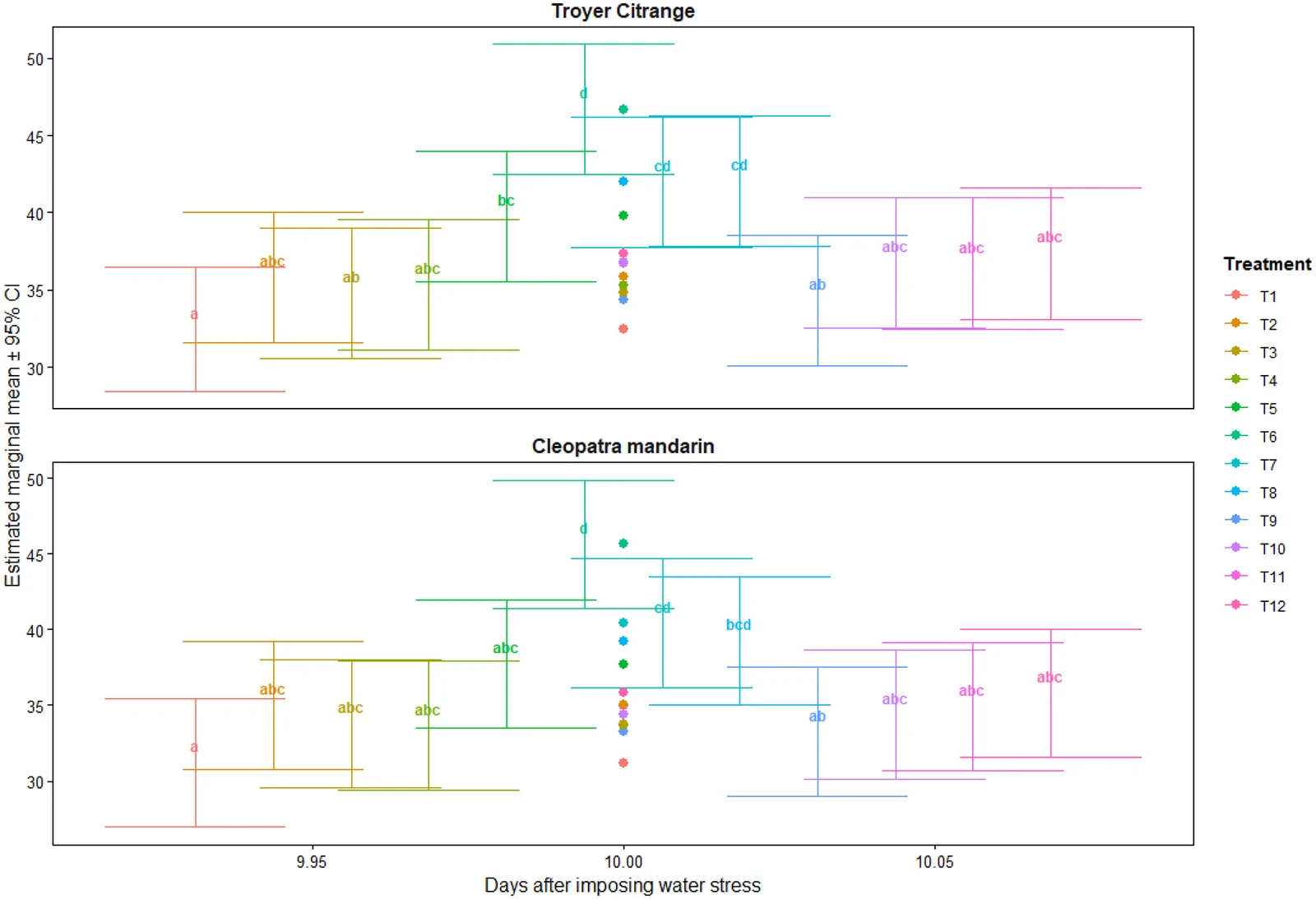
Soil relative water content in the rhizospheres of citrus rootstocks as influenced by AMF and MHB during the period of moisture stress. *{Note: Letters within a column denote statistically significant differences according to rigorous repeated-measures mixed-model analysis in SPSS 16.0 (n = 6, p < 0.05). For treatment details, please see* Table 1*}.*

At the initiation of moisture stress, soil RWC did not differ significantly among treatments. Differences became evident after the onset of water deficit and increased with the duration of stress. Across the experimental period, seedlings inoculated with *G*. *intraradices* + PSB consistently maintained higher soil RWC than the other microbial treatments and the uninoculated control in both citrus rootstocks.

In Troyer citrange, soil RWC in the *G*. *intraradices* + PSB treatment (T_6_) declined gradually from 85.50% on day 2 to 10.50% on day 20, whereas the uninoculated control (T_1_) declined from 80.50% to 8.00% over the same period. Similarly, in Cleopatra mandarin, soil RWC decreased from 82.00 to 9.50% in the *G*. *intraradices* + PSB treatment (T_18_), compared with 82.00 to 8.00% in the uninoculated control. The slower decline in soil RWC under dual inoculation was maintained throughout the drought period in both rootstocks.

Treatments combining *G*. *intraradices* with *Providencia* sp. or *Azospirillum* sp. also maintained higher soil RWC than the uninoculated control, although their performance was consistently lower than that of the *G*. *intraradices* + PSB treatment. Single inoculation with *G*. *intraradices* produced intermediate responses, whereas the uninoculated seedlings exhibited the most rapid reduction in soil RWC throughout the moisture stress period.

At the end of the experiment, soil RWC was highest in seedlings receiving *G. intraradices* + PSB, reaching 10.50% in Troyer citrange (T_6_) and 9.50% in Cleopatra mandarin (T_18_), while both uninoculated controls retained only 8.00% soil RWC. These results demonstrate that dual inoculation with *G*. *intraradices* and PSB delayed rhizospheric soil moisture depletion throughout the drought period compared with the other treatments.

### 3.3. Leaf relative water content

Water-stress reduces the relative water content of leaf. However, AM plants maintained higher leaf water potential as compared to control plants, under WS condition. Fungi *G*. *intraradices* in association with PSB significantly enhanced the relative water content in Troyer citrange (T_6_) (94.25, 87.23 and 95.62%) and Cleopatra mandarin (T_18_) (93.31, 86.04 and 95.64%) at BS and under WS and WW conditions, respectively, which was at par with Troyer seedlings inoculated with *G*. *intraradices* + *Providencia* sp. (T_8_) (86.52%) under WS condition and the dual inoculated treatment was found significantly superior over uninoculated control for Troyer citrange (T_1_) (73.62, 59.55 and 76.56%) and Cleopatra mandarin (T_13_) (72.95, 52.78 and 77.09%) at BS and under WS and WW conditions, respectively (Table 2).

**Table 2:** Effect of microbial inoculants on leaf relative water content (%) and leaf area (cm^2^) of citrus seedlings under differential soil moisture regimes*.

Treatment	Leaf relative water content (%)			Leaf area (cm^2^)		
	BS	WS	WW	BS	WS	WW
T_1_	73.62^g^	59.55^g^	76.56^g^	6.35^h^	6.36^g^	9.38^f^
T_2_	74.34^g^	58.57^g^	76.33^g^	6.03^h^	6.04^g^	9.65^f^
T_3_	80.47^ef^	74.46^e^	80.63^ef^	6.01^h^	6.01^g^	9.46^f^
T_4_	78.61^f^	69.39^f^	80.47^f^	7.18^gh^	7.19^g^	10.37^ef^
T_5_	83.77^de^	77.53^de^	85.36^de^	9.03^ef^	9.24^ef^	16.19^cd^
T_6_	94.25^a^	87.23 ^a^	95.62^a^	10.52^ce^	11.63^cd^	17.59^bc^
T_7_	88.86^bc^	76.46^de^	90.65^bc^	10.41^de^	12.89^bc^	18.90^b^
T_8_	88.19^bc^	86.52^a^	95.09^a^	10.85^cd^	11.44^cd^	15.70^d^
T_9_	79.47^f^	70.42^f^	81.54^f^	7.18^gh^	7.26^fg^	10.71^e^
T_10_	85.65^cd^	80.47^cd^	88.56^cd^	10.38^de^	10.62^d^	11.21^e^
T_11_	85.36^cd^	76.33^e^	87.08^d^	9.60^e^	10.92^cd^	14.58^d^
T_12_	83.77^de^	79.5^cd^	84.61^de^	8.58^f^	9.02^ef^	17.12^c^
T_13_	72.95^g^	52.78^h^	77.09^g^	7.57^g^	7.59^fg^	15.24^d^
T_14_	82.97^de^	51.66^h^	83.33^de^	11.60^bc^	11.62^cd^	16.79^cd^
T_15_	82.84^de^	69.91^f^	84.42^de^	9.93^de^	9.94^de^	14.24^d^
T_16_	78.76^f^	53.39^h^	80.91^f^	12.34^b^	12.41^bc^	17.36^bc^
T_17_	85.28^cd^	79.58^cd^	90.19^bc^	13.36^b^	13.68^b^	18.22^b^
T_18_	93.31^a^	86.04^ab^	95.64^a^	16.34^a^	17.47^a^	25.23^a^
T_19_	88.19^bc^	77.13^de^	90.84^b^	13.09^b^	15.85^ab^	23.59a
T_20_	86.34^cd^	82.48^bc^	92.08^b^	14.26^ab^	15.42^ab^	23.22^a^
T_21_	80.06^ef^	68.58^f^	84.61^de^	11.23^ce^	11.85 cd	17.81^bc^
T_22_	87.87^bc^	78.42^cd^	88.86^cd^	12.58^b^	13.64^b^	20.65^b^
T_23_	84.16^cd^	70.29^f^	86.82^d^	13.59^b^	15.39^ab^	21.30^ab^
T_24_	84.81^cd^	75.00^e^	86.04^d^	11.01^cd^	12.57^bc^	20.63^b^

Note: *BS = Before stress, WS = Water deficit stress, WW = Well-watered; Grouping letters (a–h) indicate significant treatment variations within columns via Fisher’s protected LSD test (n = 6, p < 0.05, SPSS 16.0). For treatment details, please see Table 1.

### 3.4. Leaf area

Leaf area was significantly improved by combined application of AM fungi and helper bacteria in both the citrus rootstocks, regardless of differential moisture treatment, although it was higher in Cleopatra mandarin compared to Troyer citrange. Co-inoculation of *G*. *intraradices* and PSB resulted in leaf production of maximum area in Cleopatra mandarin (T_18_) at BS and under water stress and well-watered condition (16.34, 17.47 and 25.23 cm^2^, respectively), which was statistically comparable to combined application of *G*. *intraradices* and *Providencia* sp. in Cleopatra mandarin (T_20_) at BS and under water stress and well-watered condition (14.26, 15.42 and 23.22 cm^2^, respectively), co-inoculation of *G*. *intraradices* and *Azospirillum* sp. in cleopatra mandarin under water stress and well-watered condition (15.85 and 23.59 cm^2^, respectively) and co-inoculation of mixed strains and *Azospirillum* sp. in Cleopatra mandarin under water stress and well-watered condition (15.39 and 21.30 cm^2^, respectively) (Table 2).

### 3.5. Chlorophyll *a*

*Glomus intraradices* + PSB inoculation (T_6_) significantly showed highest chlorophyll *a* content in Troyer citrange (2.52, 2.27 and 2.88 mg g^-1^) over other microbial culture and uninoculated control (T_1_) (1.57, 1.22 and 1.56 mg g^-1^) at BS and WS and WW conditions, respectively. In case of Cleopatra mandarin also, *G*. *intraradices* + PSB treatment (T_18_) recorded significant level of chlorophyll *a* (1.95, 1.96 and 2.64 mg g^-1^) over other microbial inoculants and control treatment (T_13_) (1.24, 1.14 and 1.64 mg g^-1^) at BS and under WS and WW conditions, respectively, which was at par with *Azospirillum* treatment (T_15_) (1.95 mg g^-1^) at BS (Table 3).

**Table 3:** Effect of microbial inoculants on leaf chlorophyll content (mg g^-1^ F.W.) in citrus rootstocks under differential soil moisture regimes*.

Treatment	Chlorophyll *a*			Chlorophyll *b*			Total Chlorophyll		
	BS	WS	WW	BS	WS	WW	BS	WS	WW
T_1_	1.57^ef^	1.22^gh^	1.56^ef^	0.29^f^	0.26^d^	0.36^e^	1.86^gh^	1.48^g^	1.92^fg^
T_2_	1.74^de^	1.65^d^	1.93^d^	0.37^df^	0.27^d^	0.67^ab^	2.11^ef^	1.93^d^	2.60^cd^
T_3_	1.57^ef^	1.44^e^	1.87^d^	0.43^ce^	0.31^cd^	0.23^f^	2.00^fh^	1.76^e^	2.11^ef^
T_4_	1.55^f^	1.28^fh^	1.57^ef^	0.40^df^	0.42^ab^	0.45^d^	1.96^fh^	1.70^ef^	2.02^f^
T_5_	2.06^bc^	1.91^bc^	2.09^c^	0.36^ef^	0.25^d^	0.42^de^	2.42^cd^	2.16^c^	2.51^d^
T_6_	2.52^a^	2.27^a^	2.88^a^	0.38^df^	0.37^bc^	0.59^bc^	2.91^a^	2.64^a^	3.46^a^
T_7_	2.20^b^	2.15^ab^	2.67^b^	0.39^df^	0.13^e^	0.57^c^	2.59^bc^	2.27^bc^	3.24^ab^
T_8_	2.18^bc^	1.89^c^	2.23^c^	0.38^df^	0.44^a^	0.45^d^	2.5^6c^	2.33^b^	2.67^cd^
T_9_	1.85^cd^	1.70^cd^	1.94^d^	0.82^a^	0.32^cd^	0.41^de^	2.67^b^	2.02^cd^	2.34^de^
T_10_	2.16^bc^	2.02^b^	2.16^c^	0.38^df^	0.29^d^	0.68^a^	2.54^c^	2.32^b^	2.84^c^
T_11_	2.01^bc^	1.94^bc^	2.03^cd^	0.38^df^	0.26^d^	0.39^de^	2.39^cd^	2.20^bc^	2.42^de^
T_12_	1.98^c^	1.88^c^	2.23^c^	0.34^ef^	0.22^d^	0.73^a^	2.33^d^	2.10^c^	2.95^bc^
T_13_	1.24^g^	1.14^h^	1.64^e^	0.29^f^	0.23^d^	0.24^f^	1.53^i^	1.37^g^	1.88^fg^
T_14_	1.18^g^	1.17^h^	1.66e	0.35^ef^	0.20^de^	0.24^f^	1.53^i^	1.37^g^	1.89^fg^
T_15_	1.95^c^	1.52^de^	1.64^e^	0.42^cf^	0.23^d^	0.43^de^	2.37^cd^	1.75^e^	2.07^f^
T_16_	1.59^ef^	1.31^fg^	1.43^f^	0.39^df^	0.36^bc^	0.59^bc^	1.98^fh^	1.67^f^	2.02^f^
T_17_	1.57^ef^	1.57^d^	1.96^d^	0.38^df^	0.33^c^	0.48^d^	1.94^fh^	1.90^d^	2.43^de^
T_18_	1.95^c^	1.96^bc^	2.64^b^	0.41^cf^	0.46^a^	0.48^d^	2.36^cd^	2.42^b^	3.12^b^
T_19_	1.65^de^	1.67^d^	2.35^c^	0.35^ef^	0.33^c^	0.38^e^	2.00^fh^	2.01^cd^	2.73^cd^
T_20_	1.52^f^	1.46^e^	2.12^c^	0.35^ef^	0.39^b^	0.32^e^	1.87^gh^	1.85^de^	2.44^de^
T_21_	1.69^de^	1.53^de^	1.86^d^	0.39^df^	0.38^bc^	0.35^e^	2.03^fg^	1.91^d^	2.21^e^
T_22_	1.57^ef^	1.51^de^	2.04^cd^	0.44^bc^	0.37^bc^	0.34^e^	2.01^fg^	1.88^de^	2.38^de^
T_23_	1.51^f^	1.56^d^	2.13^c^	0.36^ef^	0.36^bc^	0.28^f^	1.87^gh^	1.93^d^	2.42^de^
24	1.71^de^	1.68^cd^	2.05^cd^	0.40^df^	0.30^cd^	0.40^de^	2.11^ef^	1.98^d^	2.45^de^

Note: *BS = Before stress, WS = Water deficit stress, WW = Well-watered; Grouping letters (a–h) within a column denote statistically significant differences according to Fisher’s protected LSD test (n = 6, p < 0.05, SPSS 16.0). For treatment details, please see Table 1.

### 3.6. Chlorophyll *b*

At just before stress, Troyer seedlings inoculated with mixed strains (T_9_) recorded highest chlorophyll *b* content (0.82 mg g^-1^), as compared to control (T_1_) (0.29 mg g^-1^). Cleopatra seedlings recorded highest chlorophyll *b* content, when inoculated with mixed strains + PSB (T_22_) (0.44 mg g^-1^), *Azospirillum* sp. (T_15_) (0.42 mg g^-1^), *G*. *intraradices* + PSB (T_18_) (0.41 mg g^-1^), mixed strains + *Providencia* sp. (T_24_) (0.40 mg g^-1^), mixed strains (T_21_) (0.39 mg g^-1^), *Providencia* (T_16_) (0.39 mg g^-1^) and *G*. *intraradices* (T_17_) (0.38 mg g^-1^), as compared to uninoculated control (T_13_) (0.29 mg g^-1^).

Under WS condition, highest chlorophyll *b* content was found in seedlings inoculated with *G*. *intraradices* + *Providencia* sp. (T_8_), *G*. *intraradices* + PSB (T_6_) and *Providencia* culture (T_4_) (0.44, 0.37 and 0.42 mg g^-1^, respectively) over uninoculated control (T_1_) (0.26 mg g^-1^) for Troyer Citrange and seedlings dually inoculated with *G*. *intraradices* and PSB (T_18_) and *G*. *intraradices* and *Providencia* sp. (T_19_) (0.46 and 0.39 mg g^-1^, respectively) over uninoculated control (T_13_) (0.23 mg g^-1^) for Cleopatra mandarin. Under WW condition, Troyer seedlings inoculated with mixed strains + *Providencia* sp. (T_12_), mixed strains + PSB (T_10_) and single inoculation of PSB (T_2_) recorded highest chlorophyll *b* content (0.73, 0.68 and 0.67 mg g^-1^, respectively), as compared to control (T_1_) (0.36 mg g^-1^), while Cleopatra mandarin inoculated with Providencia culture alone (T_16_) showed highest chlorophyll *b* content (0.59 mg g^-1^), as compared to other microbial culture and uninoculated control (T_13_) (0.24 mg g^-1^) (Table 3).

### 3.7. Total chlorophyll

Total chlorophyll values collapsed under progressive water deficit stress. However, dually inoculated seedlings maintained significantly higher pigment concentrations. Before water-stress, Troyer and Cleopatra seedlings dually inoculated with *G*. *intraradices* and PSB (T_6_ and T_18_, respectively) exhibited highest total chlorophyll content (2.91 and 2.36 mg g^-1^, respectively), which was at par with Cleopatra seedlings inoculated with *Azospirillum* sp. (T_15_) (2.37 mg g^-1^).

After differential watering, *G*. *intraradices* + PSB treatment significantly recorded highest total chlorophyll, as compared to other treatment, for both Troyer Citrange (T_6_) (2.64 and 3.46 mg g^-1^) and Cleopatra mandarin (T_18_) (2.42 and 3.12 mg g^-1^) under WS and WW conditions, respectively. In case of Troyer citrange, the lowest content of total chlorophyll was found in uninoculated control seedlings (T_1_) (1.86, 1.48 and 1.92 mg g^-1^) at BS and under WS and WW conditions, respectively, which was at par with *Providencia* treatment (T_4_) (1.96 and 2.02 mg g^-1^) at BS and under WW condition, respectively. In case of Cleopatra mandarin, the lowest total chlorophyll content was observed in uninoculated control (T_13_) (1.53, 1.37 and 1.88 mg g^-1^) and PSB treatment (T_14_) (1.53, 1.37 and 1.89 mg g^-1^) at BS and under WS and WW conditions, respectively (Table 3).

### 3.8. Photosynthetic rate

Under 20-day stress period, photosynthetic rate declined, as compared to well-watered plants. Photosynthetic rate was significantly highest in *G*. *intraradices* + PSB and *G*. *intraradices* + *Azospirillum* sp. inoculated Troyer seedlings (T_6_ and T_7_, respectively) at just before differential water treatment (9.02 and 8.93 µmol m^-2^ s^-1^, respectively) over control (T_1_) (2.44 µmol m^-2^ s^-1^) and after 20 days for well-watered seedlings (8.82 and 8.86 µmol m^-2^ s^-1^, respectively), as compared to control (2.40 µmol m^-2^ s^-1^). In case of Cleopatra mandarin, significantly highest photosynthetic rate was found in *G*. *intraradices* + PSB (T_18_) inoculated seedlings (7.67 and 7.14 µmol m^-2^ s^-1^) over uninoculated control seedlings (T_13_) (3.98 and 3.88 µmol m^-2^ s^-1^) at before stress and after water treatment under well-watered condition, respectively.

Under water-stress condition, photosynthetic rate was significantly high in *G*. *intraradices* + PSB inoculated Troyer citrange and Cleopatra mandarin seedlings (T_6_ and T_18_, respectively) (7.57 and 6.45 µmol m^-2^ s^-1^, respectively), as compared to other microbial culture and also control Troyer and Cleopatra seedlings (T_1_ and T_13_, respectively) (1.27 and 1.23 µmol m^-2^ s^-1^, respectively) (Table 4).

**Table 4:** Effect of microbial inoculants on gas exchange features of citrus rootstocks under differential soil moisture regimes*.

Treatment	Net photosynthesis (µmol m^-2^s^-1^)			Leaf respiration (µmol m^-2^s^-1^)			Stomatal conductance (mmol m^-2^ s^-1^)		
	BS	WS	WW	BS	WS	WW	BS	WS	WW
T_1_	2.44^i^	1.27^h^	2.40^i^	8.29^a^	12.14^b^	8.75^a^	0.128^f^	0.084^e^	0.118^f^
T_2_	3.63^fg^	1.34^gh^	3.20^fg^	8.09^ab^	11.78^bc^	8.40^ab^	0.221^bc^	0.168^bc^	0.214^bc^
T_3_	3.46^gh^	2.01^f^	3.18^g^	7.75^b^	11.29^c^	7.88^b^	0.215^c^	0.172^b^	0.206^c^
T_4_	4.54^e^	2.13^ef^	4.37^e^	7.23^c^	10.48^d^	7.67^bc^	0.219^bc^	0.158^c^	0.215^bc^
T_5_	6.93^c^	5.58^b^	6.80^c^	5.50^ef^	8.74^f^	5.86^de^	0.230^b^	0.182^b^	0.225^b^
T_6_	9.02^a^	7.57^a^	8.82^a^	3.81^h^	7.52^g^	3.98^g^	0.269^a^	0.201^a^	0.267^a^
T_7_	8.93^a^	4.64^c^	8.86^a^	4.28^gh^	7.95^g^	4.61^f^	0.240^b^	0.193^a^	0.237^b^
T_8_	4.16^f^	3.84^d^	4.07^f^	4.81^fg^	8.08^g^	4.95^ef^	0.213^c^	0.178^b^	0.209^c^
T_9_	4.91^e^	2.33^e^	4.74^e^	7.96^ab^	10.04^de^	7.54^c^	0.225^b^	0.161^c^	0.223^b^
T_10_	5.10^de^	4.83^c^	5.00^d^	6.00^df^	9.79^e^	6.52^d^	0.239^b^	0.194^a^	0.236^b^
T_11_	4.85^e^	3.34^d^	4.40^e^	5.60^ef^	9.52^e^	5.95^de^	0.180^d^	0.112^d^	0.175^d^
T_12_	5.00^de^	3.93^d^	4.93^d^	6.15^de^	9.13^ef^	6.53^d^	0.234^b^	0.136^cd^	0.232^b^
T_13_	3.98^fh^	1.23^h^	3.88^gh^	7.99^ab^	13.11^a^	8.58^ab^	0.031^h^	0.006^g^	0.025^h^
T_14_	5.63^d^	2.35^e^	5.49^d^	7.03^c^	12.69^ab^	6.79^cd^	0.094^g^	0.014^fg^	0.074^g^
T_15_	2.59^i^	1.28^h^	2.52^i^	7.08^c^	12.31^b^	7.48^c^	0.094^g^	0.008^g^	0.062^g^
T_16_	5.75^d^	4.41^c^	5.65^d^	6.52^cd^	11.46^bc^	6.90^cd^	0.042^h^	0.008^g^	0.036^h^
T_17_	5.77^d^	5.54^b^	5.63^d^	5.49^ef^	9.18^ef^	5.93^de^	0.191^cd^	0.085^e^	0.174^d^
T_18_	7.67^b^	6.45^b^	7.14^b^	3.82^h^	7.83^g^	3.98^g^	0.243^b^	0.135^cd^	0.257^ab^
T_19_	6.72^c^	5.70^b^	6.24^c^	4.40^gh^	8.53^f^	4.60^f^	0.223^b^	0.122^d^	0.220^bc^
T_20_	3.90^gh^	2.02^f^	3.81^h^	4.96^fg^	8.87^f^	5.51^e^	0.094^g^	0.037^f^	0.084^g^
T_21_	2.96^hi^	2.62^e^	2.92^h^	7.54^bc^	10.73^cd^	8.12^ab^	0.087^g^	0.056^ef^	0.077^g^
T_22_	2.58^i^	2.36^e^	2.52^i^	6.73^cd^	10.40^d^	7.05^c^	0.154^e^	0.096^de^	0.121^ef^
T_23_	2.87^hi^	2.66^e^	2.81^h^	6.58^cd^	10.22^de^	7.04^c^	0.106^fg^	0.061^ef^	0.097^fg^
T_24_	3.06^h^	2.26^e^	3.03^h^	6.31^de^	10.34^d^	6.53^d^	0.108^fg^	0.076^e^	0.137^e^

Note: *BS = Before stress, WS = Water deficit stress, WW = Well-watered; Shared grouping values (**a–i**) present statistically homogeneous results column-wise via LSD thresholds (n = 6, α = 0.05, SPSS 16.0). For treatment details, please see Table 1.

### 3.9. Respiration rate

Leaf respiration rates at BS were maximum in control (T_1_) and PSB inoculated seedlings (T_2_) of Troyer citrange (8.29 and 8.09 µmol m^-2^ s^-1^, respectively), which was statistically comparable to Cleopatra mandarin control (T_13_) (7.99 µmol m^-2^ s^-1^) and Troyer citrange inoculated with mixed strains (T_9_) (7.96 µmol m^-2^ s^-1^) while minimum leaf respiration rates were recorded in Troyer citrange and Cleopatra mandarin co-inoculated with *G*. *intraradices* and PSB (T_6_ and T_18_, respectively) (3.81 and 3.82 µmol m^-2^ s^-1^, respectively), which were statistically comparable to Troyer and Cleopatra seedlings co-inoculated with *G*. *intraradices* and *Azospirillum* sp. (T_7_ and T_19_, respectively) (4.28 and 4.40 µmol m^-2^ s^-1^, respectively).

Under water stress, Cleopatra mandarin control (T_13_) exhibited maximum leaf respiration rate (13.11 µmol m^-2^ s^-1^), which was statistically comparable to PSB inoculated seedlings of Cleopatra mandarin (T_14_) (12.69 µmol m^-2^ s^-1^) whereas minimum leaf respiration rate was recorded in Troyer citrange co-inoculated with *G*. *intraradices* and PSB (T_6_) (7.52 µmol m^-2^ s^-1^), which was statistically comparable to Cleopatra mandarin co-inoculated with *G*. *intraradices* and PSB (T_18_) (7.83 µmol m^-2^ s^-1^) and Troyer citrange co-inoculated with *G*. *intraradices* and *Azospirillum* sp. (T_7_) and Troyer citrange co-inoculated with *G*. *intraradices* and *Providencia* sp. (T_8_) (7.95 and 8.08 µmol m^-2^ s^-1^, respectively).

Under well-watered condition, maximum leaf respiration rate was recorded in Troyer citrange control (T_1_) (8.75 µmol m^-2^ s^-1^), which was statistically comparable to those of Cleopatra mandarin control (T_13_), PSB inoculated Troyer citrange (T_2_) and mixed strain inoculated Cleopatra mandarin (T_21_) (8.58, 8.40 and 8.12 µmol m^-2^ s^-1^, respectively), while Troyer citrange and Cleopatra mandarin co-inoculated with *G*. *intraradices* and PSB (T_6_ and T_18_, respectively) exhibited lowest leaf respiration rate (3.98 µmol m^-2^ s^-1^). (Table 4).

### 3.10. Stomatal conductance

The dual inoculation of AM fungi and helper bacteria was observed to have a synergistic effect on the stomatal behaviour of citrus seedlings under both moisture conditions. The *G*. *intraradices*-induced increase in stomatal conductance in both Troyer citrange and Cleopatra mandarin was high when co-inoculated with PSB culture (T_6_ and T_18_, respectively), just prior to the imposition of differential moisture regimes (0.269 and 0.243 mmol m^-2^ s^-1^, respectively) and also at the end of the 20-day dry-down window under water stress conditions (0.201 and 0.135 mmol m^-2^ s^-1^, respectively). Under water stress, this treatment was statistically comparable to seedlings inoculated with *G*. *intraradices* + *Azospirillum* sp. (T_7_) and mixed strains + PSB (T_10_) (0.193 and 0.194 mmol m^-2^ s^-1^, respectively) in Troyer citrange.

The uninoculated Troyer and Cleopatra seedlings (T_1_ and T_13_, respectively) recorded significantly lower stomatal conductance values before stress (0.128 and 0.031 mmol m^-2^ s^-1^, respectively) and at the end of the 20-day drought treatment (0.084 and 0.006 mmol m^-2^ s^-1^, respectively). Thus, stomatal conductance was sustained at more functional levels under water deficit in *G*. *intraradices* + PSB and *G*. *intraradices* + *Azospirillum* treatments. Conversely, uninoculated control plants showed significantly reduced stomatal conductance, which led to marked photosynthetic inhibition. Drought-induced leaf tissue respiration spiked heavily in control variants but was efficiently suppressed in dually inoculated combinations (Table 4).

### 3.11. Total phenols

Phenols are osmolytes which are found to accumulate in the cytoplasm of leaf under water-stress condition, irrespective of treatment and citrus rootstocks. The study revealed that *G*. *intraradices* + *Azospirillum* sp. treatment significantly accumulated higher phenols in leaf of Troyer citrange (T_7_) (41.23, 46.87 and 36.70 mg 100 g^-1^), as compared to control (T_1_) (17.60, 21.09 and 14.95 mg 100 g^-1^) and in leaf of Cleopatra mandarin (T_19_) (39.86, 46.14 and 34.91 mg 100 g^-1^) over control (T_13_) (15.31, 19.48 and 12.30 mg 100 g^-1^) at BS and under WS and WW conditions, respectively (Table 5).

**Table 5:** Effect of microbial inoculants on osmolyte content of citrus rootstocks under differential soil moisture regimes*.

Treatment	Total phenols (mg 100 g^-1^ F.W.)			Proline (µg g-1)			Total soluble sugars (%)		
	BS	WS	WW	BS	WS	WW	BS	WS	WW
T_1_	17.60^gh^	21.09^g^	14.95^gh^	41.18^i^	53.70^h^	40.64^h^	19.38^i^	26.63^h^	13.05^h^
T_2_	18.69^fg^	22.61^fg^	17.37^f^	58.10^h^	61.44^g^	54.42^g^	22.40^h^	26.89^h^	14.34^gh^
T_3_	19.78^f^	22.12^fg^	18.07^f^	62.28^gh^	68.32^fg^	54.94^g^	28.40^ef^	33.52^e^	15.57^fg^
T_4_	18.34^fh^	22.56^fg^	16.89^f^	73.00^fg^	81.70^e^	65.72^f^	25.48^fg^	31.54^f^	14.70^gh^
T_5_	30.52^d^	34.81^d^	26.97^d^	78.90^de^	91.10^d^	73.50^de^	30.41^de^	33.45^e^	27.42^d^
T_6_	34.89^c^	43.21^b^	33.67^b^	103.20^b^	178.30^b^	90.78^b^	39.32^a^	44.59^b^	34.57^b^
T_7_	41.23^a^	46.87^a^	36.70^a^	80.12^d^	122.30^c^	77.82^cd^	35.51^bc^	38.87cd	30.38^c^
T_8_	36.97^b^	41.21^bc^	30.80^c^	79.98^d^	131.10^c^	76.16^d^	32.40^cd^	37.49^d^	29.53^c^
T_9_	27.15^e^	29.48^e^	22.80^e^	73.82^f^	85.50 ^de^	67.46^ef^	26.40^ef^	29.38^g^	21.40^e^
T_10_	33.38^c^	36.86^cd^	29.38^cd^	78.40^de^	97.66^d^	68.18^ef^	29.58^de^	32.52^ef^	24.47^e^
T_11_	29.12^de^	32.62^de^	24.78^e^	75.56^ef^	89.92^d^	68.04^ef^	28.69^e^	31.62^f^	23.50^e^
T_12_	28.31^e^	31.76^e^	23.76^e^	76.20^ef^	118.72^c^	65.80^f^	28.17^e^	31.93^ef^	22.71^e^
T_13_	15.31^i^	19.48^h^	12.30^i^	46.36^hi^	58.80^gh^	43.26^h^	21.45^h^	28.67^gh^	17.45^f^
T_14_	15.87^hi^	21.10^g^	13.56^hi^	62.18^gh^	66.60^fg^	57.34^g^	23.41^gh^	28.94^gh^	18.06^f^
T_15_	16.10^hi^	21.58^g^	14.80^gh^	65.56^fg^	73.50^f^	56.20^g^	30.49^d^	35.39^de^	19.61^f^
T_16_	16.07^hi^	21.09^g^	15.56^g^	77.30^de^	83.76^e^	69.78^e^	31.83^cd^	37.39^d^	20.39^f^
T_17_	28.59^de^	33.98^de^	24.82^e^	83.06^cd^	94.80^d^	75.56^d^	35.38^bc^	39.31^c^	31.44^bc^
T_18_	33.18^c^	42.13^bc^	31.20^bc^	113.96^a^	184.18^a^	97.94^a^	40.05^a^	47.49^a^	36.63^a^
T_19_	39.86^ab^	46.14^a^	34.91^ab^	89.44^bc^	124.18^bc^	80.54^c^	36.40^b^	42.63^b^	32.45^b^
T_20_	35.00^c^	39.03^c^	29.40^cd^	83.58^cd^	131.68^b^	79.80^c^	35.66^bc^	40.21^bc^	30.54^c^
T_21_	25.26^e^	29.00^e^	20.87^e^	75.16^ef^	88.28^d^	68.12^ef^	32.46^cd^	36.53^d^	25.55^de^
T_22_	31.09^d^	36.11^cd^	26.87^d^	83.30^cd^	99.40^d^	74.30^d^	34.88^bd^	39.39^c^	27.05^d^
T_23_	26.76^e^	31.68^e^	21.73^e^	77.16^de^	94.00^d^	70.88^e^	32.56^cd^	37.51^d^	25.40^de^
T_24_	27.43^e^	30.54^e^	20.77^e^	79.10^de^	120.16^c^	69.50^e^	34.43^bd^	38.63^cd^	26.67^d^

Note: *BS = Before stress, WS = Water deficit stress, WW = Well-watered; Grouping letters (a–i) within a column denote statistically significant differences according to Fisher’s protected LSD test (n = 6, p < 0.05, SPSS 16.0). For treatment details, please see Table 1.

### 3.12. Proline

Leaf proline content was found to increase in leaves of both the citrus rootstocks under water-stress condition, which helps to protect membranes and protein from dehydration. AM plants were found to have high proline content at both WS and WW condition and thus helps in maintaining high water relation of plants. Among different treatment, dual inoculation of *G*. *intraradices* and PSB caused significantly increased level of leaf proline in Troyer citrange (T_6_) (103.20, 178.30 and 90.78 µg g^-1^) over uninoculated control (T_1_) (41.18, 53.70 and 40.64 µg g^-1^) and in Cleopatra mandarin (T_18_) (113.96, 184.18 and 97.94 µg g^-1^) over control (T_13_) (46.36, 58.80 and 43.26 µg g^-1^) at BS and under WS and WW conditions, respectively (Table 5).

### 3.13. Total soluble sugars

Water-stress also caused more accumulation of total soluble sugars in leaf than WW plants. Troyer citrange seedlings inoculated with *G*. *intraradices* + PSB (T_6_) significantly recorded highest total soluble sugars in leaf (39.32, 44.59 and 34.57%) while control seedlings (T_1_) recorded lowest (19.38, 26.63 and 13.05%) at BS and under WS and WW conditions, respectively. In case of Cleopatra mandarin, the highest total soluble sugars content was found in *G*. *intraradices* + PSB (T_18_) (40.05, 47.49 and 36.63%) while significantly lowest total soluble sugars content was found in uninoculated control (T_13_) (21.45, 28.67 and 17.45%) at BS and under WS and WW conditions, respectively (Table 5).

### 3.14. Ascorbic acid

Ascorbic acid content in citrus rootstocks was more under WW condition, than under WS condition (Table 6). Before stress, Troyer seedlings inoculated with *G*. *intraradices* + PSB culture (T_6_) had highest ascorbic acid content (287.00 µmol g^-1^), which was at par with *G*. *intraradices* + *Providencia* sp. (T_8_) (273.50 µmol g^-1^), as compared to control (T_1_) (127.50 µmol g^-1^). Cleopatra seedlings inoculated with *G*. *intraradices* + PSB (T_18_) and *G*. *intraradices* + *Providencia* sp. (T_20_) had highest ascorbic acid content (269.50 and 257.50 µmol g^-1^, respectively), as compared to control (T_13_) (117.50 µmol g^-1^). Under WS condition, *G*. *intraradices* + *Providencia* culture had significant influence on increase in ascorbic acid content in leaves of both Troyer citrange (T_8_) and Cleopatra seedlings (T_20_) (250.50 and 237.50 µmol g^-1^, respectively), which was at par with *G*. *intraradices* + PSB treatment (T_6_ and T_18_, respectively) (243.50 and 228.50 µmol g^-1^, respectively), as compared to uninoculated Troyer and Cleopatra seedlings (T_1_ and T_13_, respectively) (108.50 and 100.50 µmol g^-1^, respectively). Under WW condition, *G*. *intraradices* and PSB had synergistic effect on increase in ascorbic acid concentration in leaves of Troyer citrange (T_6_) and Cleopatra mandarin seedlings (T_18_) (363.50 and 342.50 µmol g^-1^, respectively), which was at par with *G*. *intraradices* + *Azospirillum* sp. (T_7_ and T_19_, respectively) (351.50 and 328.50 µmol g^-1^, respectively), as compared to uninoculated Troyer and Cleopatra seedlings (T_1_ and T_13_, respectively) (169.50 and 159.50 µmol g^-1^, respectively).

**Table 6:** Effect of microbial inoculants on non-enzymatic antioxidant levels of citrus rootstocks under differential soil moisture regimes*.

Treatment	Ascorbic acid (µmol g^-1^ F.W.)			Total carotenoids (mg g^-1^ F.W.)			Glutathione (µmol g^-1^ F.W.)		
	BS	WS	WW	BS	WS	WW	BS	WS	WW
T_1_	127.50^fg^	108.50^f^	169.50^f^	0.55^de^	0.56^ef^	0.49^e^	9.56^e^	9.23^f^	10.01^f^
T_2_	134.00^f^	114.50^f^	175.00^f^	0.58^cd^	0.60^de^	0.52^de^	9.85^de^	9.42^ef^	10.35^e^
T_3_	142.50^ef^	120.00^ef^	188.50^ef^	0.61^bc^	0.64^cd^	0.54^cd^	10.12^cd^	9.68^de^	10.58^de^
T_4_	139.00^f^	118.50^f^	182.00^f^	0.59^cd^	0.62^cd^	0.53^d^	9.94^d^	9.55^e^	10.42^de^
T_5_	212.50^c^	194.50^c^	288.50^c^	0.64^b^	0.71^b^	0.62^b^	10.84^b^	10.45^c^	11.64^bc^
T_6_	287.00^a^	243.50^ab^	363.50^a^	0.68^ab^	0.80^a^	0.72^a^	11.85^a^	11.50^a^	12.94^a^
T_7_	264.50^b^	222.00^b^	351.50^a^	0.72^a^	0.74^b^	0.67^ab^	11.31^ab^	10.98^b^	12.23^b^
T_8_	273.50^ab^	250.50^a^	338.50^b^	0.66^b^	0.73^b^	0.65^b^	11.24^b^	11.02^b^	12.01^b^
T_9_	185.00^d^	162.00^d^	242.00^d^	0.58^cd^	0.61^cd^	0.53^d^	10.42^c^	10.01^d^	11.08^cd^
T_10_	208.50^c^	188.50^c^	274.00^c^	0.62^bc^	0.68^bc^	0.59^bc^	10.70^b^	10.29^cd^	11.42^c^
T_11_	194.50^cd^	174.50^cd^	258.00^cd^	0.60^cd^	0.65^cd^	0.56^cd^	10.55^bc^	10.14^d^	11.21^c^
T_12_	190.50^d^	170.00^d^	252.50^d^	0.59^cd^	0.63^cd^	0.55^cd^	10.48^c^	10.08^d^	11.14^cd^
T_13_	117.50^g^	100.50^g^	159.50^g^	0.41^h^	0.49^g^	0.44^g^	9.52^e^	9.31^ef^	9.95^f^
T_14_	122.00^g^	104.00^fg^	164.00^fg^	0.44^gh^	0.52^fg^	0.46^fg^	9.74^de^	9.48^ef^	10.18^ef^
T_15_	131.50^f^	111.00^f^	176.50^f^	0.48^f^	0.55^ef^	0.48^ef^	10.01^cd^	9.62^e^	10.44^de^
T_16_	128.50^fg^	107.50^f^	171.00^f^	0.46^fg^	0.53^fg^	0.47^fg^	9.88^d^	9.52^e^	10.31^e^
T_17_	198.50^c^	178.50^c^	268.00^c^	0.50^ef^	0.64^cd^	0.52^de^	10.64^bc^	10.22^d^	11.35^c^
T_18_	269.50^ab^	228.50^b^	342.50^ab^	0.54^de^	0.65^c^	0.54^cd^	11.85^a^	11.58^a^	12.72^a^
T_19_	248.50^b^	212.00^bc^	328.50^b^	0.49^ef^	0.59^de^	0.54cd	11.68^a^	11.38^a^	12.44^ab^
T_20_	257.50^ab^	237.50^a^	318.50^b^	0.48^f^	0.61^cd^	0.51^de^	11.12^b^	10.84^b^	11.95^b^
T_21_	168.50^d^	148.50^d^	224.50^d^	0.45^fg^	0.54^ef^	0.47^fg^	10.24^c^	9.85^d^	10.88^cd^
T_22_	192.50^cd^	172.50^cd^	254.50^cd^	0.48^f^	0.58^de^	0.51^de^	10.55^bc^	10.12^d^	11.24^c^
T_23_	178.50^d^	158.50^d^	238.50 ^d^	0.47^fg^	0.56^ef^	0.49^e^	10.38^c^	9.94^d^	11.02^cd^
T_24_	184.50^d^	164.50^d^	246.00^d^	0.46^fg^	0.55^ef^	0.50^e^	10.44^c^	10.02^d^	11.11^cd^

Note: *BS = Before stress, WS = Water deficit stress, WW = Well-watered; Letters (**a–h**) indicate separate structural groupings based on LSD multiple post-hoc analysis (n = 6, α = 0.05, SPSS 16.0). For treatment details, please see Table 1.

### 3.15. Carotenoids

Carotenoids (β-carotene + xanthophylls), the tetraterpenes, known for their ability to detoxify various forms of activated oxygen, was enhanced under WS condition in both the citrus rootstocks. At BS, total carotenoids were maximum in leaves of Troyer citrange co-inoculated with *G*. *intraradices* and *Azospirillum* sp. (T_7_) and *G*. *intraradices* and PSB (T_6_) (0.72 and 0.68 mg g^-1^, respectively) while minimum recorded in leaves of control and PSB inoculated seedlings of Cleopatra mandarin (T_13_ and T_14_, respectively) (0.41 and 0.44 mg g^-1^, respectively).

Under water stress, Troyer citrange co-inoculated with *G*. *intraradices* and PSB (T_6_) had higher content of total carotenoids (0.80 mg g^-1^) while control (T_13_) and PSB inoculated seedlings (T_14_) and *Providencia* sp. inoculated seedlings (T_16_) of Cleopatra mandarin exhibited minimum content of total carotenoids (0.49, 0.52 and 0.53 mg g^-1^, respectively).

Under well-watered condition, highest leaf carotenoid content was observed in Troyer citrange co-inoculated with *G*. *intraradices* and PSB (T_6_) (0.72 mg g^-1^), which was statistically comparable to that of Troyer citrange co-inoculated with *G*. *intraradices* and *Azospirillum* sp. (0.67 mg g^-1^) while lowest carotenoid content was recorded in Cleopatra mandarin control (T_13_) (0.44 mg g^-1^), which was statistically comparable to seedlings inoculated with PSB, *Providencia* sp. and mixed strains (T_14_, T_16_ and T_21_, respectively) (0.46, 0.47 and 0.47 mg g^-1^, respectively) (Table 6).

### 3.16. Glutathione

Leaf glutathione content was highest in seedlings co-inoculated with *G*. *intraradices* and PSB for both Troyer citrange (T_6_) (11.85, 11.50 and 12.94 µmol g^-1^) and Cleopatra mandarin (T_18_) (11.85, 11.58 and 12.72 µmol g^-1^) at BS and under water stress and well-watered condition, respectively, which was statistically comparable to Troyer citrange co-inoculated with *G*. *intraradices* and *Azospirillum* sp. (T_7_) at BS (11.31 µmol g^-1^) and Cleopatra mandarin co-inoculated with *G*. *intraradices* and *Azospirillum* sp. (T_19_) at BS and under water stress and well-watered condition (11.68, 11.38 and 12.44 µmol g^-1^, respectively).

Its content in leaf was minimum in control seedlings of Troyer citrange (T_1_) (9.56, 9.23 and 10.01 µmol g^-1^) and Cleopatra mandarin (T_13_) (9.52, 9.31 and 9.95 µmol g^-1^, respectively) at BS and under water stress and well-watered condition, respectively, which was statistically comparable to Troyer citrange inoculated with PSB (T_2_) at BS and under water stress (9.85 and 9.42 µmol g^-1^, respectively) and Cleopatra mandarin inoculated with PSB (T_14_) at BS and under water stress and well-watered condition (9.74, 9.48 and 10.18 µmol g^-1^, respectively) (Table 6).

### 3.17. Abscisic acid

The accumulation of leaf tissue abscisic acid (ABA) was found to be increased under water-stress condition. However, absolute leaf tissue ABA concentrations were significantly lower in Troyer citrange seedlings inoculated with *G*. *intraradices* + *Azospirillum* sp. (T_7_), *G*. *intraradices* + PSB (T_6_), *G*. *intraradices* + *Providencia* sp. (T_8_) and mixed strains + *Azospirillum* sp. (T_11_) (3.517, 3.589, 3.946 and 4.010 µg g^-1^ F.W., respectively) and Cleopatra seedlings inoculated with *G*. *intraradices* + PSB (T_18_), *G*. *intraradices* + *Azospirillum* sp. (T_19_) and *G*. *intraradices* + *Providencia* sp. (T_20_) (3.079, 3.499 and 3.301 µg g^-1^ F.W., respectively), at just before imposing differential moisture regimes. At this stage, the uninoculated Troyer and Cleopatra seedlings (T_1_ and T_13_, respectively) exhibited highest abscisic acid content in leaf (6.919 and 5.993 µg g^-1^ F.W., respectively), which was at par with Troyer seedlings inoculated with PSB culture (T_2_) (5.993 µg g^-1^ F.W.).

Under water deficit stress condition, leaf tissue hormonal accumulation was strongly modified (F_1, 264_ = 118.4, *p* < 0.001). The leaf ABA concentration was found to be highest in uninoculated Troyer citrange (T_1_) and Cleopatra mandarin seedlings (T_13_) (10.219 and 10.054 µg g^-1^ F.W., respectively, which was statistically at par with *Providencia* sp. and *Azospirillum* sp. in case of Troyer citrange (T_4_ and T_3_, respectively) (10.064 and 9.947 µg g^-1^ F.W., respectively) and Cleopatra mandarin (T_16_ and T_15_, respectively) (9.916 and 9.799 µg g^-1^ F.W., respectively). To account for pre-existing baseline variations prior to the dry-down period, evaluating the stress response as a percentage change (%Δ) relative to each treatment’s specific before-stress (BS) baseline confirmed that co-inoculated groups restricted their leaf ABA escalation rate significantly compared to uninoculated control lines (p < 0.05). The dual inoculation of *G*. *intraradices* and PSB significantly recorded lowest ABA content in Troyer citrange and Cleopatra mandarin (T_6_ and T_18_, respectively) (6.079 and 5.250 µg g^-1^ F.W., respectively, followed by Troyer and Cleopatra seedlings inoculated with *G*. *intraradices* + *Azospirillum* sp. (T_7_ and T_19_, respectively) (7.002 and 6.742 µg g^-1^ F.W., respectively).

Under well-watered conditions, *G*. *intraradices* + PSB dual inoculation resulted in significantly lower leaf tissue ABA concentrations in both Troyer and Cleopatra seedlings (T_6_: 2.187 and T_18_: 2.089 μg g^-1^ F.W., respectively), which was statistically comparable to Troyer seedlings inoculated with *G*. *intraradices* + *Providencia* sp. (T_8_: 2.672 μg g^-1^ F.W.) and Cleopatra seedlings dually inoculated with *G*. *intraradices* + *Providencia* sp. (T_20_: 2.307 μg g^-1^ F.W.) or *G*. *intraradices* + *Azospirillum* sp. (T_19_: 2.630 μg g^-1^ F.W.). The highest leaf ABA content was found in uninoculated control in Troyer citrange and Cleopatra mandarin (T_1_ and T_13_, respectively) (5.838 and 5.508 µg g^-1^ F.W., respectively, which was at par with single inoculation of *Providencia* sp. (T_16_) (5.505 µg g^-1^ F.W.), in case of Cleopatra mandarin (Fig 3).

**Fig 3:**
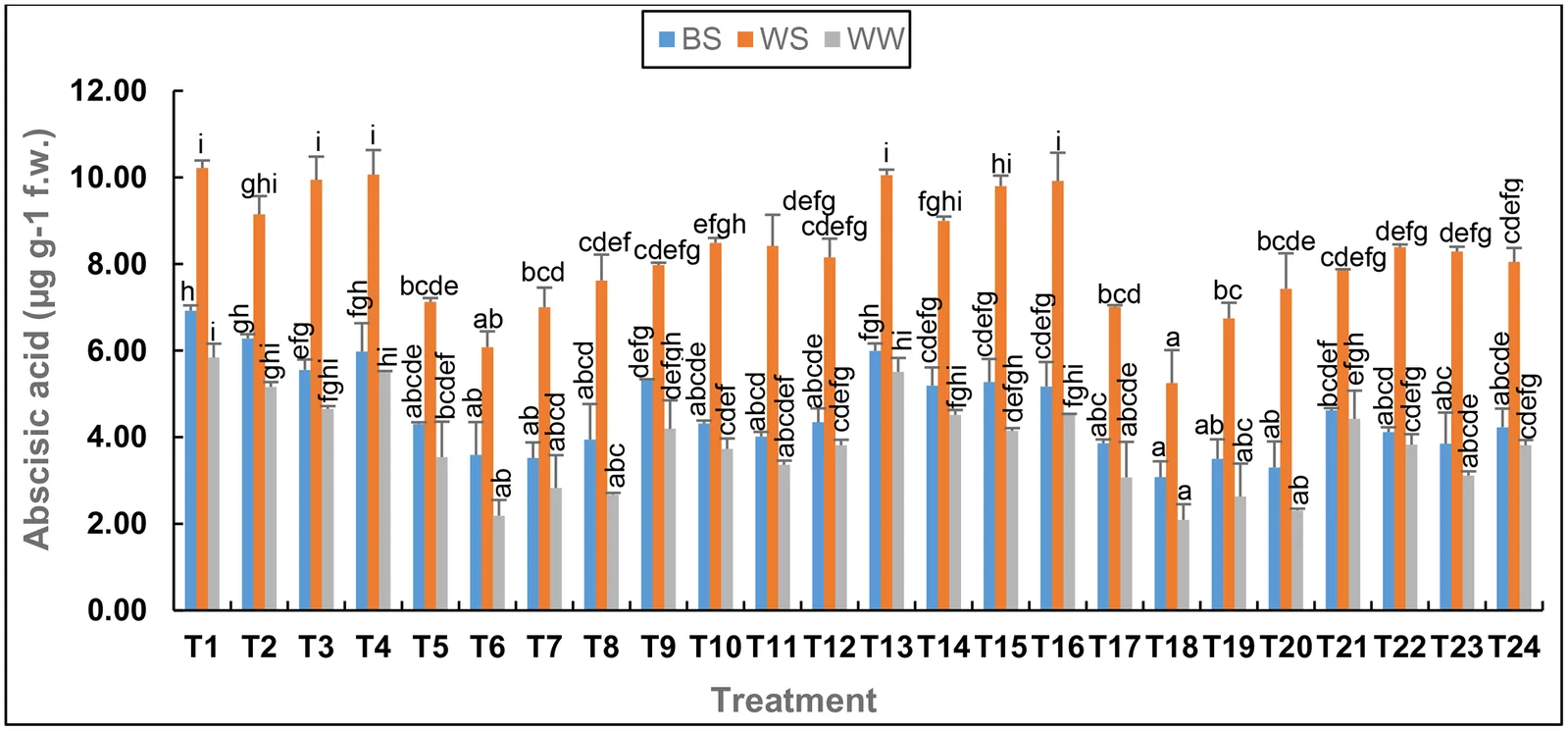
Abscisic acid content in the leaf of citrus rootstocks as influenced by AMF and MHB at before and after imposing differential soil moisture regimes. *{Note: BS = Before stress, WS = Water deficit stress, WW = Well-watered; Grouping letters (a–i) within a column denote statistically significant differences according to Fisher’s protected LSD test (n = 6, p < 0.05, SPSS 16.0). For treatment details, please see* Table 1*}.*

### 3.18. Pearson correlation analysis

Bivariate relationship modeling conducted via Pearson correlation analysis (2-tailed significance testing) highlighted a substantial positive correlation between the percentage of root colonization in citrus rootstocks and the majority of parameters. Strong positive links were verified with leaf relative water content (*r* = 0.939, *p* < 0.01), net photosynthetic rate (*r* = 0.863, *p* < 0.01), total leaf phenols (*r* = 0.969, *p* < 0.01), leaf proline (*r* = 0.866, *p* < 0.01), total leaf soluble sugars (*r* = 0.855, *p* < 0.01), leaf ascorbic acid (*r* = 0.966, *p* < 0.01), total carotenoids (*r* = 0.883, *p* < 0.01), and leaf glutathione (*r* = 0.963, *p* < 0.01). Pearson correlation mapping also confirmed a powerful negative correlation between the root colonization percentage and the final leaf tissue ABA concentration (*r* = −0.941, *p* < 0.01). These coefficients were evaluated across the 24 treatment mean nodes to track broad physiological trends across microbial and moisture matrices (Table 7).

**Table 7:** Pearson correlation coefficients (r) among root colonization and leaf physio-biochemical traits.

Physiological / Biochemical Trait Node (n = 24)	Pearson Coefficient (r) with Root Colonization (%)
Leaf relative water content (%)	0.939**
Net photosynthetic rate (µmol m^-2^ s^-1^)	0.863**
Total phenols content (mg 100 g^-1^ F.W.)	0.969**
Leaf proline content (µg g^-1^)	0.866**
Total soluble sugars (%)	0.855**
Ascorbic acid content (µmol g^-1^ F.W.)	0.966**
Total carotenoids (mg g^-1^ F.W.)	0.883**
Glutathione content (µmol g^-1^ F.W.)	0.963**
Leaf abscisic acid concentration (µg g^-1^ F.W.)	−0.941**

Note: Correlations were calculated using the treatment mean nodes (n = 24) derived from pooled annual runs. Within the experimental setup, each individual potted seedling served as a distinct independent experimental unit, yielding six true biological replications per treatment across the pooled dataset. Significance indicators: * details p < 0.05; ** details p < 0.01 (SPSS 16.0).

## 4. Discussion

### 4.1. Root colonization and water relations

Low soil moisture stress had a strong effect on AM development. However, co-inoculating citrus rootstocks with specialized AMF strains and mycorrhizal helper bacteria triggers a robust physiological buffer that successfully counteracts severe drought stress. Our investigation revealed that the Troyer citrange and Cleopatra mandarin seedlings recorded significantly highest root colonisation at just before imposing differential water treatment and after 20 days of differential water treatment for both water-stressed and well-watered plants, when dually inoculated with *G*. *intraradices* and PSB. Many workers have shown that bacteria or bacteria-like organelles are intimately associated with AMF spores, and have the capacity to inhibit or stimulate the germination of AMF spores [22,23]. Decrease in mycorrhizal inoculation under water-stress condition might be due to a decrease in spore stress, hypha growth and effect on formation of arbuscule [24]. Our study revealed that root colonization was 4.17% higher in Troyer citrange rootstock than in cleopatra mandarin, though statistically insignificant. The higher root colonization by AM fungi in Troyer citrange compared to Cleopatra mandarin was attributed to the fact that Troyer citrange naturally possessed a finer root system with a greater specific root length while Cleopatra mandarin featured a coarser, thicker root system with fewer fine lateral extensions, offering fewer immediate structural niches for the fungus to colonize aggressively [25,26].

In our study, although water-stress treatments reduced leaf relative water content, inoculation by AM fungi *G*. *intraradices* and PSB resulted in slower decline of soil moisture content and also leaf relative water content compared with other treatments, including un-inoculated control. It might be due to increase in mycorrhizal activity due to synergistic effect of helper bacteria PSB in the rhizosphere, which helped in better development of water-stable aggregates in the rhizosphere, compared to other treatments. Oliveira and Oliveira [27] found that soil moisture content was positively correlated with AM fungal colonization. Our study found that Troyer citrange seedlings co-inoculated with *G*. *intraradices* and PSB (T_6_) and those co-inoculated with *G*. *intraradices* and *Providencia* sp. (T_8_) had 27.68 and 26.97% higher leaf relative water content compared to control (T_1_) while Cleopatra mandarin co-inoculated with *G*. *intraradices* and PSB (T_18_) recorded 33.26% more leaf relative water content than control (T_13_) under water stress condition. The increase in leaf relative water content in *G*. *intraradices* and PSB inoculated citrus seedlings might be due to ability to take up more water due to well-developed root system of dual inoculated citrus seedlings, as compared to other treatments, including un-inoculated control. The enhancement of root growth by bacterial inoculation could be due to IAA produced by bacteria [28]. The AM fungi exhibited greater extraradical mycelium (glomalin) and plant water uptake probably as a result of an improvement in root conductivity to water ﬂow and/or via a mycelium able to transport water to the AM colonized root system [29]. As a result, the increased root growth due to effective AM colonization increased plant water content under water-stress conditions [30]. Mycorrhizal symbiosis had been shown to increase drought tolerance, although the mechanisms involved were contentious [31]. In the condition of water deficit stress, AM symbiosis usually postponed declines in leaf water potential [32,33]. Tripathi et al. [34] found positive mycorrhization effect on leaf water potential of papaya plant, when inoculated with *G*. *intraradices* and PSB under water-stress conditions. Our study also revealed that Troyer citrange had respectively 6.77 and 1.19% higher leaf relative water content than Cleopatra mandarin control seedlings and those co-inoculated with *G*. *intraradices* and PSB under water stress, though statistically insignificant. Thus, Troyer citrange maintained a higher leaf relative water content under water stress compared to Cleopatra mandarin due to differences in root system vigor, stomatal behavior, and osmotic adjustments [35].

In our study, we observed 130.17% higher leaf area in Cleopatra mandarin co-inoculated with *G*. *intraradices* and PSB (T_18_) compared to control (T_13_), which was statistically comparable to seedlings co-inoculated with *G*. *intraradices* and *Azospirillum* sp. (T_19_), seedlings co-inoculated with *G*. *intraradices* and *Providencia* sp. (T_20_) and those co-inoculated with mixed strains and *Azospirillum* sp. (T_23_) (108.83, 103.16 and 102.77%, respectively) under water stress condition, which could be attributed to the fact that combination of AM fungi and mycorrhiza helper bacteria improved water uptake, nutrient acquisition, and cellular protection far better than single inoculation or no treatment [36]. It was evident from our study that Cleopatra mandarin had 19.34 and 50.21% more leaf area compared to Troyer citrange in control and seedlings co-inoculated with *G*. *intraradices* and PSB under water stress condition, though statistically insignificant for control seedlings. Thus, Cleopatra mandarin developed entire, broader leaves, whereas Troyer citrange typically presented leaves with smaller overall blade dimensions [37].

### 4.2. Photosynthetic pigments and gas exchange

Water-stress can cause an adverse effect on chlorophyll content of plant leaf. Plants experiencing severe saline or water-stress in their native environments do not become greener [38]. The loss of leaf chlorophyll concentration in citrus plants under water deficit has been reported previously by Wu and Xia [39]. We found that total chlorophyll was improved by 78.38 and 76.64% in both Troyer citrange and Cleopatra mandarin co-inoculated with *G*. *intraradices* and PSB (T_6_ and T_18_, respectively), as compared to control (T_1_ and T_13_, respectively) under water stress condition. This was possibly due to the fact that at low soil water potential, mycorrhizal plants could absorb more water than non-mycorrhizal ones, as mentioned by Porcel and Ruiz-Lozano [40]. These results were in agreement with those previously found by Morte et al. [41], Sanchez–Blanco et al. [42] and Beltrano and Ronco [43] who detected higher chlorophyll concentrations in mycorrhizal plants subjected to water deficit stress. In addition, Mathur and Vyas [44] concluded that arbuscular mycorrhizal root colonization increased chlorophyll synthesis, which could be associated with higher photosynthesis rates and plant growth. We observed higher leaf chlorophyll content in Troyer citrange than Cleopatra mandarin by 8.03 and 9.09% in both control and seedlings co-inoculated with *G*. *intraradices* and PSB, respectively, under water stress, though statistically insignificant for control seedlings. Troyer citrange displayed higher leaf chlorophyll content than Cleopatra mandarin due to its genetic makeup and vigorous growth capacity [45].

Arbuscular mycorrhizal plants often show higher photosynthetic rates than their experimental NM counterparts, which is consistent with AM effects on stomatal conductance [1]. Maintenance of greater photosynthetic capacity during water deficit stress in mycorrhizal plants had also been indicated by higher starch levels in leaves of AM than in control plants [46]. Barzana et al. [47] found that the efficiency of photosystem II was maximum in maize seedlings inoculated with *G*. *intraradices* under well-watered conditions. The water deficit stress imposed reduced the efficiency of photosystem II in *G*. *intraradices*¬-inoculated maize seedlings, but remained higher than in non-AM seedlings subjected to water deficit stress. Sheng et al. [48] found that root colonization by *G*. *mosseae* enhanced net photosynthetic rate and stomatal conductance and reduced intercellular CO_2_ concentration of the leaves of maize plants grown under salt stress condition. Increased CO2 assimilation had been considered a plant strategy for drought tolerance [49]. It had been proved that concentration of chlorophyll in AM plants was higher than their control NM plants [50,51] and higher concentration of chlorophyll was associated with higher photosynthesis rate [46]. Similarly, changes in stomatal conductance [3] had been reported to be mechanisms by which AM fungus-colonized plants increased water deficit stress resistance. Stomatal closure is one of the mechanisms that plants use to protect themselves from excessive water loss. Nikolaou et al. [51] reported that mycorrhizal grape vines maintained higher stomatal conductance (more open stomata) than non-mycorrhizal vines. Our study showed that net photosynthesis was higher by 496.06% in Troyer citrange co-inoculated with *G*. *intraradices* and PSB (T_6_) compared to control (T_1_) under water stress. Leaf stomatal conductance was 139.29% higher in Troyer citrange co-inoculated with *G*. *intraradices* and PSB (T_6_) compared to control (T_1_), which was statistically comparable to seedlings co-inoculated with mixed strains and PSB (T_10_) and those co-inoculated with *G*. *intraradices* and *Azospirillum* sp. (T_7_) (130.95 and 129.76%, respectively), under water stress. Leaf respiration rate was respectively lesser by 38.06 and 40.27% in both Troyer citrange and Cleopatra mandarin co-inoculated with *G*. *intraradices* and PSB (T_6_ and T_18_) compared to control (T_1_ and T_13_), which was statistically comparable to those of Troyer citrange co-inoculated with *G*. *intraradices* and *Azospirillum* sp. (T_7_) and *G*. *intraradices* and *Providencia* sp. (T_8_) under water stress (34.51 and 33.44% lesser than control, respectively). One of the most common explanations for the improved physiological status in mycorrhizal seedlings is the strongly increased absorbing surface caused by soil-growing hyphae combined with the fungal ability to take up water from soils with low water potential [52]. The increment in photosynthetic rate and stomatal conductance induced by phosphorus solubilizing bacterial inoculation of *G*. *intraradices*-colonized plant was also reported by Vivas et al. [53], which seems to be due to a specific microbe-microbe interaction that modulates the effectiveness of each AMF on plant physiology. Our study found that Troyer citrange had 3.25 and 17.36% more net photosynthetic rate than Cleopatra mandarin in both control and seedlings co-inoculated with *G*. *intraradices* and PSB, respectively, under water stress, though statistically insignificant for control seedlings. We also found 1300.00 and 48.89% more leaf stomatal conductance in control and seedlings co-inoculated with *G*. *intraradices* and PSB of Troyer citrange, as compared to those of cleopatra mandarin, under water stress. We also noticed 7.40 and 3.96% lesser leaf respiration rate in both control and seedlings co-inoculated with *G*. *intraradices* and PSB of Troyer citrange compared to those of Cleopatra mandarin, under water stress, though statistically insignificant for microbial inoculated seedlings. Thus, Troyer citrange maintained higher photosynthesis, lower respiration and stomatal conductance under water stress because it exhibited an anisohydric behavior, keeping its stomata wider open to facilitate continuous carbon dioxide uptake, whereas Cleopatra mandarin acted isohydric, closing its stomata tightly to conserve water [54].

### 4.3. Osmotic adjustment

We found that dual inoculated treatment of *G*. *intraradices* and *Azospirillum* significantly accumulated higher concentration of osmolytes like total phenols in leaf of Troyer citrange and Cleopatra mandarin at just before imposing stress and also under water-stress and well-watered condition. Total phenols were 122.24 and 136.86% more in Troyer citrange and Cleopatra mandarin co-inoculated with *G*. *intraradices* and *Azospirillum* sp. (T_7_ and T_19_, respectively) compared to control (T_1_ and T_13_, respectively) under water stress. Eftekhari et al. [55] found that grape vines inoculated with AM fungi had significantly higher content of total phenols in the foliage as compared to un-inoculated control, indicating enhancement in defence mechanism of inoculated plants against diseases, which led to increased plant survival under nursery or glasshouse as well as field conditions [56]. We found higher content of total phenols in leaves of control and seedlings co-inoculated with *G*. *intraradices* and *Azospirillum* sp. of Troyer citrange than those of Cleopatra mandarin (8.26 and 1.58%, respectively) under water stress, though statistically insignificant for microbial inoculated seedlings.

In this study, a greater leaf proline content in AM fungi inoculated citrus seedlings was reported, which was in agreement with the findings of Wu and Xia [39], who suggested that AM symbiosis could improve the osmotic adjustment by accumulation of proline which help to maintain high osmotic levels in plants. In addition, proline might act as a sink for energy to regulate redox potentials and a hydroxyl radical scavenger to alleviate oxidative stress in AM fungi inoculated citrus seedlings [57]. In our study, leaf proline content was higher by 213.23% in Cleopatra mandarin co-inoculated with *G*. *intraradices* and PSB (T_18_) compared to control (T_13_) under water stress. The increase in leaf proline content due to AM fungi and MHB under moisture stress condition was also reported by other authors [58–60]. We observed 9.50 and 3.30% more leaf proline in control and seedlings co-inoculated with *G*. *intraradices* and PSB of Cleopatra mandarin than those of Troyer citrange under water stress, though statistically insignificant for control seedlings.

It was evident from our investigation that citrus seedlings inoculated with *G*. *intraradices* and PSB had significantly higher total soluble sugars than control seedlings regardless of soil water status. The results indicated that photosynthetic carbohydrate, especially total soluble sugars, were conducive to be retained in AM seedlings by AM colonization. Our study recorded 65.64% more total soluble sugars in leaves of Cleopatra mandarin co-inoculated with *G*. *intraradices* and PSB (T_18_) than those of control (T_13_) under water stress. The result is in harmony with the findings of Wu et al. [61]. Abbaspour et al. [62] found that Pistacia vera seedlings colonized by *G*. *etunicatum* consistently had a higher content of soluble sugars in leaves than non-AM under water-stress conditions, which might be due to the sink effect of the mycorrhizal fungus demanding sugars from shoot tissues. This beneficial response of osmotic solutes to AMF colonization would help to enhance drought tolerance of citrus seedlings by osmotic adjustment thus protecting and stabilizing macromolecules and structures from damage induced by water deficit stress [63]. We found 7.66 and 6.50% more total soluble sugars in leaves of control and seedlings co-inoculated with *G*. *intraradices* and PSB of Cleopatra mandarin than those of Troyer citrange, respectively under water stress, though statistically insignificant for control seedlings. Thus, Troyer citrange accumulated higher osmolytes than Cleopatra mandarin under water stress because it relied on aggressive osmotic adjustment and higher stomatal conductance to maintain tissue hydration and post-stress recovery [35].

### 4.4. Antioxidant response

In our investigation, decrease in total carotenoids content in citrus leaves was noticed as a result of water-stress. Carotenoids are tetra-terpenoid compositions that maintain chlorophyll against light oxidation, light absorption and transfer of light energy to chlorophyll *a* [64]. One reason for decrease in carotenoids during water-stress might be *β*‐carotene destruction and zeaxanthin formation, as reported in rice seedlings under salinity stress [65]. Our study revealed that leaf ascorbic acid was 130.88% more in Troyer citrange co-inoculated with *G*. *intraradices* and *Providencia* sp. (T_8_) compared to control (T_1_), which was statistically comparable to those of Troyer citrange co-inoculated with *G*. *intraradices* and PSB (T_6_) (124.42% more than Troyer citrange control) and Cleopatra mandarin co-inoculated with *G*. *intraradices* and *Providencia* sp. (T_20_) (136.32% more than Cleopatra mandarin control) under water stress. We observed respectively 42.86 and 32.65% higher total carotenoids in leaves of seedlings co-inoculated with *G*. *intraradices* and PSB of Troyer citrange and Cleopatra mandarin (T_6_ and T_18_), as compared to control (T_1_ and T_13_) under water stress. Leaf glutathione content was higher by 24.59 and 24.38% in seedlings co-inoculated with *G*. *intraradices* and PSB for Troyer citrange and Cleopatra mandarin (T_6_ and T_18_, respectively), as compared to control (T_1_ and T_13_, respectively), which was statistically comparable to those of Cleopatra mandarin co-inoculated by *G*. *intraradices* and *Providencia* sp. (T_19_), under water stress (22.23% more than Cleopatra mandarin control). Enteshari and Hajbagheri [66] also found that seedlings of *Ocimum basilicum* inoculated with *G*. *intraradices* had higher content of total carotenoids than control seedlings. Wu et al. [67] also found that the increments in reactive oxygen species concentration in citrus leaves due to water-stress were generally higher in non-AM than in AM seedlings, and inoculation of helper bacteria had synergistic effect for AM plants for higher ascorbic acid and glutathione. We found leaf glutathione content was more in control seedlings and those co-inoculated with *G*. *intraradices* and PSB and those co-inoculated with *G*. *intraradices* and *Azospirillum* sp. of Cleopatra mandarin than Troyer citrange (0.87, 0.70 and 3.64% more, respectively) under water stress, though statistically insignificant. Under water stress, Troyer citrange maintained a lower total glutathione compared to Cleopatra mandarin, which might be due to its vastly superior and more coordinated antioxidant defence system [68]. Our study witnessed more ascorbic acid content in leaves of control, seedlings co-inoculated with *G*. *intraradices* and PSB and those co-inoculated with *G*. *intraradices* and *Providencia* sp. of Troyer citrange than those of Cleopatra mandarin (7.96, 6.56 and 5.47%, respectively) under water stress, though statistically insignificant. Our results indicated that leaves of Troyer citrange had higher total carotenoids, as compared to those of Cleopatra mandarin, under water stress, for both control and seedlings co-inoculated with *G*. *intraradices* and PSB (14.29 and 23.08% more, respectively). Thus, Troyer citrange accumulated higher levels of ascorbic acid and total carotenoids under water stress than Cleopatra mandarin due to its stronger, more active antioxidant defence and distinct stress-signalling pathways [26].

### 4.5. Hormonal regulation

Under water-stress, ABA levels increased in all plant organs, however, it was less in plants inoculated with *Glomus* fungi than the un-inoculated plants. In our study, co-inoculation of *G*. *intraradices* and PSB resulted in reduction in leaf ABA content by 40.51% in Troyer citrange and 47.78% in Cleopatra mandarin. Kandowangko et al. [69] also found that the combined application of AM fungi and helper bacteria significantly decreased leaf ABA content as compared to control maize seedlings, under water-stress condition. The reduction of ABA content in water deficit stressed plant inoculated by AM fungi and helper bacteria might be in associated to the enhanced development of fungal hypha due to co-inoculation with helper bacteria, which assisted plant to extract water and essential nutrients under dry conditions. This result indicated that the inoculums of AM fungi and helper bacteria could work synergistically to reduce ABA concentration in water-stressed citrus seedlings and thus the dual inoculation of beneficial microorganisms to the water deficit stressed plant was able to alleviate the strained by manipulation of stomatal conductance so that the stomata were still remained open for the longer period. Thus, it is evident that heightened soil moisture retention and leaf RWC values seen in the *G*. *intraradices* + PSB groups indicate that treated rootstocks experienced significantly less severe localized dehydration. This minimized the upstream trigger for massive foliar ABA biosynthesis. Our study observed that Troyer citrange had lesser content of leaf ABA compared to Cleopatra mandarin in both control and seedlings co-inoculated with *G*. *intraradices* and PSB by 1.61 and 13.64%, respectively, though statistically insignificant. Troyer citrange maintained lower levels of ABA in its leaves compared to Cleopatra mandarin primarily due to enhanced genetic and enzymatic catabolism of active ABA [70].

The correlation analysis revealed significant positive amelioration of physio-biochemical traits in plants such as improved photosynthetic rate, accumulation of osmoprotectants (such as enhanced accumulation of proline, total phenols, total sugars, ascorbic acid and glutathione level) and control of ABA metabolism [71,72].

However, the efficacy of soil microbial inoculants remains unreliable, and unlike crop breeding, in which target traits (e.g., yield) have long been considered alongside environmental compatibility, microbial inoculant ecology is not sufficiently integrated into microbial selection and production [73]. Thus, Microbial inoculants tend to be chosen based on their activity in controlled laboratory screenings and for ease of mass cultivation, with minimal regard for ecologically relevant traits that will both allow them to survive in the field during a target functional period and prevent excessive persistence.

### 4.6. Limitations

While the multi-year dataset demonstrates robust, reproducible statistical associations between specific dual-microbe inoculations and improved citrus drought tolerance traits, several inherent limitations must be recognized. First, all trials were confined to pot-scale container architectures moving from controlled glasshouses to localized open orchards; hence, the spatial root distribution and macro-nutrient dynamics may vary compared to established open-field orchard soils. Second, because our focus centred on physiological and biochemical profiling, direct mechanical validation of root-to-shoot signalling dynamics—such as root tissue concentrations, xylem sap flow extraction profiles, or stomatal sensitivity thresholds—was not executed. Lastly, the downstream enzymatic and hormonal shifts documented here await validation via high-throughput molecular transcription profiling (e.g., expression validation of ABA biosynthetic marker genes like NCED). Future exploration tracing these molecular pathways under multi-site field trial layouts will be necessary to solidify commercial inoculant recommendations.

## 5. Conclusions

Arbuscular mycorrhizal fungi in association with MHB, particularly co-inoculation of *G*. *intraradices* and PSB ameliorated the morpho-physiological, biochemical and soil biological attributes of citrus seedlings, Troyer citrange and Cleopatra mandarin, under low moisture stress. These results imply that dual inoculation significantly alters leaf tissue ABA concentration and mitigates downstream vegetative moisture degradation under experimental drought. Thus, because of this synergistic effect, microbial communities might be better able to modify their metabolic processes and strategies for allocating water resources, which would improve absorption by plant roots. These results implied that, especially in light of climate change, a variety of microbial communities with intricate relationships could lessen the detrimental impacts of environmental stress on soil function. Our research contributed to the development of sustainable agriculture in the face of growing worldwide water deficiency stress concerns by providing new insights into sustainable soil-plant management through beneficial microorganisms. Based on the monitored water relations and carbon assimilation matrices, Troyer citrange rootstock demonstrated a more functional physiological buffer profile under water deficit stress than Cleopatra mandarin. To gain more understanding of the function of mycorrhizas in plant evolution, future studies of this and related working hypotheses under climate change scenarios are required. In vitro inoculation of AMF that have a synergistic coordination with particular rhizobacteria could be done in the context of future thrusts and contrasted with inorganic chemical fertilizers. Additionally, future study may focus on identifying the genes that mycorrhizal fungus upregulates and/or downregulates under water shortage stress by controlling water/nutrient intake, osmotic adjustment, hormonal signalling and antioxidant defences.

## Supporting information

S1 File Data of the effect of microbial inoculants on drought-associated physiological traits of citrus seedlings under differential soil moisture regimes. (XLSX)

## References

[1] Song F, Pan Z, Bai F, An J, Liu J, Guo W, et al. The Scion/Rootstock Genotypes and Habitats Affect Arbuscular Mycorrhizal Fungal Community in Citrus. Front Microbiol. 2015;6:1372. doi: 10.3389/fmicb.2015.01372 26648932 PMC4664953

[2] Li J, Zhou L, Chen G, Yao M, Liu Z, Li X, et al. Arbuscular mycorrhizal fungi enhance drought resistance and alter microbial communities in maize rhizosphere soil. Environmental Technology & Innovation. 2025;37:103947. doi: 10.1016/j.eti.2024.103947

[3] Augé RM, Stodola AJ, Ebel RC, Duan XR. Leaf elongation and water relations of mycorrhizal sorghum in response to partial soil drying: two *Glomus* species at varying phosphorus fertilization. J Exp Bot. 1995;46:297–307.

[4] Davies WJ, Tardieu F, Trejo CL. How Do Chemical Signals Work in Plants that Grow in Drying Soil?. Plant Physiol. 1994;104(2):309–14. doi: 10.1104/pp.104.2.309 12232081 PMC159200

[5] Jones RJ, Mansfield TA. Suppression of stomatal opening in leaves treated with abscisic acid. J Exp Bot. 1970;21:714–9.

[6] Allen MF, Moore Jr. TS, Christensen M. Phytohormone changes in *Bouteloua gracilis* infected by vesicular–arbuscular mycorrhizae. II. Altered levels of gibberellin-like substances and abscisic acid in the host plant. Can J Bot. 1982;60(4):468–71. doi: 10.1139/b82-063

[7] Duan X, Neuman DS, Reiber JM, Green CD, Saxton AM, Augé RM. Mycorrhizal influence on hydraulic and hormonal factors implicated in the control of stomatal conductance during drought. J Exp Bot. 1996;47(10):1541–50. doi: 10.1093/jxb/47.10.1541

[8] Goicoechea N, Antolin MC, Sanchez–Diaz M. Gas exchange is related to the hormone balance in mycorrhizal or nitrogen–fixing alfalfa subjected to drought. Physiol Plant. 1997;100:989–97.

[9] Bahadur A, Batool A, Nasir F, Jiang S, Mingsen Q, Zhang Q, et al. Mechanistic Insights into Arbuscular Mycorrhizal Fungi-Mediated Drought Stress Tolerance in Plants. Int J Mol Sci. 2019;20(17):4199. doi: 10.3390/ijms20174199 31461957 PMC6747277

[10] Nasslahsen B, Prin Y, Ferhout H, Smouni A, Duponnois R. Mycorrhizae helper bacteria for managing the mycorrhizal soil infectivity. Front Soil Sci. 2022;2. doi: 10.3389/fsoil.2022.979246

[11] Panneerselvam P, Saritha B, Mohandas S, Upreti KK, Poovarasan S, Sulladmath VV, et al. Effect of mycorrhiza-associated bacteria on enhancing colonization and sporulation of *Glomus* *mosseae* and growth promotion in sapota (*Manilkara achras* (Mill) Forsberg) seedlings. Biological Agriculture & Horticulture. 2013;29(2):118–31. doi: 10.1080/01448765.2013.779076

[12] Phillips JM, Hayman DS. Improved procedure for clearing roots and staining parasitic and vesicular–arbuscular mycorrhizal fungi for rapid assessment of infection. Trans Br Mycol Soc. 1970;55:158–61.

[13] Barrs H, Weatherley P. A Re-Examination of the Relative Turgidity Technique for Estimating Water Deficits in Leaves. Australian Journal of Biological Sciences. 1962;15(3):413–28. doi: 10.1071/bi9620413

[14] Hiscox JD, Israelstam GF. A method for the extraction of chlorophyll from leaf tissue without maceration. Can J Bot. 1979;57(12):1332–4. doi: 10.1139/b79-163

[15] Bates LS, Waldren RP, Teare ID. Rapid determination of free proline for water-stress studies. Plant Soil. 1973;39(1):205–7. doi: 10.1007/bf00018060

[16] Malik CP, Singh MB. In: Plant Enzymology and Histoenzymology. Kalyani Publishers: New Delhi. 1980. pp. 53.

[17] Buysse J, Merckx R. An Improved Colorimetric Method to Quantify Sugar Content of Plant Tissue. J Exp Bot. 1993;44(10):1627–9. doi: 10.1093/jxb/44.10.1627

[18] Mukherjee SP, Choudhuri MA. Implications of water–stress–induced changes in the levels of endogenous ascorbic acid and hydrogen peroxide in *Vigna* seedlings. Physiol Plant. 1983;58:166–70.

[19] Grifith OW. Glutathione and glutathione disulphide. In: Bergmeyer HU, editor. Methods of enzymatic analysis. Weinheim: Verlagsgesellschaft mbH. 1985. p. 521–9.

[20] Smith IK. Stimulation of glutathione synthesis in photorespiring plants by catalase inhibitors. Plant Physiol. 1985;79(4):1044–7. doi: 10.1104/pp.79.4.1044 16664526 PMC1075023

[21] Ali K, Gujjar RS, Niwas R, Gopal M, Tyagi A. A Rapid Method for Estimation of Abscisic Acid and Characterization of ABA Regulated Gene in Response to Water Deficit Stress from Rice. American J of Plant Physiology. 2011;6(3):144–56. doi: 10.3923/ajpp.2011.144.156

[22] Mayo K, Davis RE, Motta J. Stimulation of Germination of Spores of *Glomus versiforme* by Spore-Associated Bacteria. Mycologia. 1986;78(3):426–31. doi: 10.1080/00275514.1986.12025265

[23] Budi SW, van Tuinen D, Martinotti G, Gianinazzi S. Isolation from the Sorghum bicolor mycorrhizosphere of a bacterium compatible with arbuscular mycorrhiza development and antagonistic towards soilborne fungal pathogens. Appl Environ Microbiol. 1999;65(11):5148–50. doi: 10.1128/AEM.65.11.5148-5150.1999 10543835 PMC91693

[24] Sannazzaro AI, Alberto E, Ruiz OA, Menendez B. Influence of the arbuscular mycorrhizal fungus *Glomus intraradices* on the saline stress physiology of Lotus glaber. Lotus Newsletter. 2005;35:29–30.

[25] Camprubí A, Calvet C. Isolation and Screening of Mycorrhizal Fungi from Citrus Nurseries and Orchards and Inoculation Studies. HortSci. 1996;31(3):366–9. doi: 10.21273/hortsci.31.3.366

[26] Barman P, Singh SK, Patel VB, Singh AK, Nain L. Synergistic interaction of arbuscular mycorrhizal fungi and mycorrhiza helper bacteria improving antioxidant activities in Troyer citrange and Cleopatra mandarin under low moisture stress. Ind Jour of Hort. 2015;72(1):33. doi: 10.5958/0974-0112.2015.00006.7

[27] de Oliveira AN, de Oliveira LA. Seasonal dynamics of arbuscular mycorrhizal fungi in plants of *Theobroma grandiflorum* Schum and *Paullinia cupana* Mart. of an agroforestry system in Central Amazonia, Amazonas State, Brazil. Braz J Microbiol. 2005;36(3):262–70. doi: 10.1590/s1517-83822005000300011

[28] Marulanda A, Barea J-M, Azcón R. Stimulation of Plant Growth and Drought Tolerance by Native Microorganisms (AM Fungi and Bacteria) from Dry Environments: Mechanisms Related to Bacterial Effectiveness. J Plant Growth Regul. 2009;28(2):115–24. doi: 10.1007/s00344-009-9079-6

[29] Bryla DR, Duniway JM. Effects of mycorrhizal infection on drought tolerance and recovery in safflower and wheat. Plant and Soil. 1997;197(1):95–103. doi: 10.1023/a:1004286704906

[30] Marulanda A, Porcel R, Barea JM, Azcón R. Drought tolerance and antioxidant activities in lavender plants colonized by native drought-tolerant or drought-sensitive *Glomus* Species. Microb Ecol. 2007;54(3):543–52. doi: 10.1007/s00248-007-9237-y 17431706

[31] Augé RM. Water relations, drought and vesicular-arbuscular mycorrhizal symbiosis. Mycorrhiza. 2001;11(1):3–42. doi: 10.1007/s005720100097

[32] Subramanian KS, Charest C. Nutritional, growth, and reproductive responses of maize (*Zea mays* L.) to arbuscular mycorrhizal inoculation during and after drought stress at tasselling. Mycorrhiza. 1997;7(1):25–32. doi: 10.1007/s005720050159

[33] El–Tohamy W, Schnitzler WH, El–Behairy U. Effect of VA mycorrhiza on improving drought and chilling tolerance of bean plants. J Appl Bot. 1999;73:178–83.

[34] Tripathi A, Srivastava NK, Khan KK. Effect of vesicular arbuscular mycorrhizal fungi on papaya (*Carica papaya* L.) plant under water–stress conditions. Indian Journal of Scientific Research. 2011;2(4):65–7.

[35] Morade AS, Sharma RM, Dubey AK, Sathee L, Kumar S, Kadam DM, et al. Phenotyping drought stress tolerance in citrus rootstocks using high-throughput imaging and physio-biochemical techniques. BMC Plant Biol. 2025;25(1):753. doi: 10.1186/s12870-025-06823-0 40468193 PMC12135332

[36] Pandino G, Abbate C, Scavo A, Di Benedetto D, Mauromicale G, Lombardo S. Co-inoculation of arbuscular mycorrhizal fungi and plant growth promoting bacteria improves plant growth and yield of globe artichoke (*Cynara cardunculus* scolymus). Scientia Horticulturae. 2024;335:113365. doi: 10.1016/j.scienta.2024.113365

[37] Llosá MJ, Bermejo A, Almudena A, Quiñones A, Forner-Giner MA. The Citrus Rootstocks Cleopatra Mandarin, *Poncirus trifoliata*, Forner-Alcaide 5 and Forner-Alcaide 13 Vary in Susceptibility to Iron Deficiency Chlorosis. jpom. 2009;63(4):160–7. doi: 10.71318/apom.2009.63.4.160

[38] Streb P, Feierabend J. Oxidative Stress Responses Accompanying Photoinactivation of Catalase in NaCI‐treated Rye Leaves. Botanica Acta. 1996;109(2):125–32. doi: 10.1111/j.1438-8677.1996.tb00552.x

[39] Wu Q-S, Xia R-X. Arbuscular mycorrhizal fungi influence growth, osmotic adjustment and photosynthesis of citrus under well-watered and water stress conditions. J Plant Physiol. 2006;163(4):417–25. doi: 10.1016/j.jplph.2005.04.024 16455355

[40] Porcel R, Ruiz-Lozano JM. Arbuscular mycorrhizal influence on leaf water potential, solute accumulation, and oxidative stress in soybean plants subjected to drought stress. J Exp Bot. 2004;55(403):1743–50. doi: 10.1093/jxb/erh188 15208335

[41] Morte A, Diaz G, Rodriguez P, Alarcon JJ, Sanchez–Blanco MJ. Growth and water relations in mycorrhizal and nonmycorrhizal *Pinus* *halepensis* plants in response to drought. Biol Plant. 2001;44(2):263–7.

[42] Sánchez-Blanco MJ, Ferrández T, Morales MA, Morte A, Alarcón JJ. Variations in water status, gas exchange, and growth in *Rosmarinus* *officinalis* plants infected with *Glomus* *deserticola* under drought conditions. J Plant Physiol. 2004;161(6):675–82. doi: 10.1078/0176-1617-01191 15266714

[43] Beltrano J, Ronco MG. Improved tolerance of wheat plants (*Triticum aestivum* L.) to drought stress and rewatering by the arbuscular mycorrhizal fungus *Glomus claroideum*: effect on growth and cell membrane stability. Braz J Plant Physiol. 2008;20(1):29–37. doi: 10.1590/s1677-04202008000100004

[44] Mathur N, Vyas A. Influence of arbuscular mycorrhizae on biomass production, nutrient uptake and physiological changes in *Ziziphus* *mauritiana* Lam. under water stress. Journal of Arid Environments. 2000;45(3):191–5. doi: 10.1006/jare.2000.0644

[45] Khoshbakht D, Ramin AA, Baninasab B. Citrus Rootstocks Response to Salinity: Physio-biochemical Parameters Changes. Research J of Environmental Sciences. 2014;8(1):29–38. doi: 10.3923/rjes.2014.29.38

[46] Davies FT, Potter JR, Linderman RG. Drought resistance of mycorrhizal pepper plants independent of leaf P–concentration– response in gas exchange and water relations. Physiol Plant. 1993;87:45–53.

[47] Bárzana G, Aroca R, Paz JA, Chaumont F, Martinez-Ballesta MC, Carvajal M, et al. Arbuscular mycorrhizal symbiosis increases relative apoplastic water flow in roots of the host plant under both well-watered and drought stress conditions. Ann Bot. 2012;109(5):1009–17. doi: 10.1093/aob/mcs007 22294476 PMC3310489

[48] Sheng M, Tang M, Chen H, Yang B, Zhang F, Huang Y. Influence of arbuscular mycorrhizae on photosynthesis and water status of maize plants under salt stress. Mycorrhiza. 2008;18(6–7):287–96. doi: 10.1007/s00572-008-0180-7 18584217

[49] Gale J, Zeroni M. The cost to plants of different strategies of adaptation to stress and the alleviation of stress by increasing assimilation. Plant Soil. 1985;89(1–3):57–67. doi: 10.1007/bf02182233

[50] Mathur N, Vyas A. I. Influence of VA Mycorrhizae on Net Photosynthesis and Transpiration of *Ziziphus mauritiana*. Journal of Plant Physiology. 1995;147(3–4):328–30. doi: 10.1016/s0176-1617(11)82161-9

[51] Gemma JN, Koske RE, Roberts EM. Mycorrhizal fungi improve drought resistance in creeping bentgrass. J Turfgrass Sci. 1997;73:15–29.

[52] Nikolaou N, Angelopoulos K, Karagiannidis N. Effects of drought stress on mycorrhizal and non-mycorrhizal Cabernet Sauvignon grapevine, grafted onto various rootstocks. Ex Agric. 2003;39(3):241–52. doi: 10.1017/s001447970300125x

[53] Lehto T, Zwiazek JJ. Ectomycorrhizas and water relations of trees: a review. Mycorrhiza. 2011;21(2):71–90. doi: 10.1007/s00572-010-0348-9 21140277

[54] Morade AS, Sharma RM, Dubey AK, Sathee L, Kadam DM, Mahawer AK, et al. Drought-induced modulations in stomatal traits and potential conductance index of citrus rootstock genotypes. Plant Physiol Rep. 2026;31(1):83–97. doi: 10.1007/s40502-025-00920-w

[55] Vivas A, Marulanda A, Ruiz-Lozano JM, Barea JM, Azcón R. Influence of a *Bacillus* sp. on physiological activities of two arbuscular mycorrhizal fungi and on plant responses to PEG-induced drought stress. Mycorrhiza. 2003;13(5):249–56. doi: 10.1007/s00572-003-0223-z 14593518

[56] Eftekhari M, Alizadeh M, Mashayekhi K, Asghari H, Kamkar B. Integration of arbuscular mycorrhizal fungi to grape vine (*Vitis vinifera* L.) in nursery stage. Journal of Advanced Laboratory Research in Biology. 2010;1(2):103–11.

[57] Tang M, Chen H, Shang HS. Mechanism of vesicular–arbuscular mycorrhizal fungi enhanced the resistance of poplar to canker. Scientia Silvae Sinica. 2000;36(2):87–92.

[58] Smirnoff N. The role of active oxygen in the response of plants to water deficit and desiccation. New Phytol. 1993;125(1):27–58. doi: 10.1111/j.1469-8137.1993.tb03863.x 33874604

[59] Kandowangko NY, Suryatmana G, Nurlaeny N, Simanungkalit RDM. Proline and Abscisic Acid Content in Droughted Corn Plant Inoculated with *Azospirillum* sp. and Arbuscular Mycorrhizae Fungi. HAYATI Journal of Biosciences. 2009;16(1):15–20. doi: 10.4308/hjb.16.1.15

[60] Ruscitti M, Arango M, Ronco M, Beltrano J. Inoculation with mycorrhizal fungi modifies proline metabolism and increases chromium tolerance in pepper plants (*Capsicum annuum* L.). Braz J Plant Physiol. 2011;23(1):15–25. doi: 10.1590/s1677-04202011000100004

[61] Amira SS, Nermeen TS, Osama NM, D M S. Improving salinity tolerance of *Acacia saligna* (Labill.) plant by arbuscular mycorrhizal fungi and *Rhizobium* inoculation. Afr J Biotechnol. 2012;11(5):1259–66. doi: 10.5897/ajb11.2287

[62] Wu Q-S, Xia R-X, Zou Y-N, Wang G-Y. Osmotic solute responses of mycorrhizal citrus (*Poncirus trifoliata*) seedlings to drought stress. Acta Physiol Plant. 2007;29(6):543–9. doi: 10.1007/s11738-007-0065-y

[63] Abbaspour H, Saeidi-Sar S, Afshari H, Abdel-Wahhab MA. Tolerance of Mycorrhiza infected pistachio (*Pistacia vera* L.) seedling to drought stress under glasshouse conditions. J Plant Physiol. 2012;169(7):704–9. doi: 10.1016/j.jplph.2012.01.014 22418429

[64] Martìnez J-P, Lutts S, Schanck A, Bajji M, Kinet J-M. Is osmotic adjustment required for water stress resistance in the Mediterranean shrub *Atriplex halimus* L?. J Plant Physiol. 2004;161(9):1041–51. doi: 10.1016/j.jplph.2003.12.009 15499906

[65] Devlin MR, Withman FH. Plant Physiology. Delhi, India: GBS publishers and distributors. 2002.

[66] Sultana N, Ikeda T, Itoh R. Effect of NaCl salinity on photosynthesis and dry matter accumulation in developing rice grains. Environmental and Experimental Botany. 1999;42(3):211–20. doi: 10.1016/s0098-8472(99)00035-0

[67] Enteshari S, Hajbagheri S. Effects of mycorrhizal fungi on photosynthetic pigments, root mycorrhizal colonization and morphological characteristics of salt stressed *Ocimum basilicum* L. Iranian Journal of Plant Physiology. 2011;1(4):215–22.

[68] Zandalinas SI, Balfagón D, Arbona V, Gómez-Cadenas A. Modulation of Antioxidant Defense System Is Associated with Combined Drought and Heat Stress Tolerance in Citrus. Front Plant Sci. 2017;8:953. doi: 10.3389/fpls.2017.00953 28638395 PMC5461256

[69] Wu QS, Zou YN, Xia RX. Effects of water-stress and arbuscular mycorrhizal fungi on reactive oxygen metabolism and antioxidant production by citrus (*Citrus tangerine*) roots. European Journal of Soil Biology. 2006;42:166–72.

[70] Zandalinas SI, Rivero RM, Martínez V, Gómez-Cadenas A, Arbona V. Tolerance of citrus plants to the combination of high temperatures and drought is associated to the increase in transpiration modulated by a reduction in abscisic acid levels. BMC Plant Biol. 2016;16:105. doi: 10.1186/s12870-016-0791-7 27121193 PMC4848825

[71] Begum N, Qin C, Ahanger MA, Raza S, Khan MI, Ashraf M, et al. Role of Arbuscular Mycorrhizal Fungi in Plant Growth Regulation: Implications in Abiotic Stress Tolerance. Front Plant Sci. 2019;10:1068. doi: 10.3389/fpls.2019.01068 31608075 PMC6761482

[72] Correia TS, Lara TS, Santos JAD, Sousa L da S, Santana MDF. Arbuscular Mycorrhizal Fungi Promote Physiological and Biochemical Advantages in *Handroanthus serratifolius* Seedlings Submitted to Different Water Deficits. Plants (Basel). 2022;11(20):2731. doi: 10.3390/plants11202731 36297755 PMC9609855

[73] Kaminsky LM, Trexler RV, Malik RJ, Hockett KL, Bell TH. The Inherent Conflicts in Developing Soil Microbial Inoculants. Trends Biotechnol. 2019;37(2):140–51. doi: 10.1016/j.tibtech.2018.11.011 30587413

