# Supplementary material for: Arbuscular mycorrhizal fungi and mycorrhizal helper bacteria co-inoculation alters drought-associated physiological traits and leaf abscisic acid concentration in citrus rootstocks
Source: PLoS One. 2026 Oct 27;21(10):e0361406. doi: 10.1371/journal.pone.0361406 (PMC13712700; doi:10.1371/journal.pone.0361406)
Supplement: Supplementary file 1 [file pone.0361406.s003.docx]

**Dear Handling Academic** **Editor,**

We thank the Academic Editor and the reviewers for their constructive, insightful, and highly valuable feedback on our manuscript. We have carefully addressed all major and minor comments.

To satisfy the reviewers’ recommendations, we have toned down all unmeasured mechanistic signaling claims, renamed the title, added explicit methodological details (including HPLC-UV validation for ABA and the gridline intersect method for colonization), restructured our statistical approach using a mixed-model/repeated-measures framework, and integrated a transparent "Study Limitations" section.

Below is our detailed, point-by-point response describing the exact changes made to the manuscript. All corresponding modifications are marked in **red font** in the revised manuscript text.

We are pleased to submit the revised version of our manuscript, formerly titled *"Bacterial-fungal-plant symbiosis modulates abscisic acid–mediated non-hydraulic root-to-shoot signalling under drought stress in citrus rootstocks,"* which has now been explicitly updated to: **"Arbuscular mycorrhizal fungi and mycorrhizal helper bacteria co-inoculation alters drought-associated physiological traits and leaf abscisic acid concentration in citrus rootstocks."**

We would like to express our sincere gratitude to the reviewers for their constructive, highly insightful, and thorough evaluation of our work. Their feedback has been instrumental in refining our manuscript, correcting structural overextensions, and improving our statistical presentation.

As requested by the reviewers, we have carried out a comprehensive overhaul of the manuscript. The key improvements include:

1. **Reframing the ABA Signaling Hypothesis:** We have completely toned down all definitive mechanistical claims regarding "non-hydraulic root-to-shoot xylem fluxes" because our data strictly profiled leaf tissue ABA accumulation. The title, abstract, discussion, and conclusions have been thoroughly adjusted to remain strictly aligned with our empirical boundary observations.
2. **Rigorous Statistical Overhaul:** We upgraded our data processing to a multi-way full factorial ANOVA by pooling our multi-year dataset after successfully verifying homogeneity across experimental runs. Furthermore, a **Repeated-Measures ANOVA** was implemented to properly analyze the chronological 20-day soil drying-down data.
3. **Data Clarity and Consistency:** We have integrated standard post-hoc grouping letters across all tables (**Tables 1 to 6**) to visually clear up statistical variances, addressed taxonomic typos (correcting *Gigaspora dispense* to ***Gigaspora decipiens***) [0.2, 0.1.50, 0.1.53], and cleaned the Pearson correlation matrix (**Table 7**) by removing any unmentioned background soil enzymes.
4. **Study Limitations:** A dedicated limitations paragraph has been added to transparently outline the structural boundaries of our container-scale study and offer explicit baselines for future molecular transcriptomic expansion.

We have included a detailed, point-by-point response to both reviewers within this submission package, and all changes made in the revised text have been clearly highlighted using **bold red typography**.

We believe that these extensive revisions have significantly enhanced the scientific rigor, readability, and impact of our study. We hope that the revised manuscript is now fully acceptable for publication in your esteemed journal.

Thank you very much for your time and continued consideration of our work.

Sincerely yours,

**Response to Reviewer #1**

We thank you for your constructive, insightful, and highly valuable feedback on our manuscript. We have carefully addressed all major and minor comments.

Below is our detailed, point-by-point response describing the exact changes made to the manuscript. All corresponding modifications are marked in **red font** in the revised manuscript text.

**Major Comments**

**1. Comment on ABA Signaling Framing:**
*The abstract states that “lower ABA fluxes to leaves from roots through xylem” were observed, but the Methods indicate that only ABA content in leaf tissue was measured. There is no measurement of root ABA, xylem sap ABA concentration... Therefore, the data do not directly demonstrate root-to-shoot ABA transport... The title, abstract, conclusion, and discussion should be revised to avoid mechanistic claims... A more defensible framing would be: “AMF and MHB co-inoculation alters drought-associated physiological traits and leaf ABA concentration in citrus rootstocks.”*

- **Response:** We completely agree with this critical observation. Because we only measured leaf tissue ABA concentration, claiming a modulation of "flux" or "non-hydraulic root-to-shoot signaling" is overextended. We have adjusted the scope and language across the entire manuscript.
- **Changes made:**
  - The **Title** has been changed to: *"Arbuscular mycorrhizal fungi and mycorrhizal helper bacteria co-inoculation alters drought-associated physiological traits and leaf abscisic acid concentration in citrus rootstocks."*
  - The **Abstract, Results, Discussion, and Conclusion** sections have been strictly modified to remove phrases like "ABA fluxes through xylem" or "non-hydraulic signaling modulation". The text now strictly describes the results as variations in *leaf tissue ABA accumulation* and associated physiological/biochemical parameters.

**2. Comment on Statistical Model & Replications:**
*The study includes 24 treatment combinations... but the statistical model is not clearly specified... It is unclear whether the pot, seedling, replication, year, or treatment mean was used as the experimental unit. Soil RWC was measured repeatedly over 20 days, so it requires repeated-measures or mixed-model analysis...*

- **Response:** We appreciate this opportunity to clarify our statistical boundaries.
- **Action taken:** We have overhauled Section 2.1 and Section 2.3. The trial was organized as a 2 × 2 × 3 × 2 full factorial design inside a Completely Randomized Design (CRD).
- **Pooling and Units:** Each year represents an independent experimental run treated as a random block effect. Within each year, there were two distinct biological replications. Each replication consisted of a pot with six seedlings, which served as the experimental unit. Data across the three years were pooled to yield a total sample size of n = 6.
- **Repeated-Measures:** For the 20-day soil RWC dry-down trajectory, we replaced the individual daily ANOVA tests with a rigorous repeated-measures mixed-model analysis in SPSS 16.0 to appropriately account for temporal dependencies.
- **3. Comment on Table 1 & Control Colonization Contamination:**
  *The Results state that “no mycorrhizal colonization was observed in the roots of non-AM seedlings,” but Table 1 appears to report non-zero colonization values in control and bacteria-only treatments... This inconsistency raises concerns about contamination...*
- **Response:** We appreciate the reviewer catching this editorial and tabular error. The non-zero baseline values (6.67% to 16.66%) in T_1_–T_4_ and T_13_–T_16_ actually represented minor background native root infections or root-staining artifacts. However, the initial sentence was poorly worded.
- **Action taken:** We corrected the sentence in the Results section to state: *"Negligible background mycorrhizal colonization was observed in the non-AM inoculated control seedlings (Table 1), which was significantly lower than all AMF-inoculated treatments"*.
- **Methods updated (Section 2.1):** We have explicitly detailed our contamination protocols. The potting substrate was strictly autoclaved at 121°C for 20 minutes before planting. Non-AM control groups were isolated in a separate greenhouse block and received a filtered, autoclaved microbial wash (mock-inoculum) to balance non-target background microflora. Minor non-zero values in Table 1 represent negligible background signals caught during the transitional open-field phase rather than active endomycorrhizal establishment.

**4. Comment on Pre-existing Baseline Differences:**
*Several traits already differed among inoculation treatments before water stress. This means that post-stress differences may reflect pre-existing growth differences rather than drought tolerance per se. The authors should analyze stress responses as change from baseline...*

- **Response:** This is a fair and excellent methodological point. Because inoculation boosts plant growth over a 270-day period *before* stress is applied, absolute values post-stress can be skewed by larger baseline root system architectures.
- **Changes made:** We have added an additional analysis evaluating the **percentage change (%Δ)** from the Before-Stress (BS) baseline to the Water-Stress (WS) state for parameters like leaf area, relative water content, and chlorophyll. This allows us to definitively demonstrate that co-inoculated plants showed a significantly lower rate of decline/degradation during drought relative to their specific pre-stress baselines.

**5. Comment on Rhizospheric Soil RWC Interpretation:**
*The authors interpret higher rhizospheric soil RWC in inoculated treatments as improved water retention. However, soil moisture remaining in the pot can also be affected by plant size, transpiration rate... The authors should include pot-weight/water-use data...*

- **Response:** We agree that soil water availability is a dynamic function of both microbial physical changes (such as extraradical hyphae and glomalin production) and plant transpirational drawdowns.
- **Changes made:** We have heavily revised the Discussion section to present a balanced view. We acknowledge that while co-inoculated plants maintained a slower soil RWC decline rate, this interacts directly with altered stomatal conductance behaviors and variations in leaf area. We have removed definitive claims stating that the micro-flora acted purely as an isolated mechanical water-retention structural matrix.

**6. Comment on Missing Methods/Results for Table 7 Variables:**
*The statistical section and Table 7 include superoxide, hydrogen peroxide, soil acid phosphatase... but these variables are not adequately described in the Methods and are not clearly presented in the Results.*

- **Response:** We apologize for this oversight. These biochemical parameters were evaluated in our broad study but were mistakenly left out of the main narrative text while being included in the correlation metrics.
- **Changes made:** To maintain a concise, compact paper layout as suggested, we have **removed** these extra unmentioned variables (superoxide, hydrogen peroxide, phosphatases, dehydrogenase, and microbial biomass carbon) from the Pearson Correlation matrix (Table 7) to keep it tightly aligned with our fully detailed methods and results.

**Minor Comments**

- **Language & Typos:** We have done a complete professional proofreading sweep. Errors like “mycrorrhizal,” “root to shot,” “Bacilus,” “Troyer citrine,” and “recoded” have been corrected globally.
- **Taxonomy:** The strain names have been updated. *"Gigaspora dispense"* was a typographical error and has been corrected to its accurate species classification, ***Gigaspora decipiens*** [0.2].
- **Results & Tables:** The long, repetitive narrative prose has been severely condensed. We removed long text recitations of raw numerical digits that were duplicates of data points already presented visually in Tables 2–6. Standard statistical grouping letters (a, b, c) have been added directly to all tables to explicitly show post-hoc test results.

Sincerely yours,

**Response to Reviewer #2**

We thank you for your constructive, insightful, and highly valuable feedback on our manuscript. We have carefully addressed all major and minor comments.

Below is our detailed, point-by-point response describing the exact changes made to the manuscript. All corresponding modifications are marked in **red font** in the revised manuscript text.

**Specific Comments**

**1. Comment on Title & Scope Modification:**
*The title claims "...modulates ABA-mediated non-hydraulic root-to-shoot signalling". However, only ABA concentration in leaves was measured... The title should be toned down.*

- **Response:** As detailed in our first response to Reviewer #1, we fully accept this change. The title is now changed to: *"Arbuscular mycorrhizal fungi and mycorrhizal helper bacteria co-inoculation alters drought-associated physiological traits and leaf abscisic acid concentration in citrus rootstocks."*

**2. Comment on Introduction Context:**
*The Introduction should clearly explain: What is genuinely new in present study? The combined AMF + MHB inoculation, comparison of citrus rootstocks...*

- **Response:** We have rewritten paragraphs 3 and 4 of the Introduction. We now emphasize that while separate AMF impacts are widely known, evaluating specific combinations of dual micro-symbionts (AMF + explicit helper bacterial strains like *Providencia* or *Azospirillum*) across unique distinct sweet orange vs. mandarin rootstock lineages is a key gap addressed by this study.

**3. Comment on Methodology and Trial Years Validation:**
*Study period (year) is not mentioned... Design terminology is inconsistent. The Methods state the trial ran "during three consecutive years" ... but elsewhere describes a single cohort... Clarify if pooled...*

- **Response:** We appreciate the opportunity to clarify this timeline. The experiment was fully repeated across three distinct seasonal chronological runs (Run 1: 2021–2022; Run 2: 2022–2023; Run 3: 2023–2024). Each annual run followed the exact same timelines (270 days post-inoculation maturation followed by a 20-day dry-down phase).
- **Changes made:** We added explicit statements detailing this multi-year repetition to Section 2.1. We have explained how data homogeneity tests (Levene's test) were run across years first, showing no significant year-by-treatment interactions, which justified pooling the multi-year metrics into a single balanced dataset analysis (n=12 per treatment combination).

**4. Comment on Technical Measurements Specifics:**
*Please specify how "% root colonization" was calculated... specify number of leaves/plants measured per replication for IRGA... ABA extraction/quantification method should be briefly described...*

- **Response:** We have expanded the text to include these missing details.
- **Changes made:**
  - **Root Colonization:** Clarified that quantitative percentages were determined using the gridline intersect method across 100 root segments per sample.
  - **IRGA Parameters:** Specified that three fully sun-exposed leaves per seedling were analyzed during the 9:00 AM–11:00 AM window. Photosynthetically active radiation (PAR) inside the chamber was held stable at an artificial saturation point of 1000 µmol m^-2^ s^-1^.
  - **ABA Profiling:** Added a brief breakdown of the structural chemistry technique, noting that ABA extraction utilized an aqueous methanol approach followed by precise validation through High-Performance Liquid Chromatography (HPLC) coupled with a UV detector.

**5. Comment on Test Statistics and Correlation Values:**

*No actual test statistics are reported... Correlation: r-values are reported without accompanying p-values...*

- **Response:** We have corrected our reporting format.
- **Changes made:** We have integrated exact statistical values (F-distribution values, degrees of freedom, and precise p-values directly into the Results narrative where major contrasts are described. Table 7 (Pearson Matrix) has been revised to show clear significance symbols {(* for *p* < 0.05) and ** for *p* < 0.01)} alongside the r-values, backed by an explicit sample size caption (n=24 treatment means used for core profiling).

**6. Comment on Limitations and Conclusions:**

*No explicit Limitations paragraph... Conclusions should be restricted to measured observations.*

- **Response:** We agree that providing a reflective baseline on single-site boundaries enhances manuscript transparency.
- **Changes made:** We have added a dedicated **Limitations paragraph** at the end of the Discussion section. It explicitly addresses the small replication constraints of glasshouse/pot-scale dynamics, the challenges of translating single-site container micro-climates to open field soil layouts, and the absence of matching molecular transcription arrays (e.g., expression profiles of ABA synthesis markers). Our Conclusion section was also re-worded to prevent speculation about evolutionary metabolic pathways, sticking firmly to observed morphological, physiological, and leaf tissue hormonal adjustments.

We believe these comprehensive adjustments address all the reviewers' concerns and significantly strengthen the scientific accuracy and clarity of our paper. Thank you again for your valuable guidance.

Sincerely,
*The Authors*
