# Supplementary material for: Arbuscular mycorrhizal fungi and mycorrhizal helper bacteria co-inoculation alters drought-associated physiological traits and leaf abscisic acid concentration in citrus rootstocks
Source: PLoS One. 2026 Oct 27;21(10):e0361406. doi: 10.1371/journal.pone.0361406 (PMC13712700; doi:10.1371/journal.pone.0361406)
Supplement: Supplementary file 2 [file pone.0361406.s004.docx]

**Dear Handling Academic** **Editor,**

We sincerely thank the Editor and the Reviewers for their careful evaluation and constructive comments. We have carefully considered all comments and incorporated the necessary corrections throughout the revised manuscript. **For clarity, all the corrections made in the manuscript as responses to Reviewer #1 are shown in RED font and responses to Reviewer #2 and BLUE font**.

Regards

**Response to Reviewer #1**

We thank you for your constructive, insightful, and highly valuable feedback on our manuscript. We have carefully addressed all major and minor comments. Below is our detailed, point-by-point response describing the exact changes made to the manuscript. All corresponding modifications are marked in **RED font** in the revised manuscript text.

**Comment 1: The title and parts of the abstract have been improved, but language related to non-hydraulic root-to-shoot signalling remains. The study did not directly measure root ABA concentration, xylem sap ABA concentration, xylem sap flow, ABA transport, or stomatal sensitivity to ABA.**

**Response:** We sincerely thank the Reviewer for this important clarification. We agree that the present study did not directly measure these components of ABA transport or signalling. Accordingly, all necessary corrections have been incorporated throughout the manuscript. Statements implying a demonstrated ABA-mediated non-hydraulic root-to-shoot signalling mechanism have been removed or appropriately toned down. The findings are now described primarily in terms of drought-associated physiological and biochemical responses and leaf ABA accumulation. Mechanistic interpretations concerning ABA-mediated root-to-shoot signalling are presented only as a possible hypothesis for future investigation.

**Comment 2: The statistical analysis requires clarification because the manuscript states n = 6 after pooling across years, whereas the response letter mentions n = 12.**

**Response:** We thank the Reviewer for identifying this inconsistency. The discrepancy has been corrected throughout the manuscript and response letter. The correct number of independent biological replications is n = 6, not n = 12. The pot was considered the experimental unit, with six seedlings maintained per pot. The statistical analyses were based on six independent biological replicates. All statements referring to n = 12 have been corrected to n = 6.

**Comment 3: Methodological details concerning HPLC-UV validation for ABA measurement and the gridline-intersect method for AMF colonization should be clarified in the main Methods section.**

**Response:** We appreciate this suggestion. The Methods section has been revised to provide the necessary practical details for reproducibility. The ABA analysis now includes the relevant extraction and HPLC-UV procedures, calibration/standard information and validation details. The AMF colonization procedure has also been clarified, including the number of root segments examined, application of the gridline-intersect method and calculation of percentage root colonization.

**Comment 4: Concerns remain regarding controls and possible background colonization. The manuscript should describe substrate sterilization, mock inoculation, separation of controls and measures used to minimize contamination.**

**Response:** We thank the Reviewer for this important point. The Methods and Results sections have been revised to describe substrate sterilization, control/mock inoculation procedures, separation of control plants and measures taken to minimize cross-contamination during the experimental period and transition to open conditions. The wording concerning AMF colonization in non-AM treatments has also been corrected to remain consistent with the small non-zero values reported in Table 1. The statement “No mycorrhizal colonization was observed” has therefore been avoided where it could conflict with the numerical observations.

**Comment 5: Interpretation of rhizospheric soil relative water content should remain cautious and should not imply that microbial inoculation directly improved soil water retention.**

**Response:** We agree with the Reviewer. The interpretation of soil relative water content has been revised throughout the manuscript. The results are now described as an association between microbial treatment and a slower decline in soil RWC under the experimental conditions, rather than as direct evidence that microbial inoculation independently improved soil water retention. Possible contributions of plant size, leaf area, transpiration, root biomass, substrate heterogeneity and evaporation are acknowledged.

**Comment 6: The S1 File should contain the raw data underlying all tables, figures, statistical analyses and correlations.**

**Response:** We appreciate the Reviewer’s recommendation. The Supporting Information has been checked/revised to ensure that the data underlying the reported analyses are provided in an appropriate form. The data availability statement has also been revised accordingly.

**Comment 7: The English language still contains typographical and grammatical errors.**

**Response:** We thank the Reviewer for pointing this out. The manuscript has undergone careful language editing. Typographical, grammatical and terminology-related errors have been corrected throughout, including “mycrorrhizal plants,” “Bacilus,” “root to shot signalling pathway,” and “to done the study.” The manuscript has also been checked for consistency in scientific terminology and treatment nomenclature.

Regards

**Response to Reviewer #2**

We thank you for your constructive, insightful, and highly valuable feedback on our manuscript. We have carefully addressed all major and minor comments. Below is our detailed, point-by-point response describing the exact changes made to the manuscript. All corresponding modifications are marked in **Blue font** in the revised manuscript text.

**Comment 1: In Table 7, nine biologically distinct traits show very high correlations (|r| = 0.85–0.97). Please recheck the statistical analysis.**

**Response:** We thank the Reviewer for carefully examining the correlation analysis. We have rechecked the statistical analysis and the underlying calculations for Table 7. The analysis is correct and no statistical error was identified. Table 7 has therefore been retained. The correlations are based on the observations used for the correlation analysis and are reported as calculated. The interpretation has also been kept strictly correlational and does not imply causality.

**Comment 2: Letter groupings are inconsistent with the stated LSD in several tables, including Tables 2, 3 and 6.**

**Response:** We appreciate the Reviewer’s careful checking. In response, SEm and LSD values have been deleted from the tables. The statistical grouping letters have been checked and corrected wherever necessary according to the underlying analysis, and the corresponding Results text has been revised to accurately reflect the statistical groupings.

**Comment 3: For RWC, WS and WW columns show identical SEm and LSD values. Please recheck.**

**Response:** We thank the Reviewer for highlighting this issue. The RWC statistical presentation has been rechecked. In the revised tables, SEm and LSD values have been deleted as requested. The treatment comparisons and statistical grouping letters have been checked and corrected according to the underlying analysis.

**Comment 4: “At just before imposing water-stress” is awkward.**

**Response:** We agree. The wording has been corrected throughout the manuscript and is now presented as “before water-stress imposition” or “at BS,” consistent with the abbreviation convention.

**Comment 5: Several “statistically at par” claims in Section 3.9 are not supported by Table 4 groupings. Check Sections 3.15 and 3.16 as well.**

**Response:** We thank the Reviewer for this careful observation. Sections 3.9, 3.15 and 3.16 have been thoroughly rechecked against the statistical grouping letters. Statements concerning treatments being “statistically at par” have been corrected wherever necessary, and the Results text now corresponds strictly with the reported statistical groupings.

**Comment 6: Correct the statements “Troyer seedlings had higher rate of leaf respiration than Cleopatra” and “Control plants suffered total stomatal closure.”**

**Response:** We agree. These statements have been corrected to avoid unsupported generalizations. The revised Results describe the observed treatment- and condition-specific differences without making blanket statements about genotype superiority or complete stomatal closure unless directly supported by the data.

**Comment 7: The Results section should cite treatment codes such as T1 and T6 inline.**

**Response:** We appreciate this useful suggestion. Treatment codes have been incorporated throughout the Results section along with the corresponding treatment descriptions, for example, “G. intraradices + PSB (T6),” making the Results easier to trace directly to the tables.

**Comment 8: In Section 3.4, Cleopatra G. intraradices + PSB under WW should be 25.23 cm², and Cleopatra control under WW should be 15.24 cm².**

**Response:** We thank the Reviewer for identifying these errors. The values have been corrected in the revised manuscript. The corresponding Results text has also been revised.

**Comment 9: The Discussion requires substantial improvement, with actual data, percentage change, direction and statistical significance before literature comparison.**

**Response:** We agree with the Reviewer. The Discussion has been substantially revised. The revised subsections now begin with the principal findings of the present study and, where appropriate, include the direction, magnitude and statistical significance of the observed changes before comparison with previous reports.

**Comment 10: No p-value, percentage change or magnitude is reported in the Discussion.**

**Response:** We thank the Reviewer for this suggestion. The Discussion has been revised to include quantitative information wherever appropriate, including percentage changes, effect magnitudes and statistical significance based on the reported analyses.

**Comment 11: The manuscript does not discuss whether Troyer citrange and Cleopatra mandarin responded differently to inoculation.**

**Response:** We agree that genotype-dependent responses are scientifically relevant. A dedicated discussion of Troyer citrange and Cleopatra mandarin has therefore been incorporated. The revised text compares the observed response patterns between the two rootstocks while avoiding conclusions not directly supported by the statistical analysis.

**Comment 12: The ABA paragraph should be developed as a clearly identified novel interpretation.**

**Response:** We appreciate this important suggestion. The ABA interpretation has been revised and given greater prominence in the Discussion. The revised text clearly distinguishes the observed association between soil moisture status, leaf water relations and leaf ABA accumulation from a demonstrated signalling mechanism. Because root ABA concentration, xylem ABA concentration, xylem sap flow and ABA transport were not directly measured, the proposed mechanistic interpretation is explicitly presented as a hypothesis for future investigation.

**Comment 13: Break the Discussion into subheadings.**

**Response:** We agree. The Discussion has been reorganized into clear thematic subsections: 4.1 Root colonization and water relations; 4.2 Photosynthetic pigments and gas exchange; 4.3 Osmotic adjustment; 4.4 Antioxidant response; 4.5 Hormonal regulation and ABA accumulation; 4.6 Genotype-dependent responses; and 4.7 Limitations and future perspectives.

**Comment 14: Typo: “chrorophyll” should be “chlorophyll.”**

**Response:** Corrected. The spelling “chrorophyll” has been replaced with “chlorophyll” throughout the manuscript, and the manuscript has been checked for similar typographical errors.

**Comment 15: Fix bacterial treatment nomenclature throughout.**

**Response:** We thank the Reviewer for this observation. Bacterial treatment nomenclature has been standardized throughout the manuscript, including the Methods, Results, Discussion, tables, figures and Supporting Information. Treatment names and codes are now used consistently.

Regards

**General Response**

We again thank both Reviewers for their constructive comments, which have helped us improve the clarity, statistical presentation, methodological reproducibility and scientific interpretation of the manuscript. All necessary corrections have been incorporated in the revised manuscript. As requested, SEm and LSD values have been removed from the tables, the correct replication number has been stated as n = 6, and Table 7 has been retained following rechecking of the correlation analysis.

Regards
