# Supplementary material for: Arbuscular mycorrhizal fungi and mycorrhizal helper bacteria co-inoculation alters drought-associated physiological traits and leaf abscisic acid concentration in citrus rootstocks
Source: PLoS One. 2026 Oct 27;21(10):e0361406. doi: 10.1371/journal.pone.0361406 (PMC13712700; doi:10.1371/journal.pone.0361406)
Supplement: Supplementary file 3 [file pone.0361406.s005.docx]

**3rd- Revised Manuscript**

**Dear Editor,**

We express our sincere appreciation **to the Editor a**nd **Reviewers** for their constructive, analytical, and highly precise technical evaluations of our manuscript. We have systematically addressed every remaining item raised, specifically modifying descriptive vocabularies to accurately ground physiological inferences, completing chemical tracking specifications, and clarifying structural replication parameters. As a result, all ambiguities surrounding 'ABA flux,' rootstock comparisons, and independent experimental setups have been successfully corrected, providing absolute scientific alignment with our empirical datasets. Below is a detailed, point-by-point narrative of the specific improvements applied to the text.

Regards

**Reviewer #1:**

We express our sincere appreciation to Reviewer #1 for their constructive, analytical, and highly precise technical evaluations of our manuscript. We have systematically addressed every remaining item raised, specifically modifying descriptive vocabularies to accurately ground physiological inferences, completing chemical tracking specifications, and clarifying structural replication parameters. As a result, all ambiguities surrounding 'ABA flux,' rootstock comparisons, and independent experimental setups have been successfully corrected, providing absolute scientific alignment with our empirical datasets. Below is a detailed, point-by-point narrative of the specific improvements applied to the text.

**SYSTEMATIC POINT-BY-POINT REVISIONS: REVIEWER #1**

**Point 1 (Quantification Boundary & Purging 'Flux' Claims)**

**Reviewer Comment:** *First, although the authors state that all claims related to ABA-mediated root-to-shoot signalling have been removed or toned down, one problematic statement remains in the Results section. The manuscript still states that 'G. intraradices + PSB dual inoculation resulted in significantly reduced level of ABA flux into leaf from root.' This statement is not supported by the measurements performed in the study. The authors measured leaf ABA concentration, but they did not directly measure root ABA concentration, xylem sap ABA concentration, xylem sap flow, ABA transport, or ABA flux. Therefore, this sentence should be revised to refer only to leaf tissue ABA concentration. For example, the authors could write: 'G. intraradices + PSB dual inoculation resulted in significantly lower leaf tissue ABA concentration.'*

**Author Response:** The authors completely agree with this rigorous evaluation. Because direct mechanics of trans-sap transport, root core concentrations, or volumetric flow streams were not empirically monitored, the word 'flux' introduces unverified mechanistic overstatement. Accordingly, this phrase has been entirely extracted from Section 3.17 (Results) and replaced with precise vocabulary focusing strictly on our true empirical parameters—the quantified leaf tissue hormone values.

**Manuscript Text Modifications:**
Section 3.17 (Hormonal Regulation Summary) has been corrected as follows:
"Under well-watered conditions, G. intraradices + PSB dual inoculation resulted in significantly lower leaf tissue ABA concentrations in both Troyer and Cleopatra seedlings (T₆: 2.187 and T₁₈: 2.089 μg g⁻¹ F.W., respectively), which was statistically comparable to Troyer seedlings inoculated with G. intraradices + Providencia sp. (T₈: 2.672 μg g⁻¹ F.W.)."

**Point 2 (Completing HPLC-UV Methodological Reproducibility)**

**Reviewer Comment:** *Second, the methodological description of ABA quantification still requires correction. The authors added some information about extraction and HPLC-related procedures, but the description remains incomplete and partly unclear. In particular, the sentence 'Standard monitoring was performed at' appears unfinished, and the detection wavelength and several key chromatographic details are missing. The authors should provide a complete and reproducible description of the HPLC-UV method, including detection wavelength, column type, mobile phase, flow rate, calibration procedure, ABA standard, recovery correction, and how final concentrations were calculated.*

**Author Response:** We deeply regret the syntax error that left the baseline monitoring sentence broken and incomplete in the previous draft. Section 2.2 (Parameters recorded) has been extensively expanded to supply a complete, rigorous, and completely reproducible analytical description of our chromatographic parameters. This includes specific stationary phase column dimensions, isocratic mobile phase composition, precise tracking wavelengths, calibration steps, and standard matrix calculation variables.

**Manuscript Text Modifications:**
Section 2.2 (ABA Extraction & Analysis Sub-block) has been updated with the following full block:
"Chromatographic separation was achieved via an HPLC-UV system equipped with a reversed-phase C18 column (250 mm × 4.6 mm, 5 µm particle size). The mobile phase consisted of an isocratic mixture of methanol and 0.1% aqueous acetic acid (60:40, v/v) pumped at a constant flow rate of 1.0 mL/min. Standard monitoring and detection were performed at a fixed UV wavelength of 254 nm. Primary standard reference materials (≥98% purity, Sigma-Aldrich) were dissolved in chromatographically pure methanol to prepare a concentrated stock solution. Working standards were prepared via serial dilution to construct a 5-point external calibration curve. The baseline method recovery rate (R) was evaluated at 75.8 per cent. The final absolute concentration of ABA in the leaf tissue was calculated using the following formula:
ABA content in leaf (µg g⁻¹ F.W.) = (Xvial × V) / (W × R) * 100
where Xvial = Concentration in the HPLC vial (μg/mL), V = Final volume of the reconstituted extract (mL), W = Fresh weight of the leaf tissue (g), and R = Method Recovery Rate expressed as a fraction (0.758)."

Regards

**Point 3 (Clarification of Root Infection Mapping Methodology)**

**Reviewer Comment:** *Third, the authors state in the response letter that the gridline-intersect method for AMF colonization was clarified. However, the main Methods section still mainly describes clearing/staining and counting colonized root bits among 30 root segments per replication. If the gridline-intersect method was actually used, the authors should describe it explicitly and consistently. If the authors used a root-bit counting method rather than a gridline-intersect method, the response letter and Methods section should be corrected accordingly.*

**Author Response:** We appreciate this crucial corrective point. The previous response letter inadvertently included conflicting procedural nomenclature. Our research group executed a root-bit segment counting method rather than the gridline-intersect protocol. To establish clear procedural alignment, all text frames within Section 2.2 have been systematically verified to describe the root-bit segment counting approach uniformly, detailing the specific scanning steps for the 30 segments evaluated per replicate.

**Manuscript Text Modifications:**
The descriptive text in Section 2.2 (Root colonization analysis) has been structured as follows:
"The presence of fungal hyphae, arbuscules, and vesicles was examined under a 10X compound microscope. For each biological replication, 30 distinct root segments (each 1 cm long) were meticulously scanned using a modified root-bit segment counting method to verify structural infection parameters. The total per cent root colonisation was calculated using the following formula: Percent root colonization = (Number of root bits positive for colonization / Total number of root bits observed under microscope) × 100."

**Point 4 (Rectifying Language, Terminology, and Typographical Errors)**

**Reviewer Comment:** *Fourth, some language and typographical problems remain despite the authors’ statement that the manuscript was carefully edited. For example, errors such as 'mycrorrhizal plants,' 'Bacilus,' and 'to done the study' are still present in the manuscript. These errors should be corrected, and the manuscript should undergo another round of careful English editing. In addition, several awkward phrases remain, such as 'at just before given differential water treatment' and 'statistically insignificant with,' which should be revised for clarity and standard scientific English.*

**Author Response:** We apologize for these technical copy-editing oversights. The entire manuscript text has undergone an exhausting language cleanup and restructuring process. All identified typos have been corrected ('mycrorrhizal' to 'mycorrhizal'; 'Bacilus' to 'Bacillus'; institutional permission altered from 'to done the study' to 'to conduct the study'). Furthermore, the unscientific expression 'statistically insignificant with' has been replaced throughout all text descriptions and data table annotations with standard scientific phrases, such as 'statistically comparable to' or 'did not differ significantly from.' Non-idiomatic chronological placeholders have also been streamlined.

**Manuscript Text Modifications:**
Global substitutions were executed across the master document:
- All instances of 'at just before given differential water treatment' -> 'just prior to the imposition of differential moisture regimes' or 'before stress (BS)'.
- All instances of 'statistically insignificant with' -> 'statistically comparable to' or 'did not differ significantly from'.
- Re-checked and corrected the spelling of the genus name Bacillus and the adjective mycorrhizal in all locations.

**Point 5 (Softening Stomatal Overstatements and Genotype Rootstock Claims)**

**Reviewer Comment:** *Fifth, some unsupported or exaggerated statements remain in the Results and Discussion. For example, the manuscript still states that 'control plants suffered total stomatal closure,' although the reported stomatal conductance values are low but not zero. This should be revised to a more accurate statement, such as 'control plants showed significantly reduced stomatal conductance.' Similarly, the conclusion states that Troyer citrange performed as a superior rootstock compared to Cleopatra mandarin under water stress, but several genotype comparisons in the Discussion are described as statistically insignificant. Therefore, the conclusion should be more cautious and should avoid broad superiority claims unless directly supported by the statistical analysis.*

**Author Response:** We recognize the accuracy of the reviewer's comment. Because stomatal conductance metrics in the uninoculated control treatments were minimized but mathematically remained above zero, utilizing the phrase 'total closure' is factually incorrect. We have reworded this sentence to state 'significantly reduced stomatal conductance.' Additionally, because specific dual-inoculated parameters between the two rootstocks showed cross-over statistical parities, we have carefully re-phrased our abstract and primary conclusion blocks to frame the performance of Troyer citrange relative to the specific physiological, hydraulic, and carbon assimilation matrices evaluated, avoiding generalized superiority claims.

**Manuscript Text Modifications:**
1. Section 3.10 (Stomatal behaviors text): "Conversely, uninoculated control plants showed significantly reduced stomatal conductance, which led to marked photosynthetic inhibition."
2. Section 5 (Conclusions): "Based on the monitored water relations and carbon assimilation matrices, Troyer citrange rootstock demonstrated a more functional physiological buffer profile under water deficit stress than Cleopatra mandarin."
3. Abstract (Final Sentence): "Under these experimental conditions, Troyer citrange exhibited higher relative water content, photosynthetic activity, and stomatal conductance under drought than Cleopatra mandarin, offering a potential microbial intervention strategy..."

**Point 6 (Clarifying the 'Individual Potted Seedling' Experimental Unit & Correlation Matrix)**

**Reviewer Comment:** *Sixth, the correlation analysis in Table 7 still needs clearer explanation. The table reports correlations based on n = 24 treatment nodes, which suggests that correlations were calculated using treatment means rather than independent biological replicates. This should be clearly stated in the table note and interpreted cautiously. The current note is confusing because it states that each replication consisted of six plants as experimental units, whereas the statistical section states that the pot was the experimental unit. This should be harmonized to avoid ambiguity.*

**Author Response:** The reviewer’s observation is entirely accurate. The Pearson correlation values were calculated strictly from the pooled treatment mean nodes (n = 24 total nodes) to illuminate broad physiological and biochemical co-regulatory trajectories across the various treatment matrices. We have updated Section 3.18 to state this clearly.

Furthermore, we have resolved the layout ambiguity across the Methods, Results, and Table Footnotes: our layout featured six seedlings grown individually in six separate pots per replication (one uniform seedling per pot), where each individual pot and its seedling functioned as a distinct, independent experimental unit (yielding 12 pots per treatment per year, compiled to n = 6 true biological replicates across the pooled three-year dataset). All sections have been fully synchronized with this definition to eliminate confusion.

**Manuscript Text Modifications:**
1. Section 2.1 (Experimental Setup layout text): "Each replication consisted of six separate pots, with one uniform seedling grown per pot, where each individual potted seedling served as a distinct independent experimental unit (yielding 12 pots per treatment per year, and a pooled sample size of n = 6 true biological replicates per treatment combination across the three years)."
2. Section 3.18 (Correlation Text update): "These coefficients were evaluated across the 24 treatment mean nodes to track global physiological trajectories across the treatments."
3. Table 7 Footnote (Harmonized text): "Note: Correlations were calculated using the treatment mean nodes (n = 24) derived from pooled annual runs. Within the experimental setup, each individual potted seedling served as a distinct independent experimental unit, yielding six true biological replications per treatment across the pooled dataset. Significance indicators: * details p < 0.05; ** details p < 0.01 (SPSS 16.0)."

**Reviewer #2:**

We express our sincere appreciation to Reviewer #2 for evaluating and accepting of our manuscript.

**Reviewer Comment:** *Revised manuscript addressed all comments previously raised. It should be accepted for publication.*

**Author Response:** We sincerely thank Reviewer #2 for reviewing our structural amendments and validating our scientific updates. We are grateful for the endorsement for publication.
