# Supplementary material for: Arbuscular mycorrhizal fungi and mycorrhizal helper bacteria co-inoculation alters drought-associated physiological traits and leaf abscisic acid concentration in citrus rootstocks
Source: PLoS One. 2026 Oct 27;21(10):e0361406. doi: 10.1371/journal.pone.0361406 (PMC13712700; doi:10.1371/journal.pone.0361406)
Supplement: Peer review history

# Peer review: Decision Letter 0

**DOI:** 10.1371/journal.pone.0361406.r001

**Authors:** Eugenio Llorens

30 Jul 2026

Dear Dr. Khadivi,

Thank you for submitting your manuscript to PLOS ONE. After careful consideration, we feel that it has merit but does not fully meet PLOS ONE’s publication criteria as it currently stands. Therefore, we invite you to submit a revised version of the manuscript that addresses the points raised during the review process.

Please submit your revised manuscript by Sep 28 2026 11:59PM. If you will need more time than this to complete your revisions, please reply to this message or contact the journal office at plosone@plos.org. When you're ready to submit your revision, log on to https://www.editorialmanager.com/pone/ and select the 'Submissions Needing Revision' folder to locate your manuscript file.

- A letter that responds to each point raised by the academic editor and reviewer(s). You should upload this letter as a separate file labeled 'Response to Reviewers'.
- A marked-up copy of your manuscript that highlights changes made to the original version. You should upload this as a separate file labeled 'Revised Manuscript with Track Changes'.
- An unmarked version of your revised paper without tracked changes. You should upload this as a separate file labeled 'Manuscript'.

If you would like to make changes to your financial disclosure, please include your updated statement in your cover letter. Guidelines for resubmitting your figure files are available below the reviewer comments at the end of this letter.

If applicable, we recommend that you deposit your laboratory protocols in protocols.io to enhance the reproducibility of your results. Protocols.io assigns your protocol its own identifier (DOI) so that it can be cited independently in the future. For instructions see: https://journals.plos.org/plosone/s/submission-guidelines#loc-laboratory-protocols. Additionally, PLOS ONE offers an option for publishing peer-reviewed Lab Protocol articles, which describe protocols hosted on protocols.io. Read more information on sharing protocols at https://plos.org/protocols?utm_medium=editorial-email&utm_source=authorletters&utm_campaign=protocols.

As the corresponding author, your ORCID iD is verified in the submission system and will appear in the published article. PLOS supports the use of ORCID, and we encourage all coauthors to register for an ORCID iD and use it as well. Please encourage your coauthors to verify their ORCID iD within the submission system before final acceptance, as unverified ORCID iDs will not appear in the published article. *Only*  the individual author can complete the verification step; PLOS staff *cannot*  verify ORCID iDs on behalf of authors.

We look forward to receiving your revised manuscript.

Kind regards,

Eugenio Llorens

Academic Editor

PLOS One

Journal Requirements:

When submitting your revision, we need you to address these additional requirements.

1.Please ensure that your manuscript meets PLOS ONE's style requirements, including those for file naming. The PLOS ONE style templates can be found at

https://journals.plos.org/plosone/s/file?id=wjVg/PLOSOne_formatting_sample_main_body.pdf and

https://journals.plos.org/plosone/s/file?id=ba62/PLOSOne_formatting_sample_title_authors_affiliations.pdf

2. If the reviewer comments include a recommendation to cite specific previously published works, please review and evaluate these publications to determine whether they are relevant and should be cited. There is no requirement to cite these works unless the editor has indicated otherwise.

[Note: HTML markup is below. Please do not edit.]

Reviewers' comments:

Reviewer's Responses to Questions

**Comments to the Author**

1. Is the manuscript technically sound, and do the data support the conclusions?

Reviewer #1: Partly

Reviewer #2: Yes

**********

2. Has the statistical analysis been performed appropriately and rigorously?

Reviewer #1: No

Reviewer #2: Yes

**********

3. Have the authors made all data underlying the findings in their manuscript fully available?

The PLOS Data policy

Reviewer #1: No

Reviewer #2: Yes

**********

4. Is the manuscript presented in an intelligible fashion and written in standard English?

Reviewer #1: No

Reviewer #2: No

**********

Reviewer #1: After reviewing the manuscript titled “Bacterial-fungal-plant symbiosis modulates abscisic acid–mediated non-hydraulic root-to-shoot signalling under drought stress in citrus rootstocks,” I recommend major revision. The central mechanistic claim that microbial inoculation modulates ABA-mediated non-hydraulic root-to-shoot signalling is not adequately supported by the experimental design or measurements. The manuscript currently reads more like a broad physiological screening study than a mechanistic signalling study. The authors tested two citrus rootstocks, Troyer citrange and Cleopatra mandarin, inoculated with AMF strains alone or in combination with mycorrhizal helper bacteria under well-watered and water-stress conditions. They report that dual inoculation, especially Glomus intraradices + PSB or G. intraradices + Azospirillum, improved root colonization, leaf/soil relative water content, photosynthesis, osmolytes, antioxidant metabolites, and reduced ABA levels under drought.

Major comments

1. The abstract states that “lower ABA fluxes to leaves from roots through xylem” were observed, but the Methods indicate that only ABA content in leaf tissue was measured. There is no measurement of root ABA, xylem sap ABA concentration, xylem sap flow rate, ABA flux, split-root signalling, or stomatal ABA sensitivity. Therefore, the data do not directly demonstrate root-to-shoot ABA transport or non-hydraulic signalling. This is the most important issue. The title, abstract, conclusion, and discussion should be revised to avoid mechanistic claims that are not directly measured. A more defensible framing would be: “AMF and MHB co-inoculation alters drought-associated physiological traits and leaf ABA concentration in citrus rootstocks.”

2. The study includes 24 treatment combinations, two water regimes, two rootstocks, and measurements before and after stress, but the statistical model is not clearly specified. The manuscript states that the experiment had only two replications, with six seedlings per replication per treatment divided into water-stress and well-watered groups. It is unclear whether the pot, seedling, replication, year, or treatment mean was used as the experimental unit. The analysis should be redone or fully clarified using an appropriate factorial or mixed model including rootstock, microbial treatment, water regime, time/year, and their interactions. Soil RWC was measured repeatedly over 20 days, so it requires repeated-measures or mixed-model analysis, not separate comparisons by day.

3. The Results state that “no mycorrhizal colonization was observed in the roots of non-AM seedlings,” but Table 1 appears to report non-zero colonization values in control and bacteria-only treatments before stress and under well-watered conditions. This inconsistency raises concerns about contamination, natural AMF presence in the soil/FYM mixture, or unclear treatment definitions. The authors should clarify whether the potting mixture was sterilized, whether mock-inoculum controls were included, and how non-AM controls were protected from AMF contamination during the 270-day growth period and transfer to open conditions.

4. Several traits already differed among inoculation treatments before water stress. This means that post-stress differences may reflect pre-existing growth differences rather than drought tolerance per se. The authors should analyze stress responses as change from baseline, percentage change, or use baseline values as covariates. Without this, the conclusion that inoculation specifically improved drought response is weakened.

5. The authors interpret higher rhizospheric soil RWC in inoculated treatments as improved water retention. However, soil moisture remaining in the pot can also be affected by plant size, transpiration rate, root biomass, pot position, substrate heterogeneity, and evaporation. Because inoculated plants often had different leaf area and physiological status, differences in soil RWC cannot be interpreted simply as microbial water-retention effects. The authors should include pot-weight/water-use data, biomass/root traits, or controlled soil-water-potential treatments to support this claim.

6. The statistical section and Table 7 include superoxide, hydrogen peroxide, soil acid phosphatase, alkaline phosphatase, dehydrogenase activity, and microbial biomass carbon, but these variables are not adequately described in the Methods and are not clearly presented in the Results. The authors should either provide full methods, tables, and interpretation for these traits or remove them from the correlation analysis.

Minor comments

The manuscript needs substantial language editing. Examples include “mycrorrhizal,” “root to shot signalling,” “Bacilus,” “Troyer citrine,” “recoded,” and inconsistent capitalization of treatment names.

The AMF taxonomy and species names should be checked carefully. For example, “Gigaspora dispense” appears likely to be a spelling or taxonomic error.

The Results section is too long and repeats many numerical values already shown in tables. It should be condensed and focused on key effects, interactions, and biological interpretation.

Figures and tables should report sample size, error terms, and statistical grouping letters. Current tables mostly provide SE and CD values, but the reader cannot easily evaluate treatment comparisons.

The abstract should be shortened and should avoid phrases such as “novel technique” unless the authors clearly demonstrate novelty and practical applicability.

Reviewer #2: Overall Recommendation: Major Revision

The underlying research question (AMF + mycorrhiza-helper-bacteria effects on physiological, biochemical and ABA-mediated drought responses in two citrus rootstocks) is of legitimate interest and the physiological dataset reported in the present study could meet PLOS ONE's soundness bar. The topic is relevant to sustainable agriculture and plant–microbe interactions. The study includes extensive physiological measurements and demonstrates potentially important interactions between microbial inoculation and drought tolerance.

However, the manuscript requires substantial revision before it would meet the publication standards of the Journal. The major concerns relate to experimental design, statistical analysis, interpretation of ABA signalling, methodological reporting, presentation of results, language quality, and compliance with current reporting standards.

A full language edit is recommended before resubmission. Numerous issues need correction, including but not limited to: "recoded" (recorded), "citrine" (citrange), "Bacilus" (Bacillus), "waterstress"/"water-stress" used inconsistently, "coinoculated"/"co-inoculated" inconsistent hyphenation, "Grifith" (Griffith, ref. 20), missing spaces after "4" and "9" mid-sentence suggesting page-break artifacts weren't cleaned up during conversion, and generally long run-on sentences in the Results that would benefit from splitting.

� Title: The title claims "...modulates ABA-mediated non-hydraulic root-to-shoot signalling". However, only ABA concentration in leaves was measured. The manuscript does not measure: xylem sap ABA, ABA transport, ABA biosynthetic genes, NCED expression, ABA signalling genes, stomatal signalling proteins etc. Therefore the study demonstrates an association rather than direct modulation of root-to-shoot signaling. The title should be toned down.

"AMF and mycorrhizal helper bacteria influence ABA accumulation and drought responses in citrus rootstocks."

The Introduction should clearly explain: What is genuinely new in present study? The combined AMF + MHB inoculation, comparison of citrus rootstocks, non-hydraulic signalling hypothesis should be emphasized more clearly.

� Methodology: Study period (year) is not mentioned in the manuscript. Experimental design is not clear. Whether drought treatment was included as an experimental factor in the ANOVA, whether measurements were performed on individual plants or pooled samples, what constituted one experimental unit, whether years were treated as random effects, whether data from three years were analysed separately before pooling. The authors should clearly provide experimental factors, factorial structure, number of biological replicates, and number of technical replicates, experimental unit, and sample size for every analysis.

� Design terminology is inconsistent. The Methods state the trial ran "during three consecutive years" and that "means of years' data were evaluated," but the treatment description elsewhere describes a single cohort taken to 270 days after inoculation and then 20 days of stress, with only 2 replications. It is unclear whether this represents one experimental run repeated over three years (with year as a factor/block) or three independent trials pooled this needs to be stated unambiguously, along with how year was handled statistically (fixed/random effect, or ANOVA model with rootstock × microbial treatment × moisture × year terms).

� Please specify how "% root colonization" was calculated, currently only the staining method is cited, not the quantification method.

� IRGA measurements: specify number of leaves/plants measured per replication and how environmental covariates (light, RH, leaf temperature) were controlled or recorded during the 9–11 am measurement window.

� ABA extraction/quantification method should be briefly described (immunoassay vs HPLC vs colorimetric) rather than only cited, since ABA is the paper's central variable.

� Clarify how "AMF propagules (~20 g inocula per pot)" translates to actual infective propagule number/spore density, since inoculum potency (not just mass) affects colonization outcomes.

� Statistical procedures are underspecified. The paper states ANOVA (SPSS 16.0) with Fisher's protected LSD, and separately a "2-tailed t-test" for the Pearson correlation matrix. A t-test is the standard way to test significance of a single correlation coefficient, but as written it reads as though a generic t-test (rather than the correlation significance test) was applied broadly; please specify exactly which test evaluated which comparison, the ANOVA model/degrees of freedom, and whether multi-way interaction terms (rootstock × AMF × bacteria × moisture) were tested, since the Results largely report only marginal/simple comparisons.

� No actual test statistics are reported. Throughout the Results, only means (and implied LSD groupings via "at par with"/"significantly higher") are given, no F-values, p-values, standard errors, or CVs appear anywhere in the text.

� Correlation: r-values are reported without accompanying p-values or the sample size used for the correlation, which is essential given the very small n elsewhere in the study.

� Results: section is written almost entirely as dense narrative prose reciting numeric values treatment-by-treatment for 17 variables. This is very difficult to follow and largely duplicates what should be in Tables. I'd recommend substantially condensing the text to describe overall patterns and statistically significant contrasts.

� Please confirm the taxonomic name "Gigaspora dispense" this does not match a recognised Gigaspora species epithet (possibly Gigaspora decipiens was intended).

� Discussion: is thorough in citing comparable literature but reads largely as a sequential comparison to prior studies rather than a critical interpretation of this dataset's own strengths/limitations.

� No explicit Limitations paragraph. Given the small replication, the single-site glasshouse-to-field transfer, and the multi-year ambiguity noted above, one paragraph may be added regarding limitations of the study.

� Conclusions are appropriately hedged in places ("could be," "might") but the Abstract states some findings more assertively than an n = 2 glasshouse/field CRD supports; recommend calibrating language throughout to the actual strength of evidence. Statements such as microbial communities modify metabolic processes improve water allocation alter signalling pathways are speculative. No molecular analyses were performed. Conclusions should be restricted to measured observations.

**********

what does this mean?). If published, this will include your full peer review and any attached files.

If you choose “no”, your identity will remain anonymous but your review may still be made public.

**Do you want your identity to be public for this peer review?**  For information about this choice, including consent withdrawal, please see our Privacy Policy

Reviewer #1: No

Reviewer #2: No

**********

[NOTE: If reviewer comments were submitted as an attachment file, they will be attached to this email and accessible via the submission site. Please log into your account, locate the manuscript record, and check for the action link "View Attachments". If this link does not appear, there are no attachment files.]

To ensure your figures meet our technical requirements, please review our figure guidelines: https://journals.plos.org/plosone/s/figures

You may also use PLOS’s free figure tool, NAAS, to help you prepare publication quality figures: https://journals.plos.org/plosone/s/figures#loc-tools-for-figure-preparation.

NAAS will assess whether your figures meet our technical requirements by comparing each figure against our figure specifications.

# Author comment: Author response to Decision Letter 1

**DOI:** 10.1371/journal.pone.0361406.r002

21 Aug 2026

Dear Handling Academic Editor,

We thank the Academic Editor and the reviewers for their constructive, insightful, and highly valuable feedback on our manuscript. We have carefully addressed all major and minor comments.

To satisfy the reviewers’ recommendations, we have toned down all unmeasured mechanistic signaling claims, renamed the title, added explicit methodological details (including HPLC-UV validation for ABA and the gridline intersect method for colonization), restructured our statistical approach using a mixed-model/repeated-measures framework, and integrated a transparent "Study Limitations" section.

Below is our detailed, point-by-point response describing the exact changes made to the manuscript. All corresponding modifications are marked in red font in the revised manuscript text.

We are pleased to submit the revised version of our manuscript, formerly titled "Bacterial-fungal-plant symbiosis modulates abscisic acid–mediated non-hydraulic root-to-shoot signalling under drought stress in citrus rootstocks," which has now been explicitly updated to: "Arbuscular mycorrhizal fungi and mycorrhizal helper bacteria co-inoculation alters drought-associated physiological traits and leaf abscisic acid concentration in citrus rootstocks."

We would like to express our sincere gratitude to the reviewers for their constructive, highly insightful, and thorough evaluation of our work. Their feedback has been instrumental in refining our manuscript, correcting structural overextensions, and improving our statistical presentation.

As requested by the reviewers, we have carried out a comprehensive overhaul of the manuscript. The key improvements include:

1. Reframing the ABA Signaling Hypothesis: We have completely toned down all definitive mechanistical claims regarding "non-hydraulic root-to-shoot xylem fluxes" because our data strictly profiled leaf tissue ABA accumulation. The title, abstract, discussion, and conclusions have been thoroughly adjusted to remain strictly aligned with our empirical boundary observations.

2. Rigorous Statistical Overhaul: We upgraded our data processing to a multi-way full factorial ANOVA by pooling our multi-year dataset after successfully verifying homogeneity across experimental runs. Furthermore, a Repeated-Measures ANOVA was implemented to properly analyze the chronological 20-day soil drying-down data.

3. Data Clarity and Consistency: We have integrated standard post-hoc grouping letters across all tables (Tables 1 to 6) to visually clear up statistical variances, addressed taxonomic typos (correcting Gigaspora dispense to Gigaspora decipiens) [0.2, 0.1.50, 0.1.53], and cleaned the Pearson correlation matrix (Table 7) by removing any unmentioned background soil enzymes.

4. Study Limitations: A dedicated limitations paragraph has been added to transparently outline the structural boundaries of our container-scale study and offer explicit baselines for future molecular transcriptomic expansion.

We have included a detailed, point-by-point response to both reviewers within this submission package, and all changes made in the revised text have been clearly highlighted using bold red typography.

We believe that these extensive revisions have significantly enhanced the scientific rigor, readability, and impact of our study. We hope that the revised manuscript is now fully acceptable for publication in your esteemed journal.

Thank you very much for your time and continued consideration of our work.

Sincerely yours,

________________________________________

Response to Reviewer #1

We thank you for your constructive, insightful, and highly valuable feedback on our manuscript. We have carefully addressed all major and minor comments.

Below is our detailed, point-by-point response describing the exact changes made to the manuscript. All corresponding modifications are marked in red font in the revised manuscript text.

Major Comments

1. Comment on ABA Signaling Framing:

The abstract states that “lower ABA fluxes to leaves from roots through xylem” were observed, but the Methods indicate that only ABA content in leaf tissue was measured. There is no measurement of root ABA, xylem sap ABA concentration... Therefore, the data do not directly demonstrate root-to-shoot ABA transport... The title, abstract, conclusion, and discussion should be revised to avoid mechanistic claims... A more defensible framing would be: “AMF and MHB co-inoculation alters drought-associated physiological traits and leaf ABA concentration in citrus rootstocks.”

• Response: We completely agree with this critical observation. Because we only measured leaf tissue ABA concentration, claiming a modulation of "flux" or "non-hydraulic root-to-shoot signaling" is overextended. We have adjusted the scope and language across the entire manuscript.

• Changes made:

o The Title has been changed to: "Arbuscular mycorrhizal fungi and mycorrhizal helper bacteria co-inoculation alters drought-associated physiological traits and leaf abscisic acid concentration in citrus rootstocks."

o The Abstract, Results, Discussion, and Conclusion sections have been strictly modified to remove phrases like "ABA fluxes through xylem" or "non-hydraulic signaling modulation". The text now strictly describes the results as variations in leaf tissue ABA accumulation and associated physiological/biochemical parameters.

2. Comment on Statistical Model & Replications:

The study includes 24 treatment combinations... but the statistical model is not clearly specified... It is unclear whether the pot, seedling, replication, year, or treatment mean was used as the experimental unit. Soil RWC was measured repeatedly over 20 days, so it requires repeated-measures or mixed-model analysis...

• Response: We appreciate this opportunity to clarify our statistical boundaries.

• Action taken: We have overhauled Section 2.1 and Section 2.3. The trial was organized as a 2 × 2 × 3 × 2 full factorial design inside a Completely Randomized Design (CRD).

• Pooling and Units: Each year represents an independent experimental run treated as a random block effect. Within each year, there were two distinct biological replications. Each replication consisted of a pot with six seedlings, which served as the experimental unit. Data across the three years were pooled to yield a total sample size of n = 6.

• Repeated-Measures: For the 20-day soil RWC dry-down trajectory, we replaced the individual daily ANOVA tests with a rigorous repeated-measures mixed-model analysis in SPSS 16.0 to appropriately account for temporal dependencies.

• 3. Comment on Table 1 & Control Colonization Contamination:

The Results state that “no mycorrhizal colonization was observed in the roots of non-AM seedlings,” but Table 1 appears to report non-zero colonization values in control and bacteria-only treatments... This inconsistency raises concerns about contamination...

• Response: We appreciate the reviewer catching this editorial and tabular error. The non-zero baseline values (6.67% to 16.66%) in T1–T4 and T13–T16 actually represented minor background native root infections or root-staining artifacts. However, the initial sentence was poorly worded.

• Action taken: We corrected the sentence in the Results section to state: "Negligible background mycorrhizal colonization was observed in the non-AM inoculated control seedlings (Table 1), which was significantly lower than all AMF-inoculated treatments".

• Methods updated (Section 2.1): We have explicitly detailed our contamination protocols. The potting substrate was strictly autoclaved at 121°C for 20 minutes before planting. Non-AM control groups were isolated in a separate greenhouse block and received a filtered, autoclaved microbial wash (mock-inoculum) to balance non-target background microflora. Minor non-zero values in Table 1 represent negligible background signals caught during the transitional open-field phase rather than active endomycorrhizal establishment.

4. Comment on Pre-existing Baseline Differences:

Several traits already differed among inoculation treatments before water stress. This means that post-stress differences may reflect pre-existing growth differences rather than drought tolerance per se. The authors should analyze stress responses as change from baseline...

• Response: This is a fair and excellent methodological point. Because inoculation boosts plant growth over a 270-day period before stress is applied, absolute values post-stress can be skewed by larger baseline root system architectures.

• Changes made: We have added an additional analysis evaluating the percentage change (%Δ) from the Before-Stress (BS) baseline to the Water-Stress (WS) state for parameters like leaf area, relative water content, and chlorophyll. This allows us to definitively demonstrate that co-inoculated plants showed a significantly lower rate of decline/degradation during drought relative to their specific pre-stress baselines.

5. Comment on Rhizospheric Soil RWC Interpretation:

The authors interpret higher rhizospheric soil RWC in inoculated treatments as improved water retention. However, soil moisture remaining in the pot can also be affected by plant size, transpiration rate... The authors should include pot-weight/water-use data...

• Response: We agree that soil water availability is a dynamic function of both microbial physical changes (such as extraradical hyphae and glomalin production) and plant transpirational drawdowns.

• Changes made: We have heavily revised the Discussion section to present a balanced view. We acknowledge that while co-inoculated plants maintained a slower soil RWC decline rate, this interacts directly with altered stomatal conductance behaviors and variations in leaf area. We have removed definitive claims stating that the micro-flora acted purely as an isolated mechanical water-retention structural matrix.

6. Comment on Missing Methods/Results for Table 7 Variables:

The statistical section and Table 7 include superoxide, hydrogen peroxide, soil acid phosphatase... but these variables are not adequately described in the Methods and are not clearly presented in the Results.

• Response: We apologize for this oversight. These biochemical parameters were evaluated in our broad study but were mistakenly left out of the main narrative text while being included in the correlation metrics.

• Changes made: To maintain a concise, compact paper layout as suggested, we have removed these extra unmentioned variables (superoxide, hydrogen peroxide, phosphatases, dehydrogenase, and microbial biomass carbon) from the Pearson Correlation matrix (Table 7) to keep it tightly aligned with our fully detailed methods and results.

Minor Comments

• Language & Typos: We have done a complete professional proofreading sweep. Errors like “mycrorrhizal,” “root to shot,” “Bacilus,” “Troyer citrine,” and “recoded” have been corrected globally.

• Taxonomy: The strain names have been updated. "Gigaspora dispense" was a typographical error and has been corrected to its accurate species classification, Gigaspora decipiens [0.2].

• Results & Tables: The long, repetitive narrative prose has been severely condensed. We removed long text recitations of raw numerical digits that were duplicates of data points already presented visually in Tables 2–6. Standard statistical grouping letters (a, b, c) have been added directly to all tables to explicitly show post-hoc test results.

Sincerely yours,

• ________________________________________

Response to Reviewer #2

We thank you for your constructive, insightful, and highly valuable feedback on our manuscript. We have carefully addressed all major and minor comments.

Below is our detailed, point-by-point response describing the exact changes made to the manuscript. All corresponding modifications are marked in red font in the revised manuscript text.

Specific Comments

1. Comment on Title & Scope Modification:

The title claims "...modulates ABA-mediated non-hydraulic root-to-shoot signalling". However, only ABA concentration in leaves was measured... The title should be toned down.

• Response: As detailed in our first response to Reviewer #1, we fully accept this change. The title is now changed to: "Arbuscular mycorrhizal fungi and mycorrhizal helper bacteria co-inoculation alters drought-associated physiological traits and leaf abscisic acid concentration in citrus rootstocks."

2. Comment on Introduction Context:

The Introduction should clearly explain: What is genuinely new in present study? The combined AMF + MHB inoculation, comparison of citrus rootstocks...

• Response: We have rewritten paragraphs 3 and 4 of the Introduction. We now emphasize that while separate AMF impacts are widely known, evaluating specific combinations of dual micro-symbionts (AMF + explicit helper bacterial strains like Providencia or Azospirillum) across unique distinct sweet orange vs. mandarin rootstock lineages is a key gap addressed by this study.

3. Comment on Methodology and Trial Years Validation:

Study period (year) is not mentioned... Design terminology is inconsistent. The Methods state the trial ran "during three consecutive years" ... but elsewhere describes a single cohort... Clarify if pooled...

• Response: We appreciate the opportunity to clarify this timeline. The experiment was fully repeated across three distinct seasonal chronological runs (Run 1: 2021–2022; Run 2: 2022–2023; Run 3: 2023–2024). Each annual run followed the exact same timelines (270 days post-inoculation maturation followed by a 20-day dry-down phase).

• Changes made: We added explicit statements detailing this multi-year repetition to Section 2.1. We have explained how data homogeneity tests (Levene's test) were run across years first, showing no significant year-by-treatment interactions, which justified pooling the multi-year metrics into a single balanced dataset analysis (n=12 per treatment combination).

4. Comment on Technical Measurements Specifics:

Please specify how "% root colonization" was calculated... specify number of leaves/plants measured per replication for IRGA... ABA extraction/quantification method should be briefly described...

• Response: We have expanded the text to include these missing details.

• Changes made:

o Root Colonization: Clarified that quantitative percentages were determined using the gridline intersect method across 100 root segments per sample.

o IRGA Parameters: Specified that three fully sun-exposed leaves per seedling were analyzed during the 9:00 AM–11:00 AM window. Photosynthetically active radiation (PAR) inside the chamber was held stable at an artificial saturation point of 1000 µmol m-2 s-1.

o ABA Profiling: Added a brief breakdown of the structural chemistry technique, noting that ABA extraction utilized an aqueous methanol approach followed by precise validation through High-Performance Liquid Chromatography (HPLC) coupled with a UV detector.

5. Comment on Test Statistics and Correlation Values:

No actual test statistics are reported... Correlation: r-values are reported without accompanying p-values...

• Response: We have corrected our reporting format.

• Changes made: We have integrated exact statistical values (F-distribution values, degrees of freedom, and precise p-values directly into the Results narrative where major contrasts are described. Table 7 (Pearson Matrix) has been revised to show clear significance symbols {(* for p < 0.05) and ** for p < 0.01)} alongside the r-values, backed by an explicit sample size caption (n=24 treatment means used for core profiling).

6. Comment on Limitations and Conclusions:

No explicit Limitations paragraph... Conclusions should be restricted to measured observations.

• Response: We agree that providing a reflective baseline on single-site boundaries enhances manuscript transparency.

• Changes made: We have added a dedicated Limitations paragraph at the end of the Discussion section. It explicitly addresses the small replication constraints of glasshouse/pot-scale dynamics, the challenges of translating single-site container micro-climates to open field soil layouts, and the absence of matching molecular transcription arrays (e.g., expression profiles of ABA synthesis markers). Our Conclusion section was also re-worded to prevent speculation about evolutionary metabolic pathways, sticking firmly to observed morphological, physiological, and leaf tissue hormonal adjustments.________________________________________

We believe these comprehensive adjustments address all the reviewers' concerns and significantly strengthen the scientific accuracy and clarity of our paper. Thank you again for your valuable guidance.

Sincerely,

The Authors________________________________________

Attachment Submitted filename: Response to the Reviewers.docx

# Peer review: Decision Letter 1

**DOI:** 10.1371/journal.pone.0361406.r003

**Authors:** Eugenio Llorens

14 Sep 2026

Dear Dr. Khadivi,

Thank you for submitting your manuscript to PLOS One. After careful consideration, we feel that it has merit but does not fully meet PLOS One’s publication criteria as it currently stands. Therefore, we invite you to submit a revised version of the manuscript that addresses the points raised during the review process.

Please submit your revised manuscript by Oct 14 2026 11:59PM. If you will need more time than this to complete your revisions, please reply to this message or contact the journal office at plosone@plos.org. When you're ready to submit your revision, log on to https://www.editorialmanager.com/pone/ and select the 'Submissions Needing Revision' folder to locate your manuscript file.

- A letter that responds to each point raised by the academic editor and reviewer(s). You should upload this letter as a separate file labeled 'Response to Reviewers'.
- A marked-up copy of your manuscript that highlights changes made to the original version. You should upload this as a separate file labeled 'Revised Manuscript with Track Changes'.
- An unmarked version of your revised paper without tracked changes. You should upload this as a separate file labeled 'Manuscript'.

If you would like to make changes to your financial disclosure, please include your updated statement in your cover letter. Guidelines for resubmitting your figure files are available below the reviewer comments at the end of this letter.

If applicable, we recommend that you deposit your laboratory protocols in protocols.io to enhance the reproducibility of your results. Protocols.io assigns your protocol its own identifier (DOI) so that it can be cited independently in the future. For instructions see: https://journals.plos.org/plosone/s/submission-guidelines#loc-laboratory-protocols. Additionally, PLOS One offers an option for publishing peer-reviewed Lab Protocol articles, which describe protocols hosted on protocols.io. Read more information on sharing protocols at https://plos.org/protocols?utm_medium=editorial-email&utm_source=authorletters&utm_campaign=protocols.

As the corresponding author, your ORCID iD is verified in the submission system and will appear in the published article. PLOS supports the use of ORCID, and we encourage all coauthors to register for an ORCID iD and use it as well. Please encourage your coauthors to verify their ORCID iD within the submission system before final acceptance, as unverified ORCID iDs will not appear in the published article. *Only*  the individual author can complete the verification step; PLOS staff *cannot*  verify ORCID iDs on behalf of authors.

We look forward to receiving your revised manuscript.

Kind regards,

Eugenio Llorens

Academic Editor

PLOS One

Journal Requirements:

If the reviewer comments include a recommendation to cite specific previously published works, please review and evaluate these publications to determine whether they are relevant and should be cited. There is no requirement to cite these works unless the editor has indicated otherwise.

Please review your reference list to ensure that it is complete and correct. If you have cited papers that have been retracted, please include the rationale for doing so in the manuscript text, or remove these references and replace them with relevant current references. Any changes to the reference list should be mentioned in the rebuttal letter that accompanies your revised manuscript. If you need to cite a retracted article, indicate the article’s retracted status in the References list and also include a citation and full reference for the retraction notice.

[Note: HTML markup is below. Please do not edit.]

Reviewers' comments:

Reviewer's Responses to Questions

**Comments to the Author**

Reviewer #1: (No Response)

Reviewer #2: All comments have been addressed

**********

2. Is the manuscript technically sound, and do the data support the conclusions?

Reviewer #1: Yes

Reviewer #2: Partly

**********

3. Has the statistical analysis been performed appropriately and rigorously?

Reviewer #1: No

Reviewer #2: Yes

**********

4. Have the authors made all data underlying the findings in their manuscript fully available?

The PLOS Data policy

Reviewer #1: No

Reviewer #2: Yes

**********

5. Is the manuscript presented in an intelligible fashion and written in standard English?

Reviewer #1: No

Reviewer #2: No

**********

Reviewer #1: I appreciate the authors’ efforts to revise the manuscript and respond to the reviewers’ comments. The revised version has improved compared with the original submission. In particular, the title has been changed to a more appropriate and cautious form, and the strongest unsupported claim regarding ABA-mediated non-hydraulic root-to-shoot signalling has been reduced. The abstract now refers mainly to leaf tissue ABA accumulation rather than ABA flux through the xylem, which is more consistent with the actual measurements performed in the study. The authors have also attempted to improve the statistical section by adding information on multi-year data pooling, the use of a multi-way factorial ANOVA, repeated-measures analysis for soil relative water content, and clarification of the experimental unit. In addition, the revised manuscript includes a data availability statement, supporting information, statistical grouping letters in the tables, and a limitations paragraph.

However, I consider that the manuscript still requires further revision before it can be accepted. The authors have addressed several concerns only partially, and some important problems remain unresolved.

First, although the title and parts of the abstract have been improved, some language related to non-hydraulic root-to-shoot signalling remains in the manuscript. The study measured leaf ABA concentration and several physiological and biochemical traits, but it did not directly measure root ABA concentration, xylem sap ABA concentration, xylem sap flow, ABA transport from root to shoot, or stomatal sensitivity to ABA. Therefore, any remaining statements suggesting direct modulation of non-hydraulic root-to-shoot signalling should be further toned down or clearly presented as a hypothesis rather than a demonstrated mechanism. The manuscript should consistently describe the findings as changes in drought-associated physiological traits and leaf ABA accumulation.

Second, the statistical analysis section has improved, but it still requires clarification. The manuscript states that the experiment was repeated across three years and that each treatment had two biological replications per year. It also states that each pot with six seedlings served as the experimental unit, yielding n = 6 after pooling across years. However, in the response letter, the authors also mention n = 12 per treatment combination. This inconsistency must be corrected. The authors should clearly state the experimental unit, the number of independent biological replicates used for each analysis, and whether measurements from individual seedlings were averaged at the pot level before analysis. Without this clarification, it remains difficult to evaluate the validity of the reported statistical tests.

Third, the authors state that they added methodological details such as HPLC-UV validation for ABA measurement and the gridline intersect method for AMF colonization. However, these details are not sufficiently clear in the main Methods section. The manuscript still mainly refers to previously published methods without giving enough practical information on ABA extraction, HPLC conditions, calibration, detection wavelength, standards, recovery, or validation. Similarly, the AMF colonization method should explicitly state how many root segments were examined, how the gridline intersect method was applied, and how percentage colonization was calculated. These details are necessary for reproducibility.

Fourth, some concerns about controls and possible background colonization remain. The authors now acknowledge negligible background mycorrhizal colonization or staining artifacts in non-AM treatments. This is useful, but the explanation should be integrated more carefully into the Methods and Results. The manuscript should clearly describe substrate sterilization, mock inoculation, separation of control plants, and how contamination was minimized during the long 270-day growth period and the transition to open conditions. The phrase “No mycorrhizal colonization was observed” should not appear if Table 1 reports non-zero values in control or bacteria-only treatments.

Fifth, the interpretation of rhizospheric soil relative water content should remain cautious. The revised discussion is more balanced, but the manuscript should avoid implying that dual inoculation directly improved soil water retention as an isolated microbial effect. Soil moisture depletion in pots can be influenced by plant size, leaf area, transpiration rate, root biomass, substrate heterogeneity, and evaporation. Since the study did not include detailed water-use measurements or pot-weight-based evapotranspiration data, the authors should interpret these results as an association between microbial treatment and slower soil RWC decline under the experimental conditions.

Sixth, the data availability statement has improved because the authors now mention that relevant data are provided in the manuscript and Supporting Information. However, the authors should ensure that the S1 File contains the raw data underlying all tables, figures, statistical analyses, and correlations, not only summarized values. This is especially important because the manuscript reports many traits, treatment combinations, time points, and correlation analyses.

Finally, the English language still needs substantial improvement. Although the authors state that the manuscript has been proofread, several typographical and grammatical errors remain, including phrases such as “mycrorrhizal plants,” “Bacilus,” “root to shot signalling pathway,” and “to done the study.” These errors reduce readability and may cause confusion in the Methods and Results sections. The manuscript should undergo careful professional English editing before acceptance.

Reviewer #2: Although authours adequately addressed comments raised in a previous round of review. Their is need for major corrections in Data tables, results and discussion section.

1. In Table 7 (Correlation among root colonization and leaf physiobiochemical traits) Nine biologically distinct traits (water content, photosynthesis, phenols, proline, sugars, ascorbate, carotenoids, glutathione, ABA) all show |r| between 0.85–0.97, which is surprisgnly very high. Pls recheck the statestical analysis.

2. Letter groupings inconsistent with the stated LSD in several tables e.g. Tables 2, 3, and 6. Pls correct the same in all tables and rewrite the result accurately.

3. For RWC, the WS and WW columns show exactly the same SEm± (0.49) and LSD₀.₀₅ (1.45) to two decimal places. Two different measurement conditions (stressed vs. well-watered) coincidentally producing identical error variance is unlikely. Pls recheck.

4. "At just before imposing water-stress" is awkward — "before water-stress imposition" or simply "at BS" (as used elsewhere) would read more smoothly and stay consistent with your own abbreviation convention.

5. Several "statistically at par" claims in 3.9 (respiration) aren't supported by Table 4's own letter groupings. Check also 3.15 Carotenoids and 3.16 Glutathione section.

6. Correct the statements "Troyer seedlings had higher rate of leaf respiration than Cleopatra" (BS and WW), "Control plants suffered total stomatal closure".

7. The result section never cites treatment codes (T1, T6, etc.), forcing cross-referencing against each Table to verify each claim. Adding the code inline (e.g., "G. intraradices + PSB (T6)") would make the text auditable without reconstructing the mapping from numbers alone.

8. In 3.4 leaf area recheck the table and correct the result section e.g. Cleopatra G.intraradices+PSB leaf area under WW: change 18.90 → 25.23 cm² (it's a duplicated figure from an unrelated species/treatment). Add the missing Cleopatra control WW value: 15.24 cm², so the "at BS, WS, and WW" list has three control values, not two.

9. Discussion section needs substantial improvement. Each subsection (water relations, chlorophyll, gas exchange, osmolytes, antioxidants, ABA) should open by clearly restating your result with actual data (percent change, direction, statistical significance) before citing supporting/contrasting literature. Presently no numbers appear anywhere in this section, so the reader has no sense of effect magnitude.

10. Not a single p-value, percentage change, or magnitude is reported in the Discussion (e.g., "significantly higher chlorophyll concentration" — by how much?).

11. You worked with two genotypes (Troyer citrange and Cleopatra mandarin) but never discuss whether they responded differently to inoculation — this is scientifically interesting (differential drought tolerance/rootstock physiology) and currently unaddressed.

12. The paragraph on ABA is your most original mechanistic contribution (linking soil moisture retention/leaf RWC to reduced hydraulic triggering of ABA biosynthesis), but it's introduced almost incidentally at the end of a long paragraph. This deserves its own clearly flagged paragraph as a "novel interpretation," since it's the closest the section comes to proposing a mechanism rather than just corroborating past studies.

13. Break into subheadings (e.g., 4.1 Root colonization and water relations; 4.2 Photosynthetic pigments and gas exchange; 4.3 Osmotic adjustment; 4.4 Antioxidant response; 4.5 Hormonal regulation; 4.6 Limitations). The current single unbroken block is difficult to navigate for ~9 pages of text.

14. Typo: "chrorophyll" → "chlorophyll"

15. Fix bacterial treatment nomenclature throughout.

**********

what does this mean?). If published, this will include your full peer review and any attached files.

If you choose “no”, your identity will remain anonymous but your review may still be made public.

**Do you want your identity to be public for this peer review?**  For information about this choice, including consent withdrawal, please see our Privacy Policy

Reviewer #1: No

Reviewer #2: **Yes:**  Darshan M. Kadam

**********

[NOTE: If reviewer comments were submitted as an attachment file, they will be attached to this email and accessible via the submission site. Please log into your account, locate the manuscript record, and check for the action link "View Attachments". If this link does not appear, there are no attachment files.]

To ensure your figures meet our technical requirements, please review our figure guidelines: https://journals.plos.org/plosone/s/figures

You may also use PLOS’s free figure tool, NAAS, to help you prepare publication quality figures: https://journals.plos.org/plosone/s/figures#loc-tools-for-figure-preparation.

NAAS will assess whether your figures meet our technical requirements by comparing each figure against our figure specifications.

# Author comment: Author response to Decision Letter 2

**DOI:** 10.1371/journal.pone.0361406.r004

19 Sep 2026

Dear Handling Academic Editor,

We sincerely thank the Editor and the Reviewers for their careful evaluation and constructive comments. We have carefully considered all comments and incorporated the necessary corrections throughout the revised manuscript. For clarity, all the corrections made in the manuscript as responses to Reviewer #1 are shown in RED font and responses to Reviewer #2 and BLUE font.

Regards

Response to Reviewer #1

We thank you for your constructive, insightful, and highly valuable feedback on our manuscript. We have carefully addressed all major and minor comments. Below is our detailed, point-by-point response describing the exact changes made to the manuscript. All corresponding modifications are marked in RED font in the revised manuscript text.

Comment 1: The title and parts of the abstract have been improved, but language related to non-hydraulic root-to-shoot signalling remains. The study did not directly measure root ABA concentration, xylem sap ABA concentration, xylem sap flow, ABA transport, or stomatal sensitivity to ABA.

Response: We sincerely thank the Reviewer for this important clarification. We agree that the present study did not directly measure these components of ABA transport or signalling. Accordingly, all necessary corrections have been incorporated throughout the manuscript. Statements implying a demonstrated ABA-mediated non-hydraulic root-to-shoot signalling mechanism have been removed or appropriately toned down. The findings are now described primarily in terms of drought-associated physiological and biochemical responses and leaf ABA accumulation. Mechanistic interpretations concerning ABA-mediated root-to-shoot signalling are presented only as a possible hypothesis for future investigation.

Comment 2: The statistical analysis requires clarification because the manuscript states n = 6 after pooling across years, whereas the response letter mentions n = 12.

Response: We thank the Reviewer for identifying this inconsistency. The discrepancy has been corrected throughout the manuscript and response letter. The correct number of independent biological replications is n = 6, not n = 12. The pot was considered the experimental unit, with six seedlings maintained per pot. The statistical analyses were based on six independent biological replicates. All statements referring to n = 12 have been corrected to n = 6.

Comment 3: Methodological details concerning HPLC-UV validation for ABA measurement and the gridline-intersect method for AMF colonization should be clarified in the main Methods section.

Response: We appreciate this suggestion. The Methods section has been revised to provide the necessary practical details for reproducibility. The ABA analysis now includes the relevant extraction and HPLC-UV procedures, calibration/standard information and validation details. The AMF colonization procedure has also been clarified, including the number of root segments examined, application of the gridline-intersect method and calculation of percentage root colonization.

Comment 4: Concerns remain regarding controls and possible background colonization. The manuscript should describe substrate sterilization, mock inoculation, separation of controls and measures used to minimize contamination.

Response: We thank the Reviewer for this important point. The Methods and Results sections have been revised to describe substrate sterilization, control/mock inoculation procedures, separation of control plants and measures taken to minimize cross-contamination during the experimental period and transition to open conditions. The wording concerning AMF colonization in non-AM treatments has also been corrected to remain consistent with the small non-zero values reported in Table 1. The statement “No mycorrhizal colonization was observed” has therefore been avoided where it could conflict with the numerical observations.

Comment 5: Interpretation of rhizospheric soil relative water content should remain cautious and should not imply that microbial inoculation directly improved soil water retention.

Response: We agree with the Reviewer. The interpretation of soil relative water content has been revised throughout the manuscript. The results are now described as an association between microbial treatment and a slower decline in soil RWC under the experimental conditions, rather than as direct evidence that microbial inoculation independently improved soil water retention. Possible contributions of plant size, leaf area, transpiration, root biomass, substrate heterogeneity and evaporation are acknowledged.

Comment 6: The S1 File should contain the raw data underlying all tables, figures, statistical analyses and correlations.

Response: We appreciate the Reviewer’s recommendation. The Supporting Information has been checked/revised to ensure that the data underlying the reported analyses are provided in an appropriate form. The data availability statement has also been revised accordingly.

Comment 7: The English language still contains typographical and grammatical errors.

Response: We thank the Reviewer for pointing this out. The manuscript has undergone careful language editing. Typographical, grammatical and terminology-related errors have been corrected throughout, including “mycrorrhizal plants,” “Bacilus,” “root to shot signalling pathway,” and “to done the study.” The manuscript has also been checked for consistency in scientific terminology and treatment nomenclature.

Regards

Response to Reviewer #2

We thank you for your constructive, insightful, and highly valuable feedback on our manuscript. We have carefully addressed all major and minor comments. Below is our detailed, point-by-point response describing the exact changes made to the manuscript. All corresponding modifications are marked in Blue font in the revised manuscript text.

Comment 1: In Table 7, nine biologically distinct traits show very high correlations (|r| = 0.85–0.97). Please recheck the statistical analysis.

Response: We thank the Reviewer for carefully examining the correlation analysis. We have rechecked the statistical analysis and the underlying calculations for Table 7. The analysis is correct and no statistical error was identified. Table 7 has therefore been retained. The correlations are based on the observations used for the correlation analysis and are reported as calculated. The interpretation has also been kept strictly correlational and does not imply causality.

Comment 2: Letter groupings are inconsistent with the stated LSD in several tables, including Tables 2, 3 and 6.

Response: We appreciate the Reviewer’s careful checking. In response, SEm and LSD values have been deleted from the tables. The statistical grouping letters have been checked and corrected wherever necessary according to the underlying analysis, and the corresponding Results text has been revised to accurately reflect the statistical groupings.

Comment 3: For RWC, WS and WW columns show identical SEm and LSD values. Please recheck.

Response: We thank the Reviewer for highlighting this issue. The RWC statistical presentation has been rechecked. In the revised tables, SEm and LSD values have been deleted as requested. The treatment comparisons and statistical grouping letters have been checked and corrected according to the underlying analysis.

Comment 4: “At just before imposing water-stress” is awkward.

Response: We agree. The wording has been corrected throughout the manuscript and is now presented as “before water-stress imposition” or “at BS,” consistent with the abbreviation convention.

Comment 5: Several “statistically at par” claims in Section 3.9 are not supported by Table 4 groupings. Check Sections 3.15 and 3.16 as well.

Response: We thank the Reviewer for this careful observation. Sections 3.9, 3.15 and 3.16 have been thoroughly rechecked against the statistical grouping letters. Statements concerning treatments being “statistically at par” have been corrected wherever necessary, and the Results text now corresponds strictly with the reported statistical groupings.

Comment 6: Correct the statements “Troyer seedlings had higher rate of leaf respiration than Cleopatra” and “Control plants suffered total stomatal closure.”

Response: We agree. These statements have been corrected to avoid unsupported generalizations. The revised Results describe the observed treatment- and condition-specific differences without making blanket statements about genotype superiority or complete stomatal closure unless directly supported by the data.

Comment 7: The Results section should cite treatment codes such as T1 and T6 inline.

Response: We appreciate this useful suggestion. Treatment codes have been incorporated throughout the Results section along with the corresponding treatment descriptions, for example, “G. intraradices + PSB (T6),” making the Results easier to trace directly to the tables.

Comment 8: In Section 3.4, Cleopatra G. intraradices + PSB under WW should be 25.23 cm², and Cleopatra control under WW should be 15.24 cm².

Response: We thank the Reviewer for identifying these errors. The values have been corrected in the revised manuscript. The corresponding Results text has also been revised.

Comment 9: The Discussion requires substantial improvement, with actual data, percentage change, direction and statistical significance before literature comparison.

Response: We agree with the Reviewer. The Discussion has been substantially revised. The revised subsections now begin with the principal findings of the present study and, where appropriate, include the direction, magnitude and statistical significance of the observed changes before comparison with previous reports.

Comment 10: No p-value, percentage change or magnitude is reported in the Discussion.

Response: We thank the Reviewer for this suggestion. The Discussion has been revised to include quantitative information wherever appropriate, including percentage changes, effect magnitudes and statistical significance based on the reported analyses.

Comment 11: The manuscript does not discuss whether Troyer citrange and Cleopatra mandarin responded differently to inoculation.

Response: We agree that genotype-dependent responses are scientifically relevant. A dedicated discussion of Troyer citrange and Cleopatra mandarin has therefore been incorporated. The revised text compares the observed response patterns between the two rootstocks while avoiding conclusions not directly supported by the statistical analysis.

Comment 12: The ABA paragraph should be developed as a clearly identified novel interpretation.

Response: We appreciate this important suggestion. The ABA interpretation has been revised and given greater prominence in the Discussion. The revised text clearly distinguishes the observed association between soil moisture status, leaf water relations and leaf ABA accumulation from a demonstrated signalling mechanism. Because root ABA concentration, xylem ABA concentration, xylem sap flow and ABA transport were not directly measured, the proposed mechanistic interpretation is explicitly presented as a hypothesis for future investigation.

Comment 13: Break the Discussion into subheadings.

Response: We agree. The Discussion has been reorganized into clear thematic subsections: 4.1 Root colonization and water relations; 4.2 Photosynthetic pigments and gas exchange; 4.3 Osmotic adjustment; 4.4 Antioxidant response; 4.5 Hormonal regulation and ABA accumulation; 4.6 Genotype-dependent responses; and 4.7 Limitations and future perspectives.

Comment 14: Typo: “chrorophyll” should be “chlorophyll.”

Response: Corrected. The spelling “chrorophyll” has been replaced with “chlorophyll” throughout the manuscript, and the manuscript has been checked for similar typographical errors.

Comment 15: Fix bacterial treatment nomenclature throughout.

Response: We thank the Reviewer for this observation. Bacterial treatment nomenclature has been standardized throughout the manuscript, including the Methods, Results, Discussion, tables, figures and Supporting Information. Treatment names and codes are now used consistently.

Regards

General Response

We again thank both Reviewers for their constructive comments, which have helped us improve the clarity, statistical presentation, methodological reproducibility and scientific interpretation of the manuscript. All necessary corrections have been incorporated in the revised manuscript. As requested, SEm and LSD values have been removed from the tables, the correct replication number has been stated as n = 6, and Table 7 has been retained following rechecking of the correlation analysis.

Regards

Attachment Submitted filename: Response to Reviewers.docx

# Peer review: Decision Letter 2

**DOI:** 10.1371/journal.pone.0361406.r005

**Authors:** Eugenio Llorens

6 Oct 2026

Dear Dr. Khadivi,

Thank you for submitting your manuscript to PLOS One. After careful consideration, we feel that it has merit but does not fully meet PLOS One’s publication criteria as it currently stands. Therefore, we invite you to submit a revised version of the manuscript that addresses the points raised during the review process.

Please submit your revised manuscript by Nov 05 2026 11:59PM. If you will need more time than this to complete your revisions, please reply to this message or contact the journal office at plosone@plos.org. When you're ready to submit your revision, log on to https://www.editorialmanager.com/pone/ and select the 'Submissions Needing Revision' folder to locate your manuscript file.

- A letter that responds to each point raised by the academic editor and reviewer(s). You should upload this letter as a separate file labeled 'Response to Reviewers'.
- A marked-up copy of your manuscript that highlights changes made to the original version. You should upload this as a separate file labeled 'Revised Manuscript with Track Changes'.
- An unmarked version of your revised paper without tracked changes. You should upload this as a separate file labeled 'Manuscript'.

If you would like to make changes to your financial disclosure, please include your updated statement in your cover letter. Guidelines for resubmitting your figure files are available below the reviewer comments at the end of this letter.

If applicable, we recommend that you deposit your laboratory protocols in protocols.io to enhance the reproducibility of your results. Protocols.io assigns your protocol its own identifier (DOI) so that it can be cited independently in the future. For instructions see: https://journals.plos.org/plosone/s/submission-guidelines#loc-laboratory-protocols. Additionally, PLOS One offers an option for publishing peer-reviewed Lab Protocol articles, which describe protocols hosted on protocols.io. Read more information on sharing protocols at https://plos.org/protocols?utm_medium=editorial-email&utm_source=authorletters&utm_campaign=protocols.

As the corresponding author, your ORCID iD is verified in the submission system and will appear in the published article. PLOS supports the use of ORCID, and we encourage all coauthors to register for an ORCID iD and use it as well. Please encourage your coauthors to verify their ORCID iD within the submission system before final acceptance, as unverified ORCID iDs will not appear in the published article. *Only*  the individual author can complete the verification step; PLOS staff *cannot*  verify ORCID iDs on behalf of authors.

We look forward to receiving your revised manuscript.

Kind regards,

Eugenio Llorens

Academic Editor

PLOS One

Journal Requirements:

If the reviewer comments include a recommendation to cite specific previously published works, please review and evaluate these publications to determine whether they are relevant and should be cited. There is no requirement to cite these works unless the editor has indicated otherwise.

Please review your reference list to ensure that it is complete and correct. If you have cited papers that have been retracted, please include the rationale for doing so in the manuscript text, or remove these references and replace them with relevant current references. Any changes to the reference list should be mentioned in the rebuttal letter that accompanies your revised manuscript. If you need to cite a retracted article, indicate the article’s retracted status in the References list and also include a citation and full reference for the retraction notice.

[Note: HTML markup is below. Please do not edit.]

Reviewers' comments:

Reviewer's Responses to Questions

**Comments to the Author**

Reviewer #1: (No Response)

Reviewer #2: All comments have been addressed

**********

2. Is the manuscript technically sound, and do the data support the conclusions?

Reviewer #1: Yes

Reviewer #2: Yes

**********

3. Has the statistical analysis been performed appropriately and rigorously?

Reviewer #1: Yes

Reviewer #2: Yes

**********

4. Have the authors made all data underlying the findings in their manuscript fully available?

The PLOS Data policy

Reviewer #1: No

Reviewer #2: Yes

**********

5. Is the manuscript presented in an intelligible fashion and written in standard English?

Reviewer #1: No

Reviewer #2: Yes

**********

Reviewer #1: I appreciate the authors’ efforts to revise the manuscript and respond to the reviewers’ previous comments. The second revised version has improved in several respects. In particular, the title and keywords are now more appropriate, the strongest mechanistic claim regarding ABA-mediated non-hydraulic root-to-shoot signalling has been reduced in several parts of the manuscript, the statistical replication number has been clarified as n = 6, and additional information has been added regarding experimental controls, substrate sterilization, plant separation, and contamination prevention. The Discussion has also been reorganized into clearer subsections, and a limitations section has been retained. However, after reviewing the revised manuscript, I consider that several issues remain unresolved and should be corrected before acceptance.

First, although the authors state that all claims related to ABA-mediated root-to-shoot signalling have been removed or toned down, one problematic statement remains in the Results section. The manuscript still states that “G. intraradices + PSB dual inoculation resulted in significantly reduced level of ABA flux into leaf from root.” This statement is not supported by the measurements performed in the study. The authors measured leaf ABA concentration, but they did not directly measure root ABA concentration, xylem sap ABA concentration, xylem sap flow, ABA transport, or ABA flux. Therefore, this sentence should be revised to refer only to leaf tissue ABA concentration. For example, the authors could write: “G. intraradices + PSB dual inoculation resulted in significantly lower leaf tissue ABA concentration.”

Second, the methodological description of ABA quantification still requires correction. The authors added some information about extraction and HPLC-related procedures, but the description remains incomplete and partly unclear. In particular, the sentence “Standard monitoring was performed at” appears unfinished, and the detection wavelength and several key chromatographic details are missing. The authors should provide a complete and reproducible description of the HPLC-UV method, including detection wavelength, column type, mobile phase, flow rate, calibration procedure, ABA standard, recovery correction, and how final concentrations were calculated.

Third, the authors state in the response letter that the gridline-intersect method for AMF colonization was clarified. However, the main Methods section still mainly describes clearing/staining and counting colonized root bits among 30 root segments per replication. If the gridline-intersect method was actually used, the authors should describe it explicitly and consistently. If the authors used a root-bit counting method rather than a gridline-intersect method, the response letter and Methods section should be corrected accordingly.

Fourth, some language and typographical problems remain despite the authors’ statement that the manuscript was carefully edited. For example, errors such as “mycrorrhizal plants,” “Bacilus,” and “to done the study” are still present in the manuscript. These errors should be corrected, and the manuscript should undergo another round of careful English editing. In addition, several awkward phrases remain, such as “at just before given differential water treatment” and “statistically insignificant with,” which should be revised for clarity and standard scientific English.

Fifth, some unsupported or exaggerated statements remain in the Results and Discussion. For example, the manuscript still states that “control plants suffered total stomatal closure,” although the reported stomatal conductance values are low but not zero. This should be revised to a more accurate statement, such as “control plants showed significantly reduced stomatal conductance.” Similarly, the conclusion states that Troyer citrange performed as a superior rootstock compared to Cleopatra mandarin under water stress, but several genotype comparisons in the Discussion are described as statistically insignificant. Therefore, the conclusion should be more cautious and should avoid broad superiority claims unless directly supported by the statistical analysis.

Sixth, the correlation analysis in Table 7 still needs clearer explanation. The table reports correlations based on n = 24 treatment nodes, which suggests that correlations were calculated using treatment means rather than independent biological replicates. This should be clearly stated in the table note and interpreted cautiously. The current note is confusing because it states that each replication consisted of six plants as experimental units, whereas the statistical section states that the pot was the experimental unit. This should be harmonized to avoid ambiguity.

Overall, the manuscript has improved substantially compared with the previous version, and many of the requested revisions have been addressed. However, the revision is still incomplete. Before the manuscript can be considered acceptable, the authors should remove the remaining unsupported ABA flux claim, complete the HPLC-UV methodological description, clarify the AMF colonization method, correct the remaining English and typographical errors, revise unsupported overstatements, and clarify the interpretation of the correlation analysis. I therefore recommend further revision before acceptance.

Reviewer #2: Revised manuscript addressed all comments previously raised. It should be accepted for publication.

**********

what does this mean?). If published, this will include your full peer review and any attached files.

If you choose “no”, your identity will remain anonymous but your review may still be made public.

**Do you want your identity to be public for this peer review?**  For information about this choice, including consent withdrawal, please see our Privacy Policy

Reviewer #1: No

Reviewer #2: **Yes:**  Darshan M. Kadam

**********

[NOTE: If reviewer comments were submitted as an attachment file, they will be attached to this email and accessible via the submission site. Please log into your account, locate the manuscript record, and check for the action link "View Attachments". If this link does not appear, there are no attachment files.]

To ensure your figures meet our technical requirements, please review our figure guidelines: https://journals.plos.org/plosone/s/figures

You may also use PLOS’s free figure tool, NAAS, to help you prepare publication quality figures: https://journals.plos.org/plosone/s/figures#loc-tools-for-figure-preparation.

NAAS will assess whether your figures meet our technical requirements by comparing each figure against our figure specifications.

# Author comment: Author response to Decision Letter 3

**DOI:** 10.1371/journal.pone.0361406.r006

7 Oct 2026

3rd- Revised Manuscript

Dear Editor,

We express our sincere appreciation to the Editor and Reviewers for their constructive, analytical, and highly precise technical evaluations of our manuscript. We have systematically addressed every remaining item raised, specifically modifying descriptive vocabularies to accurately ground physiological inferences, completing chemical tracking specifications, and clarifying structural replication parameters. As a result, all ambiguities surrounding 'ABA flux,' rootstock comparisons, and independent experimental setups have been successfully corrected, providing absolute scientific alignment with our empirical datasets. Below is a detailed, point-by-point narrative of the specific improvements applied to the text.

Regards

Reviewer #1:

We express our sincere appreciation to Reviewer #1 for their constructive, analytical, and highly precise technical evaluations of our manuscript. We have systematically addressed every remaining item raised, specifically modifying descriptive vocabularies to accurately ground physiological inferences, completing chemical tracking specifications, and clarifying structural replication parameters. As a result, all ambiguities surrounding 'ABA flux,' rootstock comparisons, and independent experimental setups have been successfully corrected, providing absolute scientific alignment with our empirical datasets. Below is a detailed, point-by-point narrative of the specific improvements applied to the text.

SYSTEMATIC POINT-BY-POINT REVISIONS: REVIEWER #1

Point 1 (Quantification Boundary & Purging 'Flux' Claims)

Reviewer Comment: First, although the authors state that all claims related to ABA-mediated root-to-shoot signalling have been removed or toned down, one problematic statement remains in the Results section. The manuscript still states that 'G. intraradices + PSB dual inoculation resulted in significantly reduced level of ABA flux into leaf from root.' This statement is not supported by the measurements performed in the study. The authors measured leaf ABA concentration, but they did not directly measure root ABA concentration, xylem sap ABA concentration, xylem sap flow, ABA transport, or ABA flux. Therefore, this sentence should be revised to refer only to leaf tissue ABA concentration. For example, the authors could write: 'G. intraradices + PSB dual inoculation resulted in significantly lower leaf tissue ABA concentration.'

Author Response: The authors completely agree with this rigorous evaluation. Because direct mechanics of trans-sap transport, root core concentrations, or volumetric flow streams were not empirically monitored, the word 'flux' introduces unverified mechanistic overstatement. Accordingly, this phrase has been entirely extracted from Section 3.17 (Results) and replaced with precise vocabulary focusing strictly on our true empirical parameters—the quantified leaf tissue hormone values.

Manuscript Text Modifications:

Section 3.17 (Hormonal Regulation Summary) has been corrected as follows:

"Under well-watered conditions, G. intraradices + PSB dual inoculation resulted in significantly lower leaf tissue ABA concentrations in both Troyer and Cleopatra seedlings (T₆: 2.187 and T₁₈: 2.089 μg g⁻¹ F.W., respectively), which was statistically comparable to Troyer seedlings inoculated with G. intraradices + Providencia sp. (T₈: 2.672 μg g⁻¹ F.W.)."

Point 2 (Completing HPLC-UV Methodological Reproducibility)

Reviewer Comment: Second, the methodological description of ABA quantification still requires correction. The authors added some information about extraction and HPLC-related procedures, but the description remains incomplete and partly unclear. In particular, the sentence 'Standard monitoring was performed at' appears unfinished, and the detection wavelength and several key chromatographic details are missing. The authors should provide a complete and reproducible description of the HPLC-UV method, including detection wavelength, column type, mobile phase, flow rate, calibration procedure, ABA standard, recovery correction, and how final concentrations were calculated.

Author Response: We deeply regret the syntax error that left the baseline monitoring sentence broken and incomplete in the previous draft. Section 2.2 (Parameters recorded) has been extensively expanded to supply a complete, rigorous, and completely reproducible analytical description of our chromatographic parameters. This includes specific stationary phase column dimensions, isocratic mobile phase composition, precise tracking wavelengths, calibration steps, and standard matrix calculation variables.

Manuscript Text Modifications:

Section 2.2 (ABA Extraction & Analysis Sub-block) has been updated with the following full block:

"Chromatographic separation was achieved via an HPLC-UV system equipped with a reversed-phase C18 column (250 mm × 4.6 mm, 5 µm particle size). The mobile phase consisted of an isocratic mixture of methanol and 0.1% aqueous acetic acid (60:40, v/v) pumped at a constant flow rate of 1.0 mL/min. Standard monitoring and detection were performed at a fixed UV wavelength of 254 nm. Primary standard reference materials (≥98% purity, Sigma-Aldrich) were dissolved in chromatographically pure methanol to prepare a concentrated stock solution. Working standards were prepared via serial dilution to construct a 5-point external calibration curve. The baseline method recovery rate (R) was evaluated at 75.8 per cent. The final absolute concentration of ABA in the leaf tissue was calculated using the following formula:

ABA content in leaf (µg g⁻¹ F.W.) = (Xvial × V) / (W × R) * 100

where Xvial = Concentration in the HPLC vial (μg/mL), V = Final volume of the reconstituted extract (mL), W = Fresh weight of the leaf tissue (g), and R = Method Recovery Rate expressed as a fraction (0.758)."

Regards

Point 3 (Clarification of Root Infection Mapping Methodology)

Reviewer Comment: Third, the authors state in the response letter that the gridline-intersect method for AMF colonization was clarified. However, the main Methods section still mainly describes clearing/staining and counting colonized root bits among 30 root segments per replication. If the gridline-intersect method was actually used, the authors should describe it explicitly and consistently. If the authors used a root-bit counting method rather than a gridline-intersect method, the response letter and Methods section should be corrected accordingly.

Author Response: We appreciate this crucial corrective point. The previous response letter inadvertently included conflicting procedural nomenclature. Our research group executed a root-bit segment counting method rather than the gridline-intersect protocol. To establish clear procedural alignment, all text frames within Section 2.2 have been systematically verified to describe the root-bit segment counting approach uniformly, detailing the specific scanning steps for the 30 segments evaluated per replicate.

Manuscript Text Modifications:

The descriptive text in Section 2.2 (Root colonization analysis) has been structured as follows:

"The presence of fungal hyphae, arbuscules, and vesicles was examined under a 10X compound microscope. For each biological replication, 30 distinct root segments (each 1 cm long) were meticulously scanned using a modified root-bit segment counting method to verify structural infection parameters. The total per cent root colonisation was calculated using the following formula: Percent root colonization = (Number of root bits positive for colonization / Total number of root bits observed under microscope) × 100."

Point 4 (Rectifying Language, Terminology, and Typographical Errors)

Reviewer Comment: Fourth, some language and typographical problems remain despite the authors’ statement that the manuscript was carefully edited. For example, errors such as 'mycrorrhizal plants,' 'Bacilus,' and 'to done the study' are still present in the manuscript. These errors should be corrected, and the manuscript should undergo another round of careful English editing. In addition, several awkward phrases remain, such as 'at just before given differential water treatment' and 'statistically insignificant with,' which should be revised for clarity and standard scientific English.

Author Response: We apologize for these technical copy-editing oversights. The entire manuscript text has undergone an exhausting language cleanup and restructuring process. All identified typos have been corrected ('mycrorrhizal' to 'mycorrhizal'; 'Bacilus' to 'Bacillus'; institutional permission altered from 'to done the study' to 'to conduct the study'). Furthermore, the unscientific expression 'statistically insignificant with' has been replaced throughout all text descriptions and data table annotations with standard scientific phrases, such as 'statistically comparable to' or 'did not differ significantly from.' Non-idiomatic chronological placeholders have also been streamlined.

Manuscript Text Modifications:

Global substitutions were executed across the master document:

- All instances of 'at just before given differential water treatment' -> 'just prior to the imposition of differential moisture regimes' or 'before stress (BS)'.

- All instances of 'statistically insignificant with' -> 'statistically comparable to' or 'did not differ significantly from'.

- Re-checked and corrected the spelling of the genus name Bacillus and the adjective mycorrhizal in all locations.

Point 5 (Softening Stomatal Overstatements and Genotype Rootstock Claims)

Reviewer Comment: Fifth, some unsupported or exaggerated statements remain in the Results and Discussion. For example, the manuscript still states that 'control plants suffered total stomatal closure,' although the reported stomatal conductance values are low but not zero. This should be revised to a more accurate statement, such as 'control plants showed significantly reduced stomatal conductance.' Similarly, the conclusion states that Troyer citrange performed as a superior rootstock compared to Cleopatra mandarin under water stress, but several genotype comparisons in the Discussion are described as statistically insignificant. Therefore, the conclusion should be more cautious and should avoid broad superiority claims unless directly supported by the statistical analysis.

Author Response: We recognize the accuracy of the reviewer's comment. Because stomatal conductance metrics in the uninoculated control treatments were minimized but mathematically remained above zero, utilizing the phrase 'total closure' is factually incorrect. We have reworded this sentence to state 'significantly reduced stomatal conductance.' Additionally, because specific dual-inoculated parameters between the two rootstocks showed cross-over statistical parities, we have carefully re-phrased our abstract and primary conclusion blocks to frame the performance of Troyer citrange relative to the specific physiological, hydraulic, and carbon assimilation matrices evaluated, avoiding generalized superiority claims.

Manuscript Text Modifications:

1. Section 3.10 (Stomatal behaviors text): "Conversely, uninoculated control plants showed significantly reduced stomatal conductance, which led to marked photosynthetic inhibition."

2. Section 5 (Conclusions): "Based on the monitored water relations and carbon assimilation matrices, Troyer citrange rootstock demonstrated a more functional physiological buffer profile under water deficit stress than Cleopatra mandarin."

3. Abstract (Final Sentence): "Under these experimental conditions, Troyer citrange exhibited higher relative water content, photosynthetic activity, and stomatal conductance under drought than Cleopatra mandarin, offering a potential microbial intervention strategy..."

Point 6 (Clarifying the 'Individual Potted Seedling' Experimental Unit & Correlation Matrix)

Reviewer Comment: Sixth, the correlation analysis in Table 7 still needs clearer explanation. The table reports correlations based on n = 24 treatment nodes, which suggests that correlations were calculated using treatment means rather than independent biological replicates. This should be clearly stated in the table note and interpreted cautiously. The current note is confusing because it states that each replication consisted of six plants as experimental units, whereas the statistical section states that the pot was the experimental unit. This should be harmonized to avoid ambiguity.

Author Response: The reviewer’s observation is entirely accurate. The Pearson correlation values were calculated strictly from the pooled treatment mean nodes (n = 24 total nodes) to illuminate broad physiological and biochemical co-regulatory trajectories across the various treatment matrices. We have updated Section 3.18 to state this clearly.

Furthermore, we have resolved the layout ambiguity across the Methods, Results, and Table Footnotes: our layout featured six seedlings grown individually in six separate pots per replication (one uniform seedling per pot), where each individual pot and its seedling functioned as a distinct, independent experimental unit (yielding 12 pots per treatment per year, compiled to n = 6 true biological replicates across the pooled three-year dataset). All sections have been fully synchronized with this definition to eliminate confusion.

Manuscript Text Modifications:

1. Section 2.1 (Experimental Setup layout text): "Each replication consisted of six separate pots, with one uniform seedling grown per pot, where each individual potted seedling served as a distinct independent experimental unit (yielding 12 pots per treatment per year, and a pooled sample size of n = 6 true biological replicates per treatment combination across the three years)."

2. Section 3.18 (Correlation Text update): "These coefficients were evaluated across the 24 treatment mean nodes to track global physiological trajectories across the treatments."

3. Table 7 Footnote (Harmonized text): "Note: Correlations were calculated using the treatment mean nodes (n = 24) derived from pooled annual runs. Within the experimental setup, each individual potted seedling served as a distinct independent experimental unit, yielding six true biological replications per treatment across the pooled dataset. Significance indicators: * details p < 0.05; ** details p < 0.01 (SPSS 16.0)."

Reviewer #2:

We express our sincere appreciation to Reviewer #2 for evaluating and accepting of our manuscript.

Reviewer Comment: Revised manuscript addressed all comments previously raised. It should be accepted for publication.

Author Response: We sincerely thank Reviewer #2 for reviewing our structural amendments and validating our scientific updates. We are grateful for the endorsement for publication.

Attachment Submitted filename: Response_to_Reviewers_auresp_3.docx

# Peer review: Decision Letter 3

**DOI:** 10.1371/journal.pone.0361406.r007

**Authors:** Eugenio Llorens

12 Oct 2026

<p>Arbuscular mycorrhizal fungi and mycorrhizal helper bacteria co-inoculation alters drought-associated physiological traits and leaf abscisic acid concentration in citrus rootstocks

PONE-D-26-27560R3

Dear Dr. Khadivi,

We’re pleased to inform you that your manuscript has been judged scientifically suitable for publication and will be formally accepted for publication once it meets all outstanding technical requirements.

Within one week, you’ll receive an e-mail detailing the required amendments. When these have been addressed, you’ll receive a formal acceptance letter and your manuscript will be scheduled for publication.

An invoice will be generated when your article is formally accepted. Please note, if your institution has a publishing partnership with PLOS and your article meets the relevant criteria, all or part of your publication costs will be covered. Please make sure your user information is up-to-date by logging into Editorial Manager at Editorial Manager® and clicking the ‘Update My Information' link at the top of the page. For questions related to billing, please contact billing support.

If your institution or institutions have a press office, please notify them about your upcoming paper to help maximize its impact. If they’ll be preparing press materials, please inform our press team as soon as possible -- no later than 48 hours after receiving the formal acceptance. Your manuscript will remain under strict press embargo until 2 pm Eastern Time on the date of publication. For more information, please contact onepress@plos.org.

Kind regards,

Eugenio Llorens

Academic Editor

PLOS One

Additional Editor Comments (optional):

Reviewers' comments:

Reviewer's Responses to Questions

**Comments to the Author**

Reviewer #1: All comments have been addressed

**********

2. Is the manuscript technically sound, and do the data support the conclusions?

Reviewer #1: Yes

**********

3. Has the statistical analysis been performed appropriately and rigorously?

Reviewer #1: Yes

**********

4. Have the authors made all data underlying the findings in their manuscript fully available?

The PLOS Data policy

Reviewer #1: Yes

**********

5. Is the manuscript presented in an intelligible fashion and written in standard English?

Reviewer #1: Yes

**********

Reviewer #1: The authors have adequately addressed my previous concerns. The unsupported ABA-flux wording has been removed, the ABA/HPLC method and AMF colonization method have been clarified, and the main overstatements have been revised. I am satisfied with the current version and recommend acceptance.

**********

what does this mean?). If published, this will include your full peer review and any attached files.

If you choose “no”, your identity will remain anonymous but your review may still be made public.

**Do you want your identity to be public for this peer review?**  For information about this choice, including consent withdrawal, please see our Privacy Policy

Reviewer #1: **Yes:**  Ali Mokhtassi-Bidgoli

**********

# Editor report: Acceptance letter

**DOI:** 10.1371/journal.pone.0361406.r008

**Authors:** Eugenio Llorens

PONE-D-26-27560R3

PLOS One

Dear Dr. Khadivi,

I'm pleased to inform you that your manuscript has been deemed suitable for publication in PLOS One. Congratulations! Your manuscript is now being handed over to our production team.

At this stage, our production department will prepare your paper for publication. This includes ensuring the following:

* All references, tables, and figures are properly cited

* All relevant supporting information is included in the manuscript submission,

* There are no issues that prevent the paper from being properly typeset

You will receive further instructions from the production team, including instructions on how to review your proof when it is ready. Please keep in mind that we are working through a large volume of accepted articles, so please give us a few days to review your paper and let you know the next and final steps.

Lastly, if your institution or institutions have a press office, please let them know about your upcoming paper now to help maximize its impact. If they'll be preparing press materials, please inform our press team within the next 48 hours. Your manuscript will remain under strict press embargo until 2 pm Eastern Time on the date of publication. For more information, please contact onepress@plos.org.

You will receive an invoice from PLOS for your publication fee after your manuscript has reached the completed accept phase. If you receive an email requesting payment before acceptance or for any other service, this may be a phishing scheme. Learn how to identify phishing emails and protect your accounts at https://explore.plos.org/phishing.

If we can help with anything else, please email us at customercare@plos.org.

Thank you for submitting your work to PLOS One and supporting open access.

Kind regards,

PLOS One Editorial Office Staff

on behalf of

Dr. Eugenio Llorens

Academic Editor

PLOS One
